# How do we sleep while our beds are burning? High ambient temperatures are associated with substantial sleep loss

**DOI:** 10.1093/sleep/zsaf323

**Published:** 2025-10-13

**Authors:** Bastien Lechat, Barbara Toson, Hannah Scott, Duc Phuc Nguyen, Billingsley Kaambwa, Amy C Reynolds, Jack Manners, Robert J Adams, Jean-Louis Pepin, Sebastien Bailly, Andrew J K Phillips, Pierre Escourrou, Peter Catcheside, Danny J Eckert

**Affiliations:** Flinders Health and Medical Research Institute: Sleep Health, College of Medicine and Public Health, Flinders University, Adelaide, SA, Australia; Flinders Health and Medical Research Institute: Sleep Health, College of Medicine and Public Health, Flinders University, Adelaide, SA, Australia; Flinders Health and Medical Research Institute: Sleep Health, College of Medicine and Public Health, Flinders University, Adelaide, SA, Australia; Flinders Health and Medical Research Institute: Sleep Health, College of Medicine and Public Health, Flinders University, Adelaide, SA, Australia; Health Economics Unit, College of Medicine and Public Health, Flinders University, Health Sciences Building, Sturt Road, Bedford Park, SA 5042, Australia; Flinders Health and Medical Research Institute: Sleep Health, College of Medicine and Public Health, Flinders University, Adelaide, SA, Australia; Flinders Health and Medical Research Institute: Sleep Health, College of Medicine and Public Health, Flinders University, Adelaide, SA, Australia; Flinders Health and Medical Research Institute: Sleep Health, College of Medicine and Public Health, Flinders University, Adelaide, SA, Australia; HP2 Laboratory, Univ. Grenoble Alpes, Inserm U-1300, CHU Grenoble Alpes, 38043 Grenoble, France; HP2 Laboratory, Univ. Grenoble Alpes, Inserm U-1300, CHU Grenoble Alpes, 38043 Grenoble, France; Flinders Health and Medical Research Institute: Sleep Health, College of Medicine and Public Health, Flinders University, Adelaide, SA, Australia; Centre Interdisciplinaire du Sommeil, Paris, France; Flinders Health and Medical Research Institute: Sleep Health, College of Medicine and Public Health, Flinders University, Adelaide, SA, Australia; Flinders Health and Medical Research Institute: Sleep Health, College of Medicine and Public Health, Flinders University, Adelaide, SA, Australia

**Keywords:** climate change, sleep, temperature, heat, big data, environmental stress

## Abstract

**Study Objectives:**

High ambient temperatures are associated with negative health outcomes, including heat stress, injuries and accidents, and poor mental health. Sleep loss may contribute to these adverse impacts. However, robust supporting evidence is lacking.

**Methods:**

Data from a global sample of sleep tracker users (*N* = 317 758; Withings Sleep Analyzer [WSA]: *n* = 116 879; Withings ScanWatch: *n* = 200 879) between January 2020 and September 2023 (~165 million nights) were analyzed. Ambient temperatures (24 hour average) were extracted from a climate model for each nightly observation based on the users’ location. We used the case-time-series design with participant–year–week intercept and spline functions to derive exposure–response curves between temperature and short sleep (<6 hours/night) prevalence, adjusting for other time-varying factors and meteorological variables.

**Results:**

High temperatures (99th vs 50th percentile of the observed global distribution; 27.3°C vs 12.2°C) were associated with (mean [95%CI]) −15.2 [−15.6, −14.9] and −16.9 [−17.4, −16.4] minute sleep loss in the users of the smartwatch and under-mattress sensors, respectively. Similarly, high temperatures were associated with an approximately 40% relative increase in the probability of short sleep on the same night (WSA: Risk Ratio: RR [95%CI]; 1.40 [1.38, 1.41]; ScanWatch: 1.43 [1.41, 1.44]). Estimates ranged between 10% and 75% depending on the country. Sleep loss to high temperatures was higher in participants residing in Europe and countries with lower national gross domestic product per capita.

**Conclusions:**

High temperatures negatively impact sleep duration and increase the probability of short sleep globally. Our findings suggest that rising temperatures may increase the health impacts of short sleep.

**Statement of Significance**

High ambient temperatures are linked to negative health outcomes, with sleep loss potentially contributing to these effects. This study analyzed data from 317 758 global sleep tracker users to examine the relationship between temperature and short sleep. Results showed an approximately 40% relative increase in the probability of short sleep at high temperatures (99th vs 50th percentile; 27.3°C vs 12.2°C) globally. Sleep loss to high temperatures was higher in participants residing in countries with lower national gross domestic product per capita and older adults. These results suggest that sleep inadequacy from rising temperature due to climate change may further amplify global inequalities. Our findings also highlight the urgent need for targeted strategies to mitigate temperature-induced sleep loss.

## Graphical Abstract

**Graphical Abstract:**
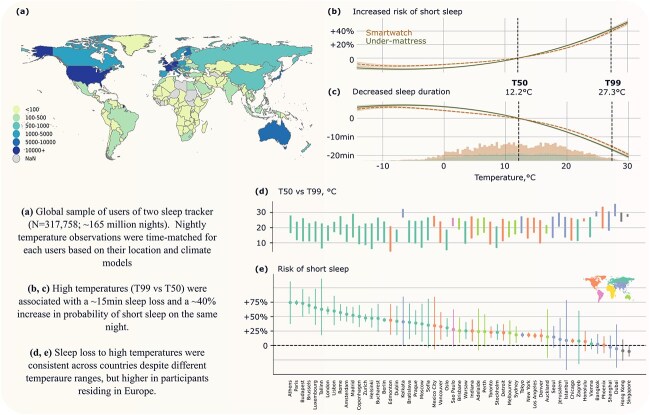

## Introduction

The Northern Hemisphere summer of 2023 saw the highest average temperatures in over 2000 years [1]. Temperatures were 2.07°C warmer than in the period 1850–1900, exceeding natural climate variability by more than 1°C [1]. High ambient temperatures and extreme heat events have strong negative effects on health [2, 3]. Increased temperatures are associated with increased risk of all-cause mortality, cardiovascular mortality, suicide, injuries (e.g. self-harm, falls, burns), and accident-related mortality [4–8]. Extreme heat also has negative impacts on an individual’s well-being, mental health [9, 10], and physical activity [11]. These impacts present major societal and labor loss costs [12], as well as increased healthcare and hospitalization costs [13]. Thus, negative health and societal impacts of high environmental temperatures have already occurred and are expected to keep rising without further adaptation.

Many adverse health outcomes related to extreme heat exposure are also associated with inadequate (i.e. disrupted/poor quality) and short sleep [14]. Sleep serves vital functions, including tissue repair and recovery, growth, memory retention, mood regulation, and optimal cardiac, metabolic, and immune system functions [15]. Consequently, the impacts of inadequate and short sleep include neurocognitive impairment and poor performance [16], poor mental health [17], and dysregulation of glucose metabolism [18] and immune function [19]. Inadequate sleep is also a major source of morbidity, mortality, and societal cost through traffic and work accidents, lost productivity, and related adverse health effects [14]. The evidence of an association between short sleep and poor health is particularly strong and consistent for people with sleep duration below 6 hours [14].

Despite the known effects of inadequate and insufficient sleep on well-being and performance, current estimates indicate that 30%–45% of adults do not achieve the recommended 7–9 hours of sleep per night, with 5%–30% of adults regularly sleeping less than 6 hours per night [14, 20–22]. Sleep initiation and maintenance are affected by many factors, including demographic and socioeconomic factors, comorbid health conditions, and sleep disorders [14, 15]. Human laboratory studies have also demonstrated the negative impact of high ambient temperature on sleep [23, 24]. Findings from a recent systematic review further support the hypothesis that higher ambient temperatures are associated with poorer sleep quality and quantity [25]. However, the impact of ambient temperature on human sleep has received little attention compared to other health-related impacts [26, 27]. Previous studies were either cross-sectional, were conducted in a single country, used devices unvalidated with polysomnography, or used self reported sleep duration [25].

In this study, we estimated exposure–response curves between ambient temperature and sleep loss using data from 317 758 participants who regularly used a validated wearable sensor (Withings Scan Watch, smartwatch) or a contactless under-mattress sleep sensor [28–31] (Withings Sleep Analyzer) between January 2020 and September 2023.

## Methods

### Participants

Retrospective data were acquired from 125 295 and 238 550 users who registered to use an under-mattress sleep sensor and/or a smartwatch (Withings Sleep Analyzer and Withings ScanWatch, respectively) between January 2020 and September 2023. Participants were required to be 18 years or older to be included in this study. Participants were also required to have a minimum of 28 nights of data and an average of at least four sleep recording nights per week to be included in the analysis. Participants were geo-localized by Withings to the closest largest city in each time zone within a country (if multiple time zones were present) or to the largest city if there was only a single time zone within a country and provided to the investigators. A more precise location was not available due to concern for reidentification and privacy. When participants sign up to the Withings app, they are prompted to fill in their age, sex, height, and weight. Information on gender is not collected. Information on other sociodemographic and clinical conditions is also not collected. All the participants provided written consent through the Withings app for their deidentified data to be used for research purposes when signing up for a Withings account. The study was approved by the Flinders University Human Research Ethics Committee (project number: 4291).

### Country-level information

We collected values for country-level gross domestic product (GDP) per capita from the World Bank (https://data.worldbank.org/indicator/NY.GDP.PCAP.CD). In some analyses, tertiles of national GDP per capita (calculated across participants) were used to assess the potential effect of high-income versus lower-income countries on the association between ambient temperature and sleep loss. The socio-development index (SDI), a measure of economic and societal development of countries developed by the World Health Organization and the Global Burden of Disease, was extracted from the 2019 estimates [32], and we used existing categories from the Global Burden of Disease to classify a country as “high,” “middle high,” or “middle” SDI.

### Device-based sleep monitoring

The Withings Sleep Analyzer is a sleep monitoring device placed under the mattress that estimates sleep timing and quality. This is achieved via automated proprietary algorithms from a built-in microphone and ballistographic assessment of movement, heart rate, and respiratory motion from a pressure sensor [30]. The Withings Sleep Analyzer has been validated against polysomnography in multiple independent studies [28–31]. The Withings Sleep Analyzer is comparable in sleep–wake classification accuracy to other validated consumer sleep monitors and research actigraphy-based devices [31, 33], and tends to overestimate sleep duration by approximately 30 minutes on average compared to polysomnography [29, 30]. The Withings ScanWatch is a wrist-worn device with a three-axis accelerometer and photoplethysmography sensor enabling it to track activity, sleep, and heart rate, although it has not been as extensively validated as the Withings Sleep Analyzer. The Withings ScanWatch was chosen as the reference wearable in the Sleep Revolution project [34], a multicenter project focusing on sleep disorder and sleep health technology. The Sleep Revolution consortium have validated the device, although it has not been peer reviewed (https://skemman.is/handle/1946/39300). A previous watch (Withings Activite) from the same company has been validated against polysomnography [35]. We compared averaged sleep duration in a subset (*N* = 3865) of users that owned both devices (see “device comparison” section in Supplementary Results). Overall, sleep duration was also approximately 21 minutes higher when people used the smartwatch vs the under-mattress sensors, despite similar sleep onset and time out of bed. Wake after sleep onset was 13 minutes lower when people used the smartwatch versus the under-mattress sensors (see Supplementary Tables S2 and S3).

### Assessment of weather information

We time-matched weather information for each day of data for every user based on their closest largest city within their time zone and country, collected from the Copernicus Climate Change Service [36]. We extracted hourly air temperature at 2 m from the ground for each of the main cities (square of 500 × 500 km around the location) in the user database for the period 2020–2023 from the fifth generation of the European Reanalysis (ERA5) dataset [37] using the Copernicus Climate Change Service [36]. The ERA5 is a climate reanalysis product and offers land-surface data that have previously been shown to provide a satisfactory proxy to station-based series and used extensively in assessing the effect of ambient temperature on health [38, 39]. Dates and time in the ERA5 dataset were converted to local time for each city. Minimum, maximum, and mean 24 hour temperatures (midnight to midnight) were calculated for each participant and location, averaged over the 500 × 500 km square around the location, for each day where sleep was recorded. We also extracted the hourly dew point temperature, total cloud cover, total precipitation, and surface pressure for each location, which were subsequently averaged over each 24 hour period. Relative humidity was calculated using MetPy [40] based on an existing formula [41].

### Assessment of air pollution

We extracted the fine particulate matter (aerodynamic diameter < 2.5 μm) concentration from the European Centre for Medium-Range Weather Forecasts Atmospheric Composition Reanalysis 4 model as a measure of air quality [42]. These reanalysis models have been validated against station-based measurements [38, 39, 43] and used in previous environmental health research [38, 39]. Air pollution was time-matched to each participant using the same methodology and grid size (500 × 500 km) as the weather information.

### Heatwave definition

Heatwaves were identified for each of the main cities between January 2020 and September 2023 using pre-existing methods [44]. A heatwave was detected when at least three consecutive days were above the 90th percentile for each calendar day at a given location. The 90th percentile was based on a 15 day moving window of daily 24 hour average temperatures over the 1950–1990 period for a given location. Average historical temperature was defined as the 50th percentile, similarly, calculated as a 15 day rolling 24 hour average. Two additional criteria were imposed for heatwave classification: an average temperature during the heatwave of at least 15°C (based on the main analysis) and an increase compared to baseline (average between the 7–14 days prior to the maximum of the heatwave) of at least 1°C. Sleep and heatwaves were time-matched to the peak of the heatwaves *(t*_0_).

### Statistical analysis

We followed the recently proposed case-time-series design [45], to study the effects of 24 hour average ambient temperature on sleep duration and short sleep, defined as a sleep duration of less than 6 hours. These research designs have been used in multiple studies to assess the effects of environmental factors on health [46, 47]. For a given outcome ${y}_{it}$, here either continuous sleep duration modeled with a Gaussian family and identity link function or short sleep (<6 hours) modeled using a binomial family and logit link function, for a person *i* at a time *t*, the equation for the case-time-series design framework can be written as follows:


(1) \begin{equation*} g\left[E\left({y}_{it}\right)\right]={\mu}_{i(k)}+f\left({x}_{it},l\right)+\sum_{j=1}^j{s}_j(t)+\sum_{p=1}^p{h}_p\left({z}_{ipt}\right)+{\varepsilon}_{it} \end{equation*}

The regression is efficiently performed using the general framework of fixed-effects models [48]. Here, $g\left[E\left({y}_{it}\right)\right]$ represents the transformation using a link function $g$ (identity or logit) of the expected values of our outcome variable ${y}_{it}$. The function $f\left({x}_{it},l\right)$ specifies the associations between the exposure of interest ${x}_{it}$ (24 hour average temperature at a time *t* for a person *i*) and our outcome of interest. Here, in our primary model, the association of interest between temperature and sleep duration (or short sleep) was modeled using distributed lags [4, 49], similar to other environmental health epidemiology studies [5, 7, 50, 51]. Lagged models were chosen because they can describe complex nonlinear exposure–response associations such that any potential delayed effects (e.g. temperature on a given day impacting sleep 2 days later) can be assessed. We chose a 4 day lag effect, but other lag structures were also tested (see Supplementary Figures S1 and S2). Temperature was modeled using a natural spline with 4 degrees of freedom, and the lag–response function used two knots. The terms ${s}_j(t)$ represent functions expressed at different timescales to model temporal variations in risk. In our primary model, this included natural splines of time (day of year variable; with 4 degrees of freedom [df]), and indicators of the day of the week. Other potential time-varying confounders ${z}_{ipt}$ can be modeled using the function ${h}_p$, here a natural spline with no lag. In our model, time-varying confounders ${z}_{ipt}$ included total cloud cover (4 df), relative humidity (4 df), density of particulate matter with aerodynamic diameter less than 2.5 μg/m^3^ (4 df), surface pressure (4 df), and total precipitation (4 df). Finally, the intercept ${\mu}_{i(k)}$ expresses baseline risk for different participants *i* with further nested time-strata *k*, thus allowing within-individual variation in baseline risks along the time stratum. For example, in our model we used participant/year/week strata intercepts, which would capture between-participant differences such as sociodemographic or medical conditions that do not vary with time (in the “participant” part of the intercept). This intercept, through the “year/week” would also allow for varying baseline risk for each week of each year for each participant, thus also accounting for time-varying confounders. For example, seasonal effect and/or average daylight effect on sleep duration [25, 52] would be accounted for *at the individual level* week to week. This intercept would also account for school holidays’ effect on sleep duration, or poor sleep due to exacerbation of symptoms due to a medical condition, such as chronic obstructive pulmonary disease [53]. The association between temperature and sleep duration was then summarized by cumulating the effect over the lag period, similar to previous studies [4, 5, 7, 49–51]. This approach was undertaken on the full dataset, and in each city separately. Further analyses examined effects in specific subgroups of interest, including age groups (10 year bins), sex, habitual sleep duration (average sleep duration over the recording period), tertile of national GDP per capita, and tertile of SDI. The models were implemented in the R programming language, using the dlnm [54] and gnm [55] package, and the full model specifications can be found at https://github.com/bastienlechat/OSA_climate.

We used a similar approach to the main analysis to study the potential associations between heatwaves and sleep loss and short sleep. Participant/year/month strata intercepts were included in the model, as well as an indicator of the day of the week, to model time-varying confounding factors. The exposure of interest (number of days relative to the maximum of the heatwave) was modeled using natural splines with 6 degrees of freedom, adjusted for the average historical temperature between 1950 and 1990 (city-specific for each calendar day; modeled using natural splines with 4 days of freedom).

### Alternative model specifications

We ran several additional models to further assess the robustness of our findings. In our main model, since we have temporal adjustment for the day of the year within the country-by-country analysis, the national average effect of seasonal daylight variation on sleep duration should have been adjusted for. We further validated this assumption in our model by constructing an alternative model specifically adjusted for daylight duration rather than the day of the year. Daylight duration was calculated using the Astral Python package based on users’ location and date of measurement. We also tested two alternative models with different temporal control variables. One model was adjusted for the person–year–week stratum and incorporated a natural cubic spline of time (6 degrees of freedom per year), similar to a previous study [47]. Another alternative model tested the effect of different strata intercepts, namely person–year–month versus person–year–week. We constructed three additional alternative models testing different functions to model the exposure of interest $f\left({x}_{it},l\right)$, namely: (1) a spline function with 4 degrees of freedom but no lagged effect, (2) a linear model with lagged effect (4 day lag effect), and (3) a linear model with no lag. These alternatives models were chosen to compare our model specification to previous studies [25, 26, 56].

### Sensitivity analyses

We undertook several additional sensitivity analyses to further validate our findings with slightly different versions of the dataset. In the first sensitivity analysis, we used more conservative inclusion/exclusion criteria of at least 26, 52, or 104 weeks of data. This sensitivity analysis had a longer temporal resolution and coverage of expected seasonal effects within each participant. In the second sensitivity analysis, we adopt additional inclusion criteria based on minimum and maximum allowable sleep duration (between 4 and 12 hours) based on previous global observational studies on wearable sleep technologies [26, 57]. The COVID-19 pandemic impacted sleep, especially during lockdowns [58, 59]. Therefore, in the third sensitivity analysis, we reproduced the analysis only in data after September 2022, a period where COVID-19 was less likely to confound the observed results. Air pollution and high ambient temperatures have been reported to be correlated [60, 61] and previous studies have suggested a potential synergistic effect between the two variables and health outcomes such as mortality [60, 61]. Given this and the potential for multicollinearity between the environmental variables, we repeated the analysis using a minimally adjusted model with only a participant–year–week stratum and a day of the year and day of the week variable. Finally, we conducted an additional sensitivity analysis to test the robustness of the spline functions to model the exposure of interest $f\left({x}_{it},l\right)$, by specifying different degrees of freedom (3, 6, and 8 df).

## Results

**Figure 1:**
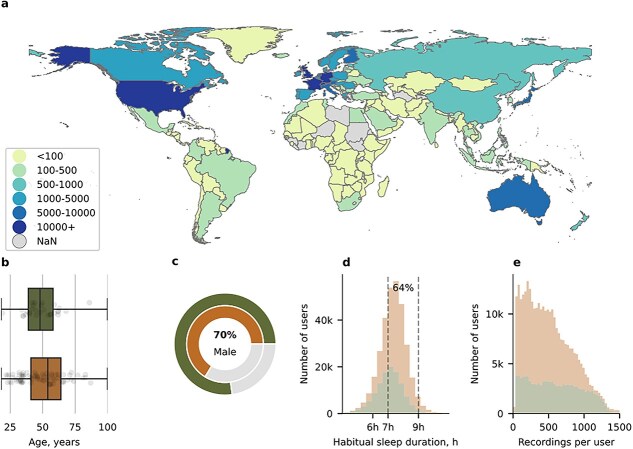
(a) Geographic distribution of the study population (both devices combined). (b) Age distribution of smartwatch (green) versus under-mattress (orange) users. (c) Proportion of male versus female (same color scheme applies). Middle number represents the proportion of males in the combined dataset (both devices). (d) Habitual sleep duration (average over the recording period) for both devices (the number on the top indicates the percentage of people who slept between 7 and 9 hours [14] in the combined dataset) and (e) number of recordings per participant for each device.

Data were analysed from 317 758 users (87% of the total sample), including 116 879 users of the under-mattress sleep sensor and 200 879 smartwatch users, who regularly used their sleep-tracking device between January 2020 and September 2023 for a combined total of approximately 165 million nights. Flow charts and inclusion criteria are detailed in Methods and Supplementary Figures S3 and S4. Users were located in 198 regions (Figure 1), including 41 countries and 50 countries with at least 100 under-mattress sensor and smartwatch users, respectively (Supplementary Table S1). Participants had a mean of 464 nights (median [IQR]; 464 [234, 743]) of sleep recordings. Population demographics (age and sex) varied somewhat between under-mattress versus smartwatch users (Figure 1b and c; Supplementary Table S1), but were consistent across the different countries. Globally, 64% of the sample had an average sleep duration within the 7–9 hour range. A total of 11% and 4% of the under-mattress and smartwatch users had an average sleep duration below 6 hours, respectively. See Supplementary Tables S1–S3 for a comparison of the devices and users’ demographics.

### Ambient temperature and sleep loss

**Figure 2:**
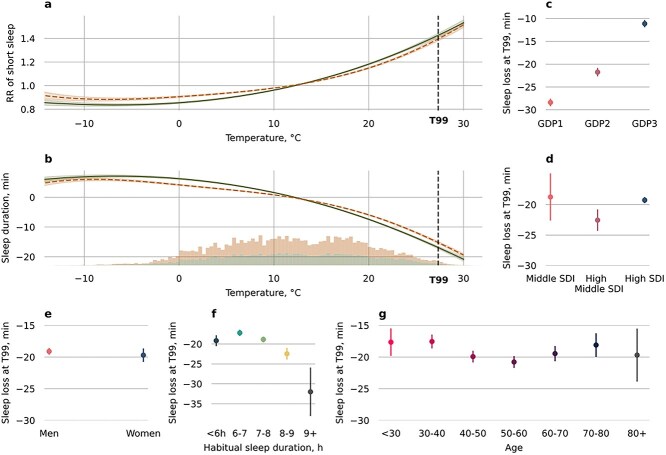
Associations between ambient temperature and sleep for users of the smartwatch (dashed orange line; *n* = 200 879) or under-mattress sensor (solid green line; *n* = 116 789). (a) Risk ratio (95% CI) of short sleep, defined as sleep duration lower than 6 hours. (b) sleep loss (minutes). The vertical dashed line represents the 99th percentile of temperature (T99). Subgroup analyses for users of the under-mattress sensors included sleep loss at T99 based on (c) the country of residence’s gross domestic product (GDP) per capita (tertiles—tertile 1: <USD 46 000 per year; tertile 2: USD 46 000 to USD 69 000 per year; tertile 3: >USD 69 000 per year); (d) the country of residence’s socio-developmental index (SDI); (e) sex; (f) habitual sleep duration; and (g) age. Error bars in (b–g) represent 95% confidence intervals. The number of participants in each subgroup is available in Supplementary Table S4.

Globally, sleep duration was relatively stable for temperatures below 5°C and was negatively associated with temperatures above 5°C. An increase from 12.2°C to 27.3°C, which corresponds to an increase in temperature from the 50th percentile to the 99th percentile globally (T99), was associated with approximately 15 (mean [95%CI]: −15.2 [−15.6, −14.9]) and 17 (−16.9 [−17.4, −16.4]) minutes of sleep loss in the users of the smartwatch and under-mattress sensors, respectively. There was also a 40% (mean [95%CI]: 1.40 [1.38, 1.41]) and 43% (1.43 [1.41, 1.44]) relative increase in the probability of short sleep at T99 for the users of the smartwatch and under-mattress sensors, respectively (Figure 2a). This equated to a 7.73% [7.42%–8.04%] and a 9.34% [8.97%–9.71%] increase in the prevalence of short sleep at T99 for users of the smartwatch and under-mattress sensors, respectively. The effect of temperature on sleep was stronger in countries in the lowest GDP per capita tertiles within the study sample (Figure 2c), whereby T99 (estimated within group rather than globally) was associated with a −11.1 (−11.9, −10.3) minute reduction in total sleep time in the highest GDP per capita tertile countries within the study sample but with a −28.4 (−29.2, −27.5) minute decrease in countries in the lowest tertiles of GDP per capita within the study sample. The same effect was not observed (Figure 2d) for the SDI. The effect of temperature on sleep loss was consistent for habitual sleep durations lower than 9 hours (Figure 2f), but stronger for habitual sleep durations greater than 9 hours (<1% of the population). The effect of temperature on sleep loss generally became stronger with increasing age (Figure 2g). There were no clear sex-specific associations (Figure 2e). Similar trends in subgroup analysis were observed for users of the smartwatch (Supplementary Figure S5). The mean (95%CI) for sleep duration loss and RR (95% CI) for short sleep are available for each subgroup analyses in supplementary Table S4.

### Country differences in sleep loss due to high temperature

**Figure 3:**
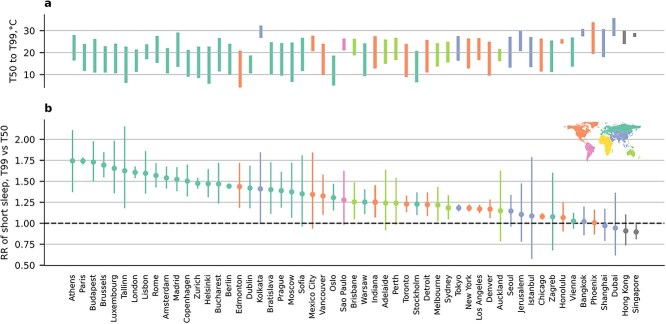
Location by location risk ratio (95% confidence interval) of short sleep (b) at 99th versus 50th percentile of temperature (a) for users of the under-mattress sensor, ranked by effect size estimates. The coloring is based on the continents of residence. See Supplementary Figure S8 for a similar figure with the smartwatch users. See Supplementary Table S5 for the number of users per city.

The magnitude of sleep loss at T99 was dependent on location. For example, greater effect estimates were observed for European cities compared to cities in North America or Australia (Figure 3). Similar results were observed with the smartwatch (Supplementary Figure S6). In some countries, high temperatures (estimated within countries) were associated with an up to 1.75-fold relative increase in the probability of short sleep versus the 50th percentiles (Figure 3). There was some evidence of possible regional adaptation effects. For example, exposure to temperatures of approximately 20°C in some countries (e.g. France and England) were associated with a 60%–75% relative increase in the probability of short sleep, whereas similar temperature exposure in cities within the United States or Australia was associated with a more modest 10%–30% relative increase in the probability of short sleep. In some countries, such as Portugal, a small increase in temperature (T99 vs T50, 23.7 vs 16.9°C) was still associated with a 60% relative increase in the probability of poor sleep (1.60 [1.35, 1.86]). The effect was relatively stable for cities within the United States, with an approximately 10%–20% relative increase in short-sleep probability across the different cities (Supplementary Figure S7). Similar results were found for cities across Australia and Canada (Supplementary Figures S8 and S9). Analyses were repeated with different exposure variables, such as the 24-hour minimum and 24-hour maximum temperature, which yielded similar results (see Supplementary Figure S10). The mean (95%CI) for sleep duration and RR (95% CI) for short-sleep duration of the association with temperature are available for each location in Supplementary Table S5.

### Alternative model specifications and sensitivity analyses

**Figure 4:**
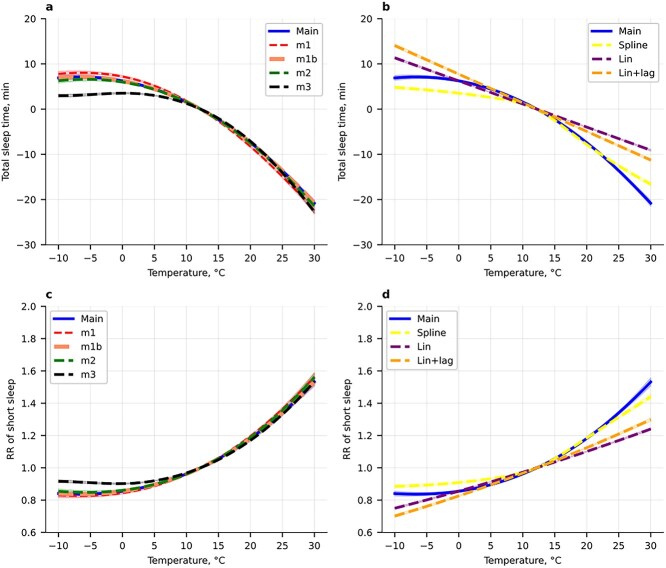
Associations between ambient temperature and sleep duration (a, b) and short sleep (c, d) for users of under-mattress sensors (*n* = 116 789) for different model specifications. M1: daylight duration adjustment; M1b: daylight and day-of-year adjustment; M2: temporal control using a natural cubic spline of time (6 degrees of freedom/year); M3: person–year–month versus person–year–week. M4: exposure of interest $f\left({x}_{it},l\right)$ modeled using splines (4 df) but no lag; M5: linear function; M7: linear function including lagged effect.

Alternative models with different temporal control and/or daylight adjustments did not change the main findings in the global model (Figure 4) or in the country-by-country model (Supplementary Tables S6 and S7). The results were similar for sleep duration (Figure 4a) and short sleep (Figure 4c) as the outcomes and were also similar for users of smartwatches (Supplementary Figure S11). When the exposure of interest was modeled as a spline without lagged effect (Figure 4b and d), the magnitude of the association between high temperature (27.3 vs 12.2°C) and sleep duration and short sleep was slightly smaller (sleep duration: mean [95%CI], −14.5 [−14.8, −14.2] minutes; short sleep: RR [95%CI], 1.37 [1.36, 1.38]) than for the main model (sleep duration: −16.90 [−17.37, −16.44] minutes; short sleep: 1.43 [1.41, 1.44]). The magnitude was even smaller when the exposure of interest was modeled using a linear function without (sleep duration: −7.7 [−7.9, −7.6] minutes; short sleep: 1.20 [1.20, 1.21]) or with a lagged effect (sleep duration: −9.6 [−9.8, −9.3] minutes; short sleep: 1.25 [1.24, 1.26]). Similar results were observed with the country-by-country models, and for users of smartwatches (Supplementary Tables S8 and S9). None of the sensitivity analyses changed the main results (Supplementary Figures S15–S18). Alternative specifications of the spline function to model the exposure–response curve also did not change the main findings (Supplementary Figure S19). Exposure–response curves for the other variables in the model are available in Supplementary Figures S20–S23.

### Heatwaves and sleep loss

**Figure 5:**
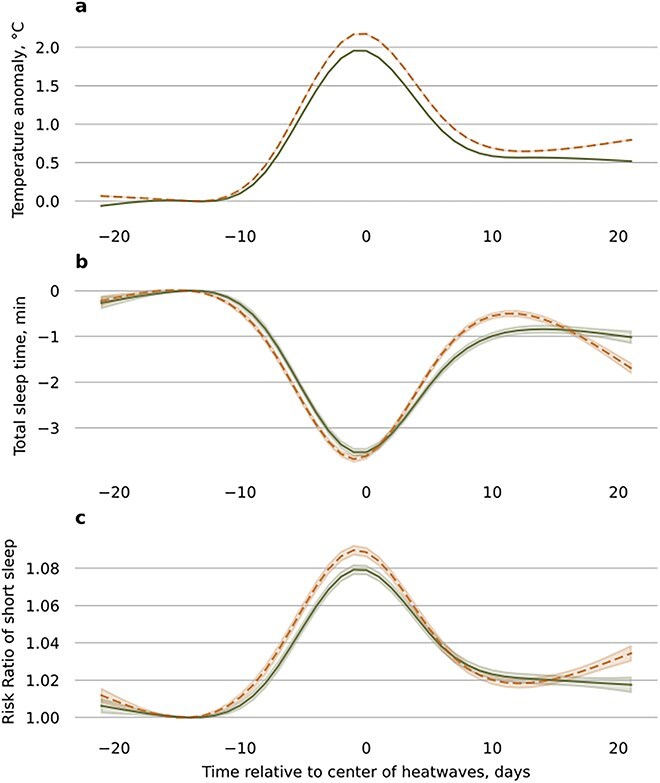
Associations between ambient temperature and sleep surrounding a heatwave event for users of the smartwatch (dashed orange line) or under-mattress sensor (solid green line). (a) Increased temperature profiles during heatwaves, associated with (b) decreased average total sleep time and (c) increased risk of short sleep. The number of participants in each city is available in Supplementary Table S5.

In countries with at least 100 users of each device (Supplementary Table S1), we identified approximately 1300 and 1100 heatwaves across the 3.5 year data-monitoring period for users of the under-mattress sensor and smartwatch devices, respectively. This is equivalent to between 3 and 15 heatwaves per location per year, and similar to published estimates [62]. The median heat wave duration was 6 days, maximum temperature approximately 2.8°C above baseline, and average baseline temperature 17.8°C (Supplementary Figures S12 and S13). On average, heatwaves were associated with an approximately 2°C to 2.5°C increase in 24 hour temperatures compared to baseline and were associated with an approximately 3–4 minute decrease in total sleep time and an 8%–9% relative increase in the probability of short sleep (Figure 5). The magnitude was consistent across the two devices (Figure 5) and across different cities (Supplementary Figure S14). The magnitude of the effect, approximately equal to a 3.5% relative increase in the probability of short sleep per 1°C increase in maximum heatwave temperature, is comparable to the main analysis, where we observed a 40% relative increase in the probability of short sleep for a 15°C in temperature increase.

## Discussion

Ambient temperature changes meaningfully influence sleep duration as quantified objectively in over 300 000 people globally, recorded over an approximately 3 year period. High ambient temperatures (99th vs 50th percentile) are associated with a large (10–40 minute, country-dependent) decrease in sleep duration and a marked relative increase in the probability of short sleep (defined as <6 hours of sleep per night). Many European cities have up to a 75% relative increase in the probability of short sleep during high versus median temperatures. This effect was stronger in older adults and in people living in countries with a lower GDP per capita. The observed effect was also consistent during heatwaves.

Inadequate sleep due to extreme temperatures has been reported in human laboratory studies [23, 24] and real-world environments [25, 26, 63]. However, previous studies were typically cross-sectional, were conducted in a single country, used devices unvalidated with polysomnography, or used self-reported sleep duration. Self-report data from 765 000 US survey respondents from 2002 to 2011, coupled with nighttime temperature data, show that increases in nighttime temperatures increased self-reported nights of insufficient sleep [9]. A study of data from 47 000 users of a wrist-worn consumer device, not yet validated against polysomnography, showed similar but smaller effects [26]. This could contribute to conservative estimates of heat-induced short-sleep prevalence. This previous study also had a large proportion (~30%) of users from Japan [26], a country where average sleep duration is low and the prevalence of short sleep is high [20, 64]. This may have resulted in a ceiling effect in the ability to detect associations between temperature and short sleep/sleep duration. Another recent study of more than 200 000 participants across mainland China found a decrease in sleep duration (measured using an unvalidated smartwatch) with increased ambient temperatures, with a slightly lower magnitude of effect than reported in the current study [56]. Methodological, sample, and measurement differences may explain the apparent differences in effect magnitudes between studies. Indeed, some studies report the linear association between a 1°C or 10°C increase in temperature with sleep duration as their primary outcome, and some studies do not account for lagged effects [25, 56]. Our supplementary analyses suggest that this may lead to an underestimation of the effect. Similar to previous studies, we also observed a greater magnitude of the effect of temperature on sleep loss for older adults and users residing in lower-income countries [26]. Taken together, these studies and our results indicate that temperature-induced sleep inadequacy represents an additional pathway through which climate change may amplify global inequalities. Overall, consistent results in all these previous studies despite the slightly different methodologies highlight the robustness of the association between temperature and reduced sleep duration.

Our study also has several unique strengths. We replicated our estimates with two different consumer devices (under-mattress vs wrist-worn) and different population demographics, which showed consistent effects. We further replicated our results in a different analytical paradigm, by estimating the effect of heatwaves on sleep loss. Sleep loss due to increased temperatures during a heatwave was of similar magnitude to the main analysis and consistent across the two devices. This provides additional support for a causal relationship between high temperature exposure and sleep loss. Another strength of our study is the wider geographical coverage than that of most previous studies, acknowledging European bias, but also a dataset larger than that of prior work. Most users had more than a year and a half of nightly recordings, substantially greater than any previous study.

There are several reasons to suggest that our estimates are conservative and may underestimate the effect of heat-induced sleep loss. Firstly, lower socioeconomic groups are likely to be underrepresented in the current consumer study sample, given that all the participants purchased and owned a sleep-tracking device, which currently costs between USD 100 and USD 600 depending on the device. Hence, participants were also more likely to have had access to more favorable sleeping environments and heat stress–mitigation strategies such as air conditioning, which are less readily available in lower socioeconomic populations [65]. Secondly, the under-mattress sleep sensor and smartwatch used in this study typically overestimate sleep by approximately 30 minutes [30], which is comparable to other validated wearable and nonwearable sleep trackers [31, 33].

There are additional limitations of our study. We did not have access to more precise geographical locations of users beyond the nearest largest city within a country and time zone due to privacy concerns, which may have increased noise in the estimated association between temperature and short sleep. Noisy estimates may have been particularly salient in countries and regions with large landmasses (e.g. the United States) and/or diverse topographies, and could explain some of the difference in observed effect size between countries. Since we did not have the participants’ precise locations, we were unable to control for potential spatial autocorrelation, which may have resulted in inflated risk of type I errors. This limitation is common in studies using wearables and/or smartphones to locate and collect user data due to reidentification and privacy. The devices we used in this study did not record temperature. Hence, we relied on outdoor ambient temperature from reanalysis models, similar to previous studies. Together, these limitations are likely to result in an attenuation of the effects rather than confounding our results [66–68]. It is also possible that the lack of precise geographical locations and the resulting residual measurement error of daylight could explain some of the observed results. However, this is not likely given that the person–year–week intercept should account for average daylight variation in sleep duration within individuals, and further sensitivity analysis controlling for daylight duration did not change the main findings. The data were collected partly during the COVID-19 pandemic period, although this is unlikely to have impacted our results given that sensitivity analyses with data restricted to post-summer 2022 showed similar results. Furthermore, despite the large sample size, some countries had a smaller number of users, and results for these countries should therefore be replicated. Additionally, the device is not validated to detect naps, and we were unable to study whether participants use napping as a strategy to balance nighttime sleep loss. While demographic data are consistent across most countries in our sample, there may have been unobserved demographic differences between the users of each country (socioeconomic status, rates of medical conditions, etc.) that may explain, at least in part, some of the observed differences in the exposure–response curves between ambient temperature and short sleep and sleep duration. Of note, a previous study showed that the demographics (age and BMI) of this user sample were similar to population estimates, but biased in terms of female representation (30% female) [28]. We also had almost no data from lower- and middle-income countries, which is a limitation that applies to most studies in sleep research [15]. Indeed, we only had 1.4% of users residing in countries with a “middle” SDI, and no users residing in countries with a low SDI. Several lower- and middle-income countries are predicted to experience the greatest increases in heat with climate change, and may be the least likely to have the infrastructure to mitigate extreme heat [65, 69].

The observed increase in short-sleep prevalence with high temperature is likely to carry significant population-level societal costs, given the high prevalence and wide-ranging health and safety implications of disrupted and/or short sleep [16–19]. High temperatures are also associated with a large increase in Obstructive sleep apnoea (OSA) severity and prevalence on the same night [70, 71]. Similarly, patients are less likely to use their OSA treatment on nights with a high temperature [72]. Taken together, these studies suggest that sleep inadequacy from high and rising temperatures is likely to be a major threat to human health.

In conclusion, we found that high ambient temperatures are strongly associated with a significant decrease in sleep duration globally. Our findings highlight the urgent need for targeted strategies to mitigate temperature-induced sleep loss and further contribute to knowledge about the threat of climate change–related warming to human health and well-being.

## Code availability

Statistical model specifications are available on GitHub: https://github.com/bastienlechat/OSA_climate.

## Supplementary Material

SUPPLEMENT_zsaf323

## Data Availability

The dataset associated with this study is stored in a proprietary repository (Withings) and cannot be shared publicly due to privacy, ethical, and legal concerns. The investigator team accessed the data through an application process to Withings, and a formal data sharing agreement designed to safeguard user confidentiality, as outlined in the terms and conditions and privacy policy documentation. Queries for data access can be directed to Withings (data_compliance@withings.com) with a timeframe for response of 4 weeks. Specific deidentified raw data that support the findings of this study, including individual data, are available from the corresponding author (bastien.lechat@flinders.edu.au) upon request subject to ethical and data custodian (Withings) approval described above. The timeframe for response to requests will be up to 4 weeks. ERA5 weather data and climate model projections are freely available from the Copernicus data store (https://cds.climate.copernicus.eu/). Information on country GDP is freely available from the World Bank (https://www.worldbank.org/ext/en/home). Exposure–response curves between temperature and sleep duration loss and short sleep derived in this study are available on GitHub: https://github.com/bastienlechat/OSA_climate.
