# Supplementary material for: How do we sleep while our beds are burning? High ambient temperatures are associated with substantial sleep loss
Source: Sleep. 2025 Oct 13;49(7):zsaf323. doi: 10.1093/sleep/zsaf323 (PMC13357511; doi:10.1093/sleep/zsaf323)
Supplement: SUPPLEMENT_zsaf323 [file supplement_zsaf323.docx]

**Supplementary material: How do we sleep while our beds are burning? High ambient temperatures are associated with substantial sleep loss.**

Bastien Lechat^1*#^, Barbara Toson^1*^, Hannah Scott^1^, Duc Phuc Nguyen^1^, Billingsley Kaambwa^2^, Amy C Reynolds^1^, Jack Manners^1^, Robert J. Adams^1^, Jean-Louis Pepin^3^, Sebastien Bailly^3^, Andrew J K Phillips^1^, Pierre Escourrou^4^, Peter Catcheside^1^, Danny J Eckert^1^

* Corresponding author

[bastien.lechat@flinders.edu.au](mailto:bastien.lechat@flinders.edu.au)

Mark Oliphant Building, Level 2, Building A, 5 Laffer Drive, Bedford Park 5042

^1^ Adelaide Institute for Sleep Health and FHMRI Sleep Health, College of Medicine and Public Health, Flinders University, Adelaide, Australia

^2^ Health Economics Unit, College of Medicine and Public Health, Flinders University, Health Sciences Building, Sturt Road, Bedford Park, SA, 5042, Australia

^3^ Univ. Grenoble Alpes, HP2 Laboratory, Inserm U-1300, CHU Grenoble Alpes, 38043 Grenoble, France.

^4^ Centre Interdisciplinaire du Sommeil, Paris, France

**Contents**

[Supplementary Methods 5](#_Toc212017516)

[Statistical analysis 5](#_Toc212017517)

[Supplementary Results 7](#_Toc212017518)

[Participants 7](#_Toc212017519)

[Device comparison 12](#_Toc212017520)

[Ambient temperature and sleep loss 13](#_Toc212017521)

[Heatwaves and sleep loss 35](#_Toc212017522)

[Sensitivity analyses 37](#_Toc212017523)

***List of tables***

[**Table S1:** Users demographics across different country for users with an under-mattress sensor and/or a smartwatch. 9](#_Toc212017528)

[**Table S2:** Comparison of user demographics and sleep duration across both devices. 12](#_Toc212017529)

[**Table S3**: Comparison of user demographics and sleep duration of users with both devices. 13](#_Toc212017530)

[**Table S4:** Associations between high temperature (27°C vs 12.2°C, 99th vs 50^th,^ of the global distribution) and short sleep and sleep duration loss –for each subgroup analyses and both devices. 18](#_Toc212017531)

[**Table S5:** Associations between high temperature and short sleep – RR (95%CI) – and sleep duration loss – Mean (95%CI) - locations by locations. 19](#_Toc212017532)

[**Table S6**: Associations between high temperature (99th vs 50^th,^ of the global distribution) and sleep duration loss - mean (95%CI) - in the users of the under-mattress sensors for each locations based on alternative mode specifications. 22](#_Toc212017533)

[**Table S7:** Associations between high temperature (99th vs 50^th,^ of the global distribution) and short sleep - RR (95%CI) - in the users of the under-mattress sensors for each location based on alternative mode specifications. 25](#_Toc212017534)

[**Table S8:** Associations between high temperature (99th vs 50^th,^ of the global distribution) and sleep duration loss - mean (95%CI) - in the users of the smartwatch sensors for each locations based on alternative mode specifications. 28](#_Toc212017535)

[**Table S9:** Associations between high temperature (99th vs 50^th,^ of the global distribution) and short sleep - RR (95%CI) - in the users of the smartwatch for each location based on alternative mode specifications. 31](#_Toc212017536)

[**Table S10:** Number of users and recordings for different inclusion/exclusion criteria. 37](#_Toc212017537)

***List of figures***

[**Figure S1:** Lagged effect of exposure to temperature on sleep duration. Lag 0 represents the acute effect of exposure, lag 1, 2 and 3 represents the effects on the following 1,2 and 3rd day. Subsample of the dataset (N=10,000) with the under-the-mattress sensors 5](#_Toc212017538)

[**Figure S2:** Cumulative exposure-response curve between temperature and odds of poor sleep for different lag structures (4, 7 and 14 days). Subsample of the dataset (N=10,000) with the under-the-mattress sensors. Note that for 7 and 14 days we used participant-year-month intercept rather than participant-year-week intercept. 6](#_Toc212017539)

[**Figure S3:** Flow chart of inclusion/exclusion criteria for the under-mattress sensor users. 7](#_Toc212017540)

[**Figure S4:** Flow chart of inclusion/exclusion criteria for the smartwatch users. 8](#_Toc212017541)

[**Figure S5:** Associations between ambient temperature and sleep for users using a smartwatch (green) or an under-mattress sensor (green). **a)** Risk ratio (95%CI) of short sleep defined as sleep duration < 6h. **b)** Sleep loss (min). Dashed line represents the 99th percentile of temperature (T99). Subgroup analyses for users of the smartwatch sensors included sleep loss at T99 based on **c)** the country of residence gross domestic product per capita (tertiles); **d)** the country of residence socio-developmental index (SDI) e) sex, f) Habitual sleep duration and **g)** age. Number of participants in each subgroup is available in Table S4. 13](#_Toc212017542)

[**Figure S6:** Location by location Risk ratio (95% confidence interval) of short sleep (bottom) at 99th versus 50th percentile of temperature (top) for users of the smartwatch, ranked by effect size. Coloring is based on the continents of residence. The number of participants in each city is available in Table S5. 14](#_Toc212017543)

[**Figure S7:** Risk ratios (95% confidence interval) of short sleep for different percentile of temperature (50^th^ as the reference) for different locations in the USA for users of the under-mattress sensor (green) and smartwatch (orange). The number of participants in each city is available in Table S5. 14](#_Toc212017544)

[**Figure S8:** Risk ratio (95% confidence interval) of short sleep for different percentile of temperature (50^th^ as the reference) for different locations in Australia for users of the under-mattress sensor (green) and smartwatch (orange). The number of participants in each city is available in Table S5. 15](#_Toc212017545)

[**Figure S9:** Risk ratio (95% confidence interval) of short sleep for different percentile of temperature (50^th^ as the reference) for different locations in Canada for users of the under-mattress sensor (green) and smartwatch (orange). The number of participants in each city is available in Table S5. 16](#_Toc212017546)

[**Figure S10**: Associations between ambient 24h average (green), max (orange) and min (purple) temperature and risk ratio of short sleep (sleep duration <6h) for the top 12 locations with the most under-the-mattress users. Temperature variable was rescaled to percentage based on the maximum and minimum values observed. The number of participants in each city is available in Table S5. 17](#_Toc212017547)

[***Figure S11:*** *Associations between ambient temperature and sleep duration (****a*** *and* ***b****) and short sleep (****c****,* ***d****) for users of smartwatch for different model specifications. M1: daylight duration adjustment; M1b daylight and day of year adjustement; M2 temporal control using a natural cubic spline of time (six degrees of freedom/year); M3: person-year-month vs. person-year-week. M4: exposure of interest* $fxit, l$ *modelled using splines (4df) but no lag; M5: linear function; M7: linear function including lagged effect.* 34](#_Toc212017548)

[**Figure S12:** Heatwaves characteristics (strength, baseline temperature and duration) for the users of the under-mattress sensors. 35](#_Toc212017549)

[**Figure S13:** Heatwaves characteristics (strength, baseline temperature and duration) for the users of the smartwatch. 35](#_Toc212017550)

[**Figure S14:** Increased temperature profiles during heatwaves (blue) and associated increased risk ratio of short sleep for under-mattress users (green) and smartwatch users (orange) for top 12 locations with most users and available heatwaves information. The number of participants in each city is available in Table S5. 36](#_Toc212017551)

[**Figure S15:** Risk ratio (95%CI) of short sleep, defined as sleep duration <6h for different ambient temperature for users using a smartwatch (right) or an under-mattress sensor (left). Different colors represent different cut-offs for the minimum number of weeks to be included in the analysis. 37](#_Toc212017552)

[**Figure S16:** Risk ratio (95%CI) of short sleep, defined as sleep duration <6h for different ambient temperature for users using a smartwatch (right) or an under-mattress sensor (left). The analysis was run on the full dataset (blue) vs nights with sleep duration between 4 and 12h (orange – dashed lines). 38](#_Toc212017553)

[**Figure S17:** Risk ratio (95%CI) of short sleep, defined as sleep duration <6h for different ambient temperature for users using a smartwatch (right) or an under-mattress sensor (left). The analysis was run on the full dataset (blue) vs nights recorded after September (orange – dashed lines). 39](#_Toc212017554)

[**Figure S18**: Risk ratio (95%CI) of short sleep, defined as sleep duration <6h, for different ambient temperature in the fully adjusted models (blue) and minimally adjusted models (black) for the top 12 countries with the most users. Different colors represent different cut-offs for the minimum number of weeks to be included in the analysis. The number of participants in each city is available in Table S5. 40](#_Toc212017555)

[**Figure S19:** Associations between ambient temperature and sleep duration (a and b) and short sleep (c, d) for users of under-mattress sensors (left) and smartwatch (right) for different model specifications. The exposure of interest $fxit, l$ was modelled using different degrees of freedom for the splines including 3df (red), 4df (main model – blue), 6 df and 8 df. 41](#_Toc212017556)

[**Figure S20:** Exposure-response curve for the adjustment variables for the model investigating the association between temperature and sleep duration in users of the under-mattress sensors. Adjustment variables included relative humidity (4 df), total cloud cover (4 df), surface pressure (4 df), average daily density particulate matter with aerodynamic diameter <2.5 µg/m^3^ (4 df), splines of time (day of year variable; with 4 degrees of freedom [df]), daylight duration (4df – sensitivity model only) and total precipitation (4 df). 42](#_Toc212017557)

[**Figure S21**: Exposure-response curve for the adjustment variables for the model investigating the association between temperature and short sleep (<6hr) in users of the under-mattress sensors. Adjustment variables included relative humidity (4 df), total cloud cover (4 df), surface pressure (4 df), average daily density particulate matter with aerodynamic diameter <2.5 µg/m^3^ (4 df), splines of time (day of year variable; with 4 degrees of freedom [df]), daylight duration (4df – sensitivity model only) and total precipitation (4 df). 43](#_Toc212017558)

[**Figure S22:** Exposure-response curve for the adjustment variables for the model investigating the association between temperature and sleep duration in users of the smartwatch. Adjustment variables included relative humidity (4 df), total cloud cover (4 df), surface pressure (4 df), average daily density particulate matter with aerodynamic diameter <2.5 µg/m^3^ (4 df), splines of time (day of year variable; with 4 degrees of freedom [df]), daylight duration (4df – sensitivity model only) and total precipitation (4 df). 44](#_Toc212017559)

[**Figure S23:** Exposure-response curve for the adjustment variables for the model investigating the association between temperature and short sleep (<6hr) in users of the smartwatch. Adjustment variables included relative humidity (4 df), total cloud cover (4 df), surface pressure (4 df), average daily density particulate matter with aerodynamic diameter <2.5 µg/m^3^ (4 df), splines of time (day of year variable; with 4 degrees of freedom [df]), daylight duration (4df – sensitivity model only) and total precipitation (4 df). 45](#_Toc212017560)

# Supplementary Methods

## Statistical analysis

Lagged models were chosen as they can describe complex non-linear exposure-response association at different lags, such that any potential delayed effects (e.g., how temperature on a given day affects sleep two days later) can be assessed. The lag period chosen for this study was 4 days since we observed significant delayed effect of high temperature exposure on sleep time up to day 4 (lag 3; Figure S1). We also tested longer lag structure (up to 14-days) but the cumulative exposure-response curve was similar in all the different lag structures (Figure S2).


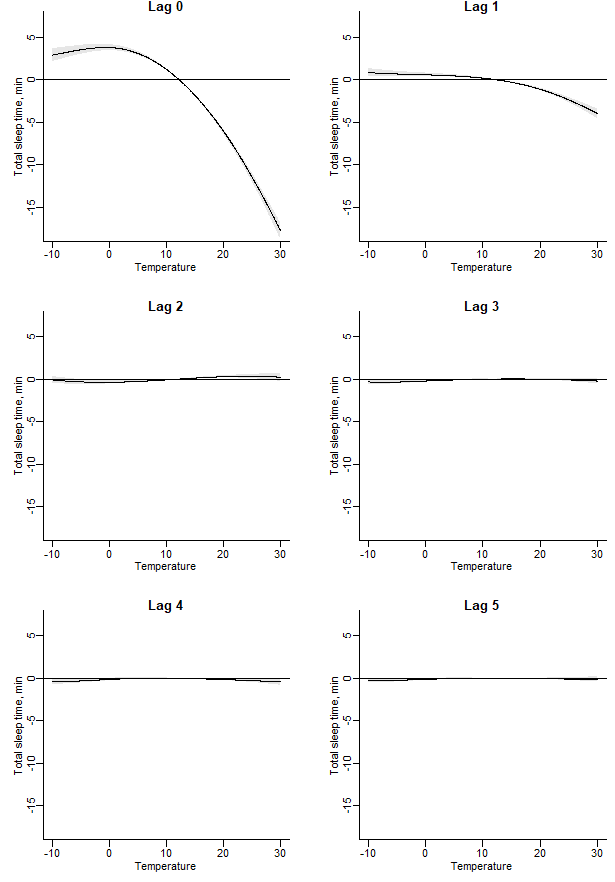


**Figure S1:** Lagged effect of exposure to temperature on sleep duration. Lag 0 represents the acute effect of exposure, lag 1, 2 and 3 represents the effects on the following 1,2 and 3rd day. Subsample of the dataset (N=10,000) with the under-the-mattress sensors


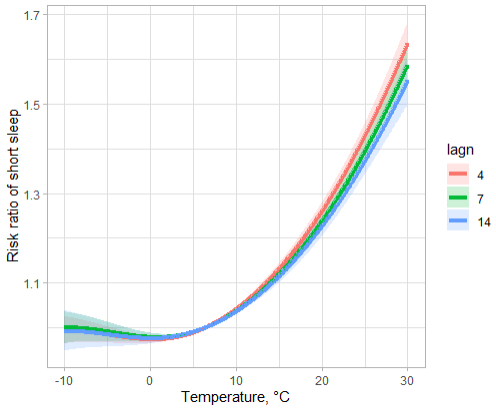


**Figure S2:** Cumulative exposure-response curve between temperature and odds of poor sleep for different lag structures (4, 7 and 14 days). Subsample of the dataset (N=10,000) with the under-the-mattress sensors. Note that for 7 and 14 days we used participant-year-month intercept rather than participant-year-week intercept.

# Supplementary Results

## Participants

There was 125,555 users of the under-mattress sensors and 238,589 users of the smartwatch that used their devices between 2017 and September 2023. In instances where there were multiple sleep episodes within 24h, we assumed that the longest episodes were the main sleep period and discarded the other recordings (~5.7% of recordings). Data before January 2020 was removed given that the under-mattress device was validated in 2020^1^. This resulted in ~6% of the dataset removed. Further inclusion criteria were at least 28 nights of data and an average use of the device of at least 4 times per week, as done in a previous study^2^.

125,555 pxs

81,953,785 nights

Before 2020

260 pxs

6,885,722 nights

125,295 pxs

75,068,063 nights

- < 28 nights **and/or**

- use < 4 times per/week.

8,261 pxs

1,936,792 nights

117,034 pxs

73,137,271 nights

- Missing country and/or weather information

155 pxs

43,154 nights

116,879 pxs

73,094,117 nights

**Figure S3:** Flow chart of inclusion/exclusion criteria for the under-mattress sensor users.

238,589 pxs

96,464,811 nights

Before 2020

39 pxs

6,885,722 nights

238,550 pxs

96,463,874 nights

- < 28 nights **and/or**
- use < 4 times per/week.

37,629 pxs

4,897,157 nights

200,921 pxs

91,566,717 nights

200,879 pxs

91,531,963 nights

- Missing country and/or weather information

42 pxs

34,754 nights

**Figure S4:** Flow chart of inclusion/exclusion criteria for the smartwatch users.

**Table S1:** Users demographics across different country for users with an under-mattress sensor and/or a smartwatch.

|  | **Under-mattress users** | | | | | | **Smartwatch users** | | | | | |
| --- | --- | --- | --- | --- | --- | --- | --- | --- | --- | --- | --- | --- |
| Country | **N, users** | **Nights per users** | **Age,**  **Mean (SD)** | **% Man** | **BMI**  **Mean (SD)** | **TST***  **Mean (SD)** | **N, users** | **Nights per users** | **Age,**  **Mean (SD)** | **% Man** | **BMI**  **Mean (SD)** | **TST***  **Mean (SD)** |
| Australia | 3194 | 403 | 45.5 (13.8) | 77.3 | 28.7  (6.5) | 7.2 (1.1) | 5128 | 411 | 52.7 (15.9) | 66.4 | 28.0 (5.8) | 7.7 (0.9) |
| Austria | 1351 | 668 | 49.1 (13.3) | 80.5 | 27.6 (5.0) | 7.0 (0.9) | 4320 | 427 | 52.3 (15.7) | 64.1 | 26.7 (4.9) | 7.5 (0.8) |
| Belgium | 1624 | 665 | 48.4 (12.5) | 78.9 | 27.0 (4.9) | 7.2 (0.9) | 3237 | 463 | 51.1 (15.0) | 67.6 | 26.7 (4.9) | 7.6 (0.8) |
| Brazil | 188 | 560 | 46.8 (12.4) | 81.4 | 27.6 (4.6) | 7.2 (0.8) | 156 | 357 | 50.4 (14.4) | 83.3 | 26.5 (5.1) | 7.3 (0.8) |
| Bulgaria | 112 | 606 | 47.1 (10.5) | 77.7 | 27.5 (5.6) | 7.1 (0.9) | 289 | 490 | 48.3 (13.1) | 75.8 | 27.1 (4.7) | 7.4 (0.9) |
| Canada | 2932 | 604 | 47.4 (13.6) | 75.2 | 28.0 (6.2) | 7.2 (1.0) | 1812 | 470 | 48.7 (15.2) | 69.4 | 27.1 (5.3) | 7.5 (0.8) |
| China | 297 | 598 | 43.2 (11.8) | 80.1 | 24.8 (3.9) | 6.5 (1.0) | 243 | 402 | 41.3 (11.5) | 79.0 | 24.5 (4.9) | 7.2 (0.8) |
| Croatia | 128 | 627 | 45.9 (12.9) | 83.6 | 27.3 (4.6) | 7.0 (0.8) | 411 | 462 | 48.9 (14.6) | 69.1 | 26.5 (4.5) | 7.4 (0.7) |
| Czech Republic | 504 | 570 | 44.1 (12.1) | 78.2 | 27.6 (5.1) | 7.0 (0.9) | 951 | 433 | 44.6 (14.2) | 75.3 | 26.5 (4.7) | 7.4 (0.7) |
| Denmark | 1071 | 615 | 48.2 (12.5) | 81.2 | 27.7 (5.2) | 7.1 (0.9) | 1477 | 467 | 49.9 (14.8) | 69.5 | 27.0 (4.9) | 7.5 (0.8) |
| Estonia | 156 | 634 | 45.2 (11.7) | 73.1 | 26.7 (5.3) | 7.2 (0.8) | 395 | 522 | 44.1 (12.4) | 57.0 | 27.0 (5.4) | 7.6 (0.7) |
| Finland | 1936 | 626 | 46.8 (13.2) | 79.3 | 28.3 (5.5) | 7.5 (1.2) | 3470 | 483 | 52.7 (15.3) | 66.2 | 27.5 (4.9) | 7.7 (0.8) |
| France | 17398 | 613 | 50.4 (14.2) | 75.5 | 26.7 (4.9) | 7.2 (0.9) | 46599 | 495 | 53.0 (15.6) | 65.8 | 26.3 (4.8) | 7.6 (0.8) |
| Germany | 20585 | 678 | 51.9 (12.9) | 78.7 | 28.1 (5.5) | 7.0 (0.9) | 63256 | 442 | 55.5 (14.7) | 62.6 | 27.1 (5.1) | 7.5 (0.8) |
| Greece | 281 | 608 | 48.4 (12.2) | 80.8 | 27.6 (5.5) | 6.9 (0.9) | 768 | 461 | 51.9 (14.1) | 71.6 | 26.6 (4.6) | 7.2 (0.8) |
| Hungary | 436 | 650 | 46.2 (11.5) | 81.0 | 28.0 (5.3) | 7.0 (0.9) | 1158 | 504 | 48.7 (13.9) | 71.3 | 26.9 (5.0) | 7.4 (0.7) |
| Iceland | na | na | na | na | na | na | 327 | 378 | 48.3 (17.0) | 55.4 | 31.6 (11.5) | 7.7 (0.9) |
| India | 104 | 467 | 48.9 (15.7) | 79.8 | 26.5 (4.5) | 6.7 (1.0) | 249 | 342 | 51.2 (14.6) | 86.3 | 25.5 (4.9) | 6.9 (0.9) |
| Ireland | 405 | 619 | 48.2 (12.8) | 83.2 | 28.3 (6.2) | 7.1 (1.0) | 1429 | 487 | 53.1 (14.5) | 66.6 | 27.7 (5.1) | 7.7 (0.8) |
| Israel | 116 | 665 | 48.5 (13.4) | 80.2 | 27.5 (4.9) | 6.7 (1.1) | 173 | 401 | 49.4 (15.6) | 83.2 | 26.5 (4.5) | 7.1 (0.7) |
| Italy | 1936 | 640 | 50.8 (13.1) | 84.8 | 26.6 (5.2) | 6.8 (0.9) | 3859 | 454 | 54.5 (14.8) | 73.4 | 26.2 (5.7) | 7.3 (0.8) |
| Japan | 4774 | 737 | 46.1 (12.5) | 87.4 | 24.6 (4.3) | 6.4 (1.0) | 2027 | 503 | 48.4 (12.9) | 81.1 | 24.3 (4.2) | 6.9 (0.9) |
| South Korea | 321 | 603 | 41.1 (12.3) | 79.8 | 25.3 (4.3) | 6.4 (1.0) | na | na | na | na | na | na |
| Latvia | na | na | na | na | na | na | 172 | 483 | 45.5 (12.0) | 68.0 | 26.5 (4.7) | 7.5 (0.7) |
| Lithuania | na | na | na | na | na | na | 291 | 470 | 44.8 (13.3) | 58.1 | 25.8 (5.1) | 7.7 (0.8) |
| Luxembourg | 264 | 660 | 50.0 (11.7) | 77.7 | 27.1 (5.0) | 7.1 (0.8) | 456 | 473 | 51.7 (14.4) | 64.3 | 26.9 (4.8) | 7.5 (0.7) |
| Mexico | 281 | 564 | 47.9 (13.3) | 76.9 | 26.3 (4.7) | 7.2 (1.2) | 212 | 380 | 48.2 (14.5) | 83.5 | 27.5 (4.3) | 7.3 (0.7) |
| Morocco | na | na | na | na | na | na | 313 | 394 | 53.9 (16.2) | 73.5 | 26.2 (5.0) | 7.3 (0.9) |
| Netherlands | 2883 | 622 | 47.7 (13.2) | 76.8 | 26.8 (4.9) | 7.3 (0.8) | 3204 | 477 | 50.7 (15.0) | 69.4 | 25.8 (5.2) | 7.6 (0.8) |
| New Zealand | 157 | 573 | 45.8 (12.8) | 79.0 | 27.7 (4.9) | 7.3 (0.9) | 449 | 387 | 49.5 (14.9) | 70.6 | 28.0 (5.5) | 7.7 (0.9) |
| Norway | 979 | 572 | 46.6 (12.8) | 82.0 | 27.2 (5.0) | 7.1 (0.9) | 1868 | 470 | 49.4 (15.1) | 72.3 | 26.7 (4.7) | 7.4 (0.8) |
| Poland | 838 | 549 | 45.8 (12.1) | 80.9 | 27.3 (4.8) | 7.1 (1.0) | 1614 | 453 | 48.3 (14.0) | 72.9 | 26.7 (4.9) | 7.4 (0.8) |
| Portugal | 591 | 616 | 50.6 (13.4) | 76.0 | 26.2 (4.5) | 7.2 (0.9) | 935 | 480 | 52.7 (15.2) | 73.5 | 26.0 (4.2) | 7.5 (0.8) |
| Reunion | na | na | na | na | na | na | 126 | 523 | 49.5 (13.4) | 79.4 | 27.1 (4.8) | 7.3 (0.8) |
| Romania | 415 | 482 | 45.3 (12.5) | 79.3 | 27.5 (5.2) | 7.0 (0.9) | 663 | 416 | 47.2 (13.6) | 70.1 | 26.7 (4.8) | 7.4 (0.8) |
| Russia | 170 | 531 | 45.9 (11.6) | 77.6 | 27.0 (4.9) | 7.0 (0.9) | 345 | 426 | 47.4 (13.8) | 77.7 | 26.5 (4.6) | 7.5 (0.7) |
| Singapore | 305 | 560 | 43.4 (10.7) | 84.6 | 25.9 (4.6) | 6.6 (0.9) | 160 | 442 | 46.7 (12.4) | 73.1 | 25.5 (4.4) | 7.2 (0.8) |
| Slovakia | 189 | 662 | 45.2 (12.3) | 79.9 | 27.2 (5.6) | 7.0 (0.8) | 285 | 478 | 45.5 (14.1) | 69.1 | 26.7 (5.2) | 7.3 (0.7) |
| Slovenia | na | na | na | na | na | na | 173 | 469 | 47.5 (16.0) | 69.4 | 25.8 (4.0) | 7.3 (0.8) |
| South Africa | na | na | na | na | na | na | 114 | 399 | 49.9 (15.6) | 75.4 | 27.8 (5.6) | 7.3 (0.7) |
| Spain | 1572 | 566 | 49.4 (13.3) | 79.9 | 26.9 (4.9) | 7.0 (1.0) | 3182 | 453 | 54.2 (14.3) | 72.2 | 26.6 (4.3) | 7.3 (0.8) |
| Sweden | 1843 | 639 | 48.6 (13.4) | 80.2 | 27.2 (5.3) | 7.3 (0.9) | 3057 | 479 | 50.8 (15.4) | 70.8 | 26.7 (4.7) | 7.5 (0.8) |
| Switzerland | 4556 | 663 | 49.4 (13.0) | 80.0 | 27.0 (4.9) | 7.0 (0.9) | 8274 | 471 | 49.9 (15.0) | 61.1 | 26.3 (4.9) | 7.5 (0.8) |
| Thailand | 221 | 586 | 47.7 (13.7) | 81.0 | 26.0 (5.7) | 6.7 (1.0) | 168 | 416 | 51.0 (15.0) | 80.4 | 26.0 (5.3) | 7.3 (0.8) |
| Tunisia | na | na | na | na | na | na | 106 | 422 | 51.7 (15.8) | 83.0 | 26.6 (4.0) | 7.3 (0.9) |
| Turkey | 106 | 519 | 49.4 (13.2) | 76.4 | 26.9 (4.3) | 7.0 (0.9) | 304 | 404 | 50.1 (14.7) | 73.7 | 27.0 (4.3) | 7.4 (0.9) |
| Ukraine | na | na | na | na | na | na | 122 | 406 | 42.4 (14.0) | 68.0 | 25.7 (5.2) | 7.6 (0.8) |
| UAE | 134 | 527 | 45.1 (9.5) | 85.1 | 27.4 (5.0) | 6.7 (0.9) | 124 | 407 | 44.9 (13.4) | 78.2 | 27.4 (5.4) | 7.2 (0.9) |
| United Kingdom | 7235 | 653 | 48.6 (13.0) | 79.0 | 28.1 (5.9) | 7.2 (1.0) | 13573 | 504 | 52.6 (14.6) | 68.7 | 27.5 (5.5) | 7.6 (0.8) |
| United States | 32038 | 608 | 47.6 (14.1) | 73.2 | 28.4 (6.2) | 7.1 (1.0) | 15955 | 351 | 48.7 (15.0) | 70.3 | 28.2 (6.1) | 7.4 (0.9) |

TST: Total sleep time; BMI: Body Mass Index

## Device comparison

In total, 3,865 users owned both devices at some point during the recording period. Since we used the two devices in separate analysis we considered these users in both analyses. Users with the under-mattress sensors were in general younger than users of the smartwatch. The number of sleep recordings was also higher for users of the under-mattress sensor. Body mass index (BMI) was missing in 92,082 individuals (29%). Average sleep duration was ~25 min lower in the smartwatch users. This is consistent with preliminary validation of the device by the Sleep Revolution consortium (<https://skemman.is/handle/1946/39300>), which suggest an overestimation of total sleep time of ~57 minutes compared to gold standard polysomnography. On the other hand, the under-mattress sensor overestimates sleep duration by ~30 minutes^1,3^, hence the difference of 25 minutes between the two devices is not surprising. 3,865 users (N = 2.9 million nights) owned both devices over the recording period but only 189 users had two devices on the same nights (N = 2,931 nights). In these 3,865 users, sleep duration was also ~21 minutes higher when people used the smartwatch vs the under-mattress sensors, despite similar sleep onset and time out of bed (Table S2). Wake after sleep onset was 13 min lower when people used the smartwatch vs the under-mattress sensors.

**Table S2:** Comparison of user demographics and sleep duration across both devices.

|  |  | Overall | Smartwatch users | Under-mattress  users |
| --- | --- | --- | --- | --- |
| n |  | 317,758 | 200,879 | 116,879 |
| Age, years |  | 51.3 (14.8) | 52.7 (15.2) | 48.8 (13.6) |
| Sex, n (%) | Men | 223,563 (70.4) | 133,230 (66.3) | 90,333 (77.3) |
|  | Women | 94,195 (29.6) | 67,649 (33.7) | 26,546 (22.7) |
| BMI, kg/m^2^ |  | 27.2 (5.4) | 26.9 (5.2) | 27.6 (5.6) |
| Sleep duration, min |  | 440.5 (54.5) | 449.4 (49.5) | 425.1 (59.1) |
| Number of recordings, |  | 518.1 (347.8) | 455.7 (296.8) | 625.4 (398.9) |
| Has smartwatches, n (%) |  | 204744 (64.4) | 200879 (100.0) | 3865 (3.3) |

**Table S3**: Comparison of user demographics and sleep duration of users with both devices.

|  | Smartwatch users | Under-mattress  users |
| --- | --- | --- |
| Sleep duration, min | 446 (51) | 426 (60) |
| Wake after sleep onset, min | 22 (12) | 35 (29) |
| Sleep onset, hh:mm | 23h53 (1h14) | 23h54 (1h22) |
| Start recording, hh:mm | 23h50 (1h14) | 23h25 (1h22) |
| End recording, hh:mm | 7h48 (1h10) | 7h50 (1h20) |
| Number of recordings | 388 (267) | 366 (275) |

## Ambient temperature and sleep loss


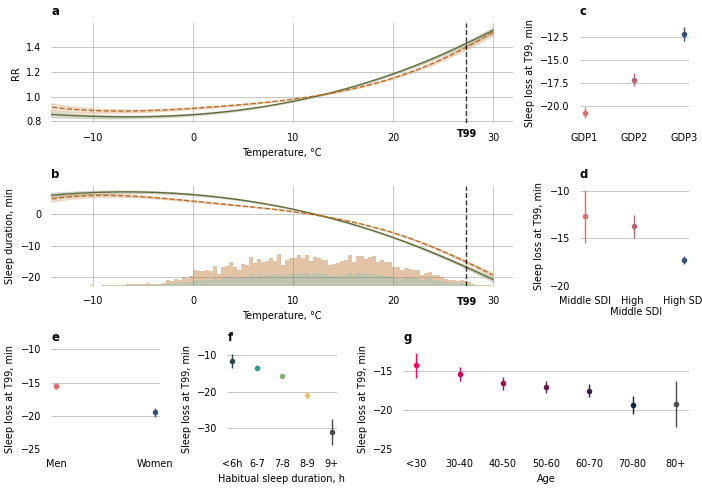


**Figure S5:** Associations between ambient temperature and sleep for users using a smartwatch (green) or an under-mattress sensor (green). **a)** Risk ratio (95%CI) of short sleep defined as sleep duration < 6h. **b)** Sleep loss (min). Dashed line represents the 99th percentile of temperature (T99). Subgroup analyses for users of the smartwatch sensors included sleep loss at T99 based on **c)** the country of residence gross domestic product per capita (tertiles); **d)** the country of residence socio-developmental index (SDI) e) sex, f) Habitual sleep duration and **g)** age. Number of participants in each subgroup is available in Table S4.


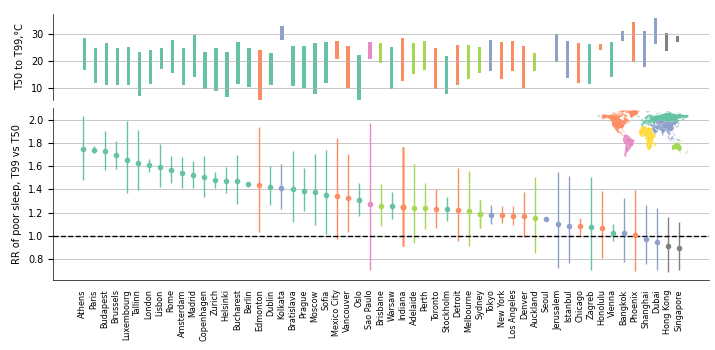


**Figure S6:** Location by location Risk ratio (95% confidence interval) of short sleep (bottom) at 99th versus 50th percentile of temperature (top) for users of the smartwatch, ranked by effect size. Coloring is based on the continents of residence. The number of participants in each city is available in Table S5.

*
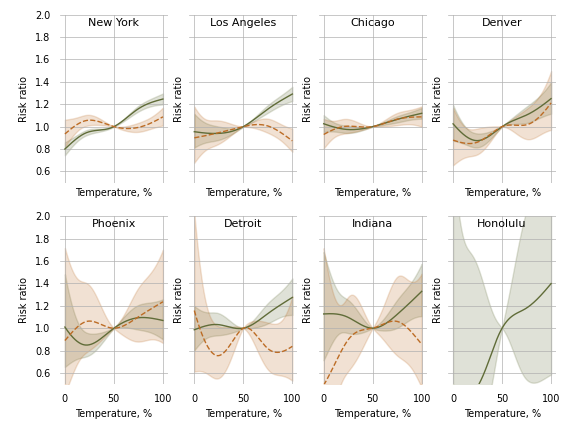
*

**Figure S7:** Risk ratios (95% confidence interval) of short sleep for different percentile of temperature (50^th^ as the reference) for different locations in the USA for users of the under-mattress sensor (green) and smartwatch (orange). The number of participants in each city is available in Table S5.


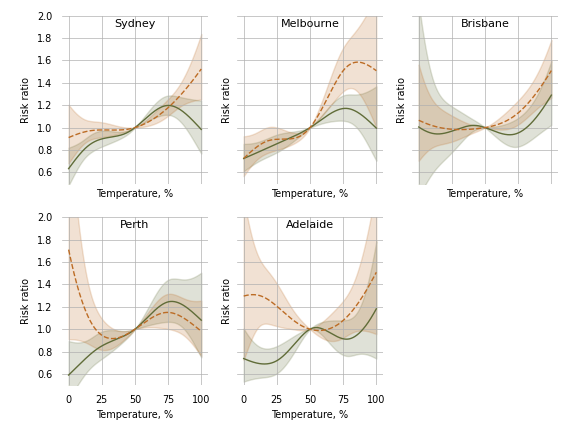


**Figure S8:** Risk ratio (95% confidence interval) of short sleep for different percentile of temperature (50^th^ as the reference) for different locations in Australia for users of the under-mattress sensor (green) and smartwatch (orange). The number of participants in each city is available in Table S5.


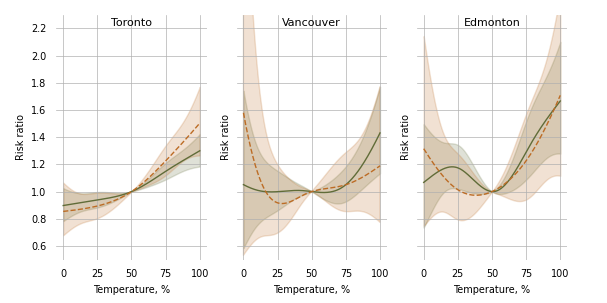


**Figure S9:** Risk ratio (95% confidence interval) of short sleep for different percentile of temperature (50^th^ as the reference) for different locations in Canada for users of the under-mattress sensor (green) and smartwatch (orange). The number of participants in each city is available in Table S5.


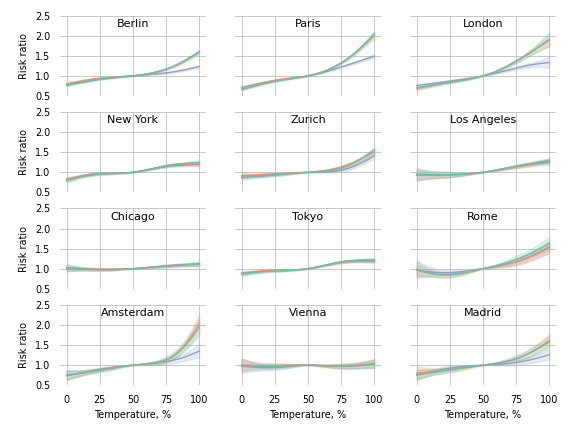


**Figure S10**: Associations between ambient 24h average (green), max (orange) and min (purple) temperature and risk ratio of short sleep (sleep duration <6h) for the top 12 locations with the most under-the-mattress users. Temperature variable was rescaled to percentage based on the maximum and minimum values observed. The number of participants in each city is available in Table S5.

**Table S4:** Associations between high temperature (27°C vs 12.2°C, 99th vs 50^th,^ of the global distribution) and short sleep and sleep duration loss –for each subgroup analyses and both devices.

|  |  | **Number of users** | **Number of users** | **RR (95%CI)**  **Short sleep (6hr sleep)** | | **Mean (95%CI)**  **Sleep duration** | |
| --- | --- | --- | --- | --- | --- | --- | --- |
| Group | **category** | **WSA** | **SCW** | **WSA** | **SCW** | **WSA** | **SCW** |
| overall | overall | 116879 | 200879 | 1.43 (1.41, 1.44) | 1.40 (1.38, 1.41) | -16.9 (-17.4, -16.4) | -15.2 (-15.6, -14.9) |
| Age, years | 0 to 30 | 9401 | 16099 | 1.42 (1.36, 1.49) | 1.32 (1.26, 1.38) | -17.6 (-19.8, -15.5) | -14.2 (-15.8, -12.6) |
|  | 30 to 40 | 25349 | 33319 | 1.41 (1.37, 1.44) | 1.38 (1.34, 1.42) | -17.5 (-18.6, -16.5) | -15.3 (-16.3, -14.4) |
|  | 40 to 50 | 30657 | 36716 | 1.50 (1.47, 1.54) | 1.43 (1.39, 1.47) | -19.9 (-20.8, -19.0) | -16.5 (-17.4, -15.7) |
|  | 50 to 60 | 28300 | 47411 | 1.55 (1.52, 1.59) | 1.47 (1.44, 1.51) | -20.8 (-21.7, -19.8) | -17.1 (-17.8, -16.3) |
|  | 60 to 70 | 15391 | 40951 | 1.57 (1.52, 1.62) | 1.51 (1.47, 1.55) | -19.5 (-20.7, -18.3) | -17.5 (-18.3, -16.7) |
|  | 70 to 80 | 6182 | 21841 | 1.56 (1.48, 1.65) | 1.57 (1.51, 1.62) | -18.1 (-20.0, -16.2) | -19.3 (-20.5, -18.1) |
|  | 80+ | 1599 | 4542 | 1.53 (1.37, 1.71) | 1.57 (1.45, 1.70) | -19.7 (-23.9, -15.5) | -19.2 (-22.1, -16.3) |
| Habitual sleep* | < 6h | 21579 | 13441 | 1.41 (1.37, 1.45) | 1.29 (1.24, 1.35) | -19.2 (-20.5, -17.8) | -11.5 (-13.4, -9.6) |
|  | 6 to 7 h | 52750 | 58149 | 1.46 (1.43, 1.48) | 1.38 (1.36, 1.41) | -17.2 (-18.0, -16.4) | -13.4 (-14.2, -12.6) |
|  | 7 to 8 h | 64827 | 120402 | 1.59 (1.55, 1.62) | 1.48 (1.46, 1.51) | -18.9 (-19.6, -18.1) | -15.7 (-16.2, -15.1) |
|  | 8 to 9 h | 24541 | 63776 | 1.58 (1.49, 1.67) | 1.54 (1.49, 1.60) | -22.5 (-23.9, -21.0) | -21.0 (-21.8, -20.1) |
|  | 9+ h | 5120 | 13179 | 1.49 (1.26, 1.73) | 1.60 (1.43, 1.79) | -32.0 (-38.1, -25.9) | -30.9 (-34.4, -27.4) |
| Sex | Woman | 26546 | 67649 | 1.52 (1.48, 1.57) | 1.51 (1.48, 1.54) | -19.7 (-20.8, -18.6) | -19.5 (-20.2, -18.8) |
|  | Man | 90333 | 133230 | 1.50 (1.48, 1.52) | 1.43 (1.41, 1.45) | -19.1 (-19.6, -18.6) | -15.6 (-16.0, -15.1) |
| GDP | Tertile 1 | 40250 | 68807 | 1.77 (1.74, 1.81) | 1.54 (1.52, 1.57) | -28.4 (-29.2, -27.5) | -20.7 (-21.3, -20.1) |
|  | Tertile 2 | 37802 | 77371 | 1.61 (1.57, 1.65) | 1.48 (1.45, 1.51) | -21.8 (-22.7, -20.8) | -17.1 (-17.8, -16.5) |
|  | Tertile 3 | 38558 | 54405 | 1.25 (1.22, 1.27) | 1.30 (1.27, 1.33) | -11.1 (-11.9, -10.3) | -12.2 (-13.0, -11.5) |
| SDI | High | 107203 | 183160 | 1.50 (1.48, 1.52) | 1.47 (1.45, 1.49) | -19.3 (-19.8, -18.7) | -17.3 (-17.7, -16.9) |
|  | Middle-high | 7156 | 14583 | 1.34 (1.23, 1.47) | 1.25 (1.16, 1.35) | -18.7 (-22.6, -14.9) | -12.7 (-15.5, -9.9) |
|  | Middle | 1819 | 2660 | 1.67 (1.60, 1.74) | 1.39 (1.34, 1.44) | -22.5 (-24.3, -20.8) | -13.7 (-14.9, -12.5) |

**Table S5:** Associations between high temperature and short sleep – RR (95%CI) – and sleep duration loss – Mean (95%CI) - locations by locations.

|  |  |  |  |  | **RR (95%CI)**  **Short sleep (6hr sleep)** | | **Mean (95%CI)**  **Sleep duration** | |
| --- | --- | --- | --- | --- | --- | --- | --- | --- |
| Country | **city** | **N-SCW*** | **N-WSA*** | **Temperature**  **99^th^ vs 50th** | **WSA** | **SCW** | **WSA** | **SCW** |
| Australia | **Sydney** | 3748 | 1177 | 25.0 to 15.8°C | 1.18 (1.05, 1.33) | 1.33 (1.22, 1.46) | -8.8 (-13.3, -4.3) | -13.0 (-15.7, -10.3) |
|  | **Melbourne** | 1980 | 800 | 24.4 to 13.8°C | 1.22 (1.02, 1.43) | 1.74 (1.44, 2.08) | -12.3 (-18.6, -6.0) | -19.0 (-25.1, -12.8) |
|  | **Brisbane** | 1533 | 475 | 26.3 to 18.9°C | 1.26 (1.05, 1.49) | 1.36 (1.19, 1.55) | -3.1 (-9.7, 3.4) | -11.2 (-15.7, -6.7) |
|  | **Perth** | 1064 | 366 | 26.9 to 16.7°C | 1.24 (0.98, 1.54) | 1.04 (0.85, 1.25) | -7.1 (-16.4, 2.1) | -11.0 (-16.5, -5.4) |
|  | **Adelaide** | 803 | 332 | 26.0 to 15.1°C | 1.24 (0.92, 1.64) | 1.22 (0.92, 1.60) | -12.1 (-22.5, -1.7) | -19.3 (-27.4, -11.2) |
| Austria | **Vienna** | 6538 | 1334 | 27.0 to 13.7°C | 1.03 (0.93, 1.12) | 1.07 (1.00, 1.14) | -2.2 (-5.4, 1.1) | -5.3 (-7.4, -3.2) |
| Belgium | **Brussels** | 5484 | 1689 | 22.9 to 11.0°C | 1.69 (1.55, 1.85) | 1.40 (1.28, 1.52) | -21.6 (-24.6, -18.5) | -12.6 (-15.1, -10.1) |
| Brazil | **Sao Paulo** | 319 | 138 | 26.5 to 21.1°C | 1.28 (0.98, 1.63) | 1.71 (1.13, 2.39) | -0.2 (-11.4, 11.1) | -11.5 (-27.7, 4.7) |
| Bulgaria | **Sofia** | 409 | 102 | 26.7 to 11.7°C | 1.35 (0.96, 1.81) | 1.64 (1.31, 2.03) | -23.9 (-36.9, -10.8) | -12.5 (-21.2, -3.9) |
| Canada | **Toronto** | 3211 | 1924 | 24.0 to 9.0°C | 1.23 (1.13, 1.33) | 1.35 (1.19, 1.52) | -8.9 (-11.7, -6.1) | -10.2 (-14.0, -6.3) |
|  | **Vancouver** | 822 | 498 | 24.1 to 9.7°C | 1.33 (1.10, 1.58) | 1.12 (0.83, 1.50) | -10.2 (-16.8, -3.6) | -3.1 (-11.5, 5.3) |
|  | **Edmonton** | 508 | 287 | 21.3 to 4.1°C | 1.44 (1.18, 1.72) | 1.45 (1.05, 1.95) | -10.5 (-17.3, -3.8) | -10.0 (-21.0, 0.9) |
| China | **Shanghai** | 741 | 348 | 30.7 to 18.1°C | 0.97 (0.79, 1.17) | 0.69 (0.47, 0.98) | 0.7 (-7.7, 9.0) | 24.6 (12.2, 36.9) |
| Croatia | **Zagreb** | 477 | 61 | 25.5 to 10.9°C | 1.08 (0.68, 1.60) | 1.51 (1.14, 1.94) | -14.4 (-30.0, 1.3) | -20.1 (-30.1, -10.1) |
| Czechia | **Prague** | 1778 | 516 | 24.3 to 9.5°C | 1.39 (1.19, 1.61) | 1.42 (1.24, 1.62) | -15.9 (-21.5, -10.3) | -10.1 (-14.3, -5.9) |
| Denmark | **Copenhagen** | 2763 | 1042 | 21.3 to 9.0°C | 1.50 (1.32, 1.70) | 1.31 (1.14, 1.49) | -17.2 (-21.5, -12.9) | -7.8 (-11.8, -3.9) |
| Estonia | **Tallinn** | 611 | 163 | 22.8 to 6.3°C | 1.63 (1.18, 2.15) | 1.02 (0.79, 1.30) | -11.0 (-22.2, 0.2) | -9.4 (-16.4, -2.4) |
| Finland | **Helsinki** | 5598 | 1915 | 22.8 to 6.0°C | 1.47 (1.31, 1.65) | 1.20 (1.09, 1.31) | -13.9 (-17.6, -10.2) | -6.7 (-9.3, -4.2) |
| France | **Paris** | 69553 | 17609 | 24.0 to 11.7°C | 1.74 (1.70, 1.78) | 1.53 (1.50, 1.56) | -25.9 (-26.8, -24.9) | -19.9 (-20.5, -19.2) |
| Germany | **Berlin** | 91191 | 21180 | 24.2 to 9.9°C | 1.44 (1.41, 1.47) | 1.30 (1.27, 1.32) | -16.3 (-17.1, -15.6) | -10.9 (-11.4, -10.4) |
| Greece | **Athens** | 1197 | 156 | 28.0 to 16.4°C | 1.74 (1.37, 2.11) | 1.45 (1.19, 1.74) | -26.0 (-38.5, -13.5) | -15.5 (-23.0, -8.0) |
| Hungary | **Budapest** | 1872 | 443 | 26.2 to 10.9°C | 1.73 (1.50, 1.98) | 1.46 (1.31, 1.64) | -24.9 (-30.3, -19.4) | -14.3 (-17.9, -10.7) |
| India | **Kolkata** | 453 | 84 | 32.1 to 27.0°C | 1.41 (1.00, 1.85) | 0.79 (0.61, 1.00) | -14.3 (-30.8, 2.1) | 7.2 (-2.4, 16.9) |
| Ireland | **Dublin** | 1929 | 397 | 18.7 to 10.5°C | 1.42 (1.18, 1.69) | 1.22 (1.06, 1.39) | -19.0 (-25.1, -12.9) | -9.3 (-13.5, -5.2) |
| Israel | **Jerusalem** | 316 | 108 | 30.0 to 20.8°C | 1.11 (0.78, 1.48) | 1.48 (1.09, 1.93) | 0.8 (-15.5, 17.1) | -5.0 (-18.4, 8.4) |
| Italy | **Rome** | 6919 | 1756 | 27.5 to 15.4°C | 1.57 (1.43, 1.72) | 1.40 (1.29, 1.52) | -19.4 (-23.2, -15.7) | -15.1 (-17.8, -12.3) |
| Japan | **Tokyo** | 7211 | 4711 | 27.5 to 16.4°C | 1.18 (1.14, 1.22) | 1.03 (0.95, 1.11) | -10.2 (-12.6, -7.8) | 0.4 (-3.8, 4.6) |
| Luxembourg | **Luxembourg** | 902 | 264 | 24.1 to 10.7°C | 1.66 (1.36, 1.98) | 1.61 (1.32, 1.94) | -23.2 (-30.0, -16.4) | -20.3 (-26.3, -14.3) |
| Mexico | **Mexico_City** | 351 | 176 | 27.7 to 20.7°C | 1.34 (0.94, 1.84) | 1.06 (0.69, 1.56) | -13.8 (-30.2, 2.6) | -5.3 (-20.1, 9.5) |
| Netherlands | **Amsterdam** | 6800 | 2860 | 22.0 to 10.6°C | 1.54 (1.41, 1.67) | 1.35 (1.22, 1.49) | -18.1 (-20.6, -15.7) | -14.7 (-17.3, -12.0) |
| New Zealand | **Auckland** | 680 | 158 | 21.7 to 16.1°C | 1.15 (0.79, 1.63) | 1.48 (1.18, 1.84) | -7.5 (-18.4, 3.4) | -13.4 (-20.3, -6.6) |
| Norway | **Oslo** | 2980 | 987 | 18.7 to 5.1°C | 1.31 (1.15, 1.47) | 1.19 (1.06, 1.34) | -15.8 (-20.7, -11.0) | -8.4 (-12.1, -4.6) |
| Poland | **Warsaw** | 2838 | 835 | 24.3 to 9.3°C | 1.25 (1.11, 1.41) | 1.11 (1.00, 1.23) | -13.8 (-18.3, -9.3) | -5.9 (-9.3, -2.5) |
| Portugal | **Lisbon** | 1754 | 518 | 23.7 to 16.9°C | 1.60 (1.35, 1.86) | 1.22 (1.04, 1.41) | -19.3 (-25.0, -13.6) | -11.0 (-15.7, -6.2) |
| Romania | **Bucharest** | 1320 | 429 | 26.6 to 11.5°C | 1.47 (1.24, 1.72) | 1.28 (1.08, 1.50) | -19.8 (-27.2, -12.5) | -8.2 (-14.0, -2.4) |
| Russia | **Moscow** | 550 | 185 | 24.6 to 6.5°C | 1.37 (1.07, 1.72) | 1.32 (1.04, 1.64) | -15.4 (-25.4, -5.5) | -15.7 (-24.0, -7.3) |
| Singapore | **Singapore** | 520 | 307 | 28.9 to 27.2°C | 0.90 (0.81, 0.99) | 1.03 (0.83, 1.25) | 3.8 (-0.3, 7.9) | 1.4 (-5.6, 8.5) |
| Slovakia | **Bratislava** | 541 | 186 | 24.7 to 10.3°C | 1.40 (1.11, 1.73) | 1.42 (1.13, 1.74) | -13.8 (-21.6, -5.9) | -18.6 (-25.9, -11.3) |
| South Korea | **Seoul** | <100 | 302 | 27.2 to 13.0°C | 1.15 (0.96, 1.34) | na | -9.5 (-20.9, 2.0) | na |
| Spain | **Madrid** | 5658 | 1327 | 29.1 to 13.6°C | 1.52 (1.38, 1.67) | 1.38 (1.27, 1.50) | -16.9 (-21.0, -12.8) | -15.2 (-18.1, -12.3) |
| Sweden | **Stockholm** | 5255 | 1834 | 20.9 to 6.6°C | 1.23 (1.10, 1.37) | 1.07 (0.98, 1.18) | -8.4 (-12.0, -4.8) | 0.1 (-2.6, 2.9) |
| Switzerland | **Zurich** | 13394 | 4631 | 22.7 to 8.4°C | 1.48 (1.41, 1.54) | 1.48 (1.41, 1.55) | -21.7 (-23.3, -20.0) | -17.5 (-19.0, -16.0) |
| Thailand | **Bangkok** | 458 | 194 | 30.7 to 27.4°C | 1.02 (0.86, 1.20) | 0.99 (0.74, 1.29) | -4.8 (-11.7, 2.1) | 5.4 (-3.5, 14.3) |
| Turkey | **Istanbul** | 447 | 58 | 27.1 to 13.4°C | 1.09 (0.58, 1.79) | 0.94 (0.61, 1.36) | -12.8 (-38.5, 13.0) | -19.6 (-34.8, -4.3) |
| United Arab Emirates | **Dubai** | 420 | 132 | 35.7 to 27.8°C | 0.94 (0.62, 1.36) | 0.92 (0.68, 1.21) | -8.2 (-24.7, 8.3) | -2.3 (-13.7, 9.1) |
| United Kingdom | **London** | 22498 | 7210 | 21.9 to 11.3°C | 1.61 (1.54, 1.68) | 1.30 (1.24, 1.35) | -21.4 (-23.0, -19.8) | -11.1 (-12.4, -9.8) |
| United States of America | **New York** | 22222 | 12947 | 26.5 to 12.8°C | 1.18 (1.14, 1.22) | 1.09 (1.02, 1.16) | -9.0 (-10.5, -7.6) | -3.9 (-6.1, -1.7) |
|  | **Los Angeles** | 12293 | 8018 | 26.6 to 16.7°C | 1.17 (1.12, 1.22) | 0.89 (0.80, 0.97) | -9.4 (-10.9, -7.9) | -0.7 (-3.7, 2.4) |
|  | **Chicago** | 11747 | 7496 | 26.4 to 11.5°C | 1.08 (1.04, 1.12) | 1.06 (0.98, 1.13) | -5.8 (-7.2, -4.4) | -6.6 (-9.1, -4.1) |
|  | **Denver** | 2841 | 1686 | 25.0 to 9.1°C | 1.17 (1.06, 1.28) | 1.14 (0.96, 1.35) | -8.4 (-11.8, -4.9) | -1.2 (-6.8, 4.3) |
|  | **Phoenix** | 1085 | 646 | 33.8 to 19.4°C | 1.01 (0.87, 1.16) | 1.16 (0.85, 1.55) | -2.8 (-8.2, 2.6) | -4.5 (-15.2, 6.3) |
|  | **Detroit** | 886 | 718 | 25.6 to 11.0°C | 1.22 (1.09, 1.37) | 0.83 (0.57, 1.19) | -11.0 (-15.0, -7.0) | -10.0 (-20.7, 0.8) |
|  | **Indiana** | 482 | 374 | 27.4 to 12.9°C | 1.25 (1.07, 1.45) | 0.86 (0.52, 1.37) | -10.4 (-16.0, -4.7) | -7.9 (-22.0, 6.2) |
|  | **Honolulu** | <100 | 122 | 26.1 to 24.2°C | 1.07 (0.90, 1.25) | na | 0.6 (-6.2, 7.5) | na |

*Number of users that contributed to the exposure-response curve estimation

**Table S6**: Associations between high temperature (99th vs 50^th,^ of the global distribution) and sleep duration loss - mean (95%CI) - in the users of the under-mattress sensors for each locations based on alternative mode specifications.

| Country | **city** | **Main** | **M1** | **M2** | **M3** | **M4** | **M5** | **M6** |
| --- | --- | --- | --- | --- | --- | --- | --- | --- |
| Australia | **Sydney** | -8.8 (-13.3, -4.3) | -9.7 (-14.2, -5.3) | -8.5 (-13.0, -3.9) | -10.1 (-13.3, -6.9) | -10.2 (-12.6, -7.8) | -9.8 (-11.5, -8.0) | -10.9 (-13.9, -7.9) |
|  | **Melbourne** | -12.3 (-18.6, -6.0) | -13.2 (-19.5, -6.9) | -12.5 (-18.9, -6.2) | -5.7 (-10.1, -1.3) | -13.5 (-16.7, -10.3) | -16.1 (-18.4, -13.8) | -17.6 (-21.4, -13.8) |
|  | **Brisbane** | -3.1 (-9.7, 3.4) | -4.0 (-10.5, 2.5) | -2.8 (-9.3, 3.8) | -1.7 (-6.1, 2.7) | -4.2 (-8.0, -0.4) | -4.4 (-6.7, -2.0) | -2.3 (-6.1, 1.5) |
|  | **Perth** | -7.1 (-16.4, 2.1) | -7.6 (-16.8, 1.5) | -5.0 (-14.4, 4.4) | -11.0 (-18.1, -4.0) | -12.2 (-18.1, -6.3) | -13.9 (-18.0, -9.8) | -10.4 (-16.8, -4.0) |
|  | **Adelaide** | -12.1 (-22.5, -1.7) | -12.8 (-23.2, -2.4) | -12.5 (-22.9, -2.1) | -13.5 (-21.0, -6.0) | -10.7 (-16.4, -5.1) | -9.2 (-13.3, -5.1) | -13.0 (-19.2, -6.9) |
| Austria | **Vienna** | -2.2 (-5.4, 1.1) | -4.5 (-7.7, -1.4) | -3.6 (-6.9, -0.3) | -2.7 (-4.9, -0.4) | -0.9 (-2.9, 1.1) | 0.6 (-0.5, 1.6) | -0.6 (-2.2, 1.0) |
| Belgium | **Brussels** | -21.6 (-24.6, -18.5) | -23.9 (-27.0, -20.9) | -22.2 (-25.3, -19.2) | -21.3 (-23.1, -19.5) | -18.2 (-20.0, -16.4) | -10.0 (-11.2, -8.8) | -11.8 (-13.5, -10.1) |
| Brazil | **Sao Paulo** | -0.2 (-11.4, 11.1) | -1.3 (-12.6, 10.0) | -1.2 (-12.6, 10.1) | -6.7 (-14.0, 0.7) | -6.5 (-14.6, 1.5) | -10.7 (-14.4, -7.0) | -10.0 (-15.2, -4.8) |
| Bulgaria | **Sofia** | -23.9 (-36.9, -10.8) | -25.9 (-38.9, -13.0) | -22.6 (-35.9, -9.2) | -24.0 (-32.3, -15.6) | -28.7 (-39.0, -18.3) | -13.0 (-19.5, -6.5) | -9.5 (-17.7, -1.2) |
| Canada | **Toronto** | -8.9 (-11.7, -6.1) | -10.0 (-12.8, -7.2) | -9.3 (-12.2, -6.3) | -5.6 (-7.5, -3.7) | -10.2 (-12.0, -8.4) | -5.3 (-6.3, -4.2) | -6.4 (-7.9, -4.9) |
|  | **Vancouver** | -10.2 (-16.8, -3.6) | -12.1 (-18.7, -5.5) | -11.1 (-17.8, -4.4) | -14.1 (-18.3, -9.8) | -9.8 (-14.8, -4.8) | -3.4 (-6.5, -0.3) | -2.2 (-6.3, 2.0) |
|  | **Edmonton** | -10.5 (-17.3, -3.8) | -10.2 (-16.9, -3.5) | -10.4 (-17.2, -3.5) | -15.6 (-20.1, -11.2) | -13.3 (-18.0, -8.5) | -2.3 (-4.7, 0.0) | -0.4 (-3.5, 2.6) |
| China | **Shanghai** | 0.7 (-7.7, 9.0) | -1.2 (-9.6, 7.1) | -3.8 (-12.7, 5.1) | -1.2 (-6.3, 3.9) | 1.2 (-5.3, 7.6) | -2.8 (-6.6, 0.9) | -4.7 (-9.5, 0.2) |
| Croatia | **Zagreb** | -14.4 (-30.0, 1.3) | -20.4 (-35.8, -5.0) | -15.9 (-31.9, 0.0) | -26.5 (-36.1, -16.9) | -15.3 (-26.5, -4.0) | -7.6 (-15.0, -0.2) | -6.2 (-16.4, 4.0) |
| Czechia | **Prague** | -15.9 (-21.5, -10.3) | -18.7 (-24.2, -13.2) | -17.1 (-22.7, -11.5) | -22.1 (-25.5, -18.6) | -12.0 (-15.7, -8.3) | -5.3 (-7.7, -2.8) | -8.8 (-12.2, -5.3) |
| Denmark | **Copenhagen** | -17.2 (-21.5, -12.9) | -19.7 (-24.0, -15.4) | -20.0 (-24.4, -15.6) | -17.1 (-19.7, -14.5) | -16.1 (-19.2, -13.0) | -10.1 (-12.2, -8.0) | -9.3 (-12.0, -6.6) |
| Estonia | **Tallinn** | -11.0 (-22.2, 0.2) | -11.3 (-22.5, -0.1) | -11.0 (-22.5, 0.4) | -10.9 (-17.7, -4.1) | -15.7 (-24.6, -6.8) | -4.0 (-9.9, 1.9) | -4.9 (-12.0, 2.1) |
| Finland | **Helsinki** | -13.9 (-17.6, -10.2) | -14.8 (-18.4, -11.1) | -16.0 (-19.8, -12.2) | -14.2 (-16.5, -12.0) | -16.2 (-19.1, -13.4) | -7.6 (-9.5, -5.8) | -6.3 (-8.5, -4.0) |
| France | **Paris** | -25.9 (-26.8, -24.9) | -28.0 (-28.9, -27.0) | -25.8 (-26.7, -24.9) | -25.9 (-26.5, -25.3) | -19.9 (-20.5, -19.2) | -11.6 (-12.0, -11.2) | -15.0 (-15.5, -14.4) |
| Germany | **Berlin** | -16.3 (-17.1, -15.6) | -18.4 (-19.2, -17.7) | -17.5 (-18.3, -16.7) | -21.1 (-21.6, -20.6) | -13.2 (-13.7, -12.7) | -7.2 (-7.6, -6.9) | -9.9 (-10.3, -9.4) |
| Greece | **Athens** | -26.0 (-38.5, -13.5) | -29.2 (-41.5, -16.9) | -23.7 (-36.8, -10.5) | -18.5 (-26.4, -10.6) | -18.6 (-29.4, -7.9) | -7.0 (-13.6, -0.4) | -11.5 (-19.5, -3.5) |
| Hungary | **Budapest** | -24.9 (-30.3, -19.4) | -27.6 (-32.9, -22.2) | -24.7 (-30.3, -19.2) | -18.7 (-22.1, -15.2) | -22.0 (-25.9, -18.2) | -8.6 (-11.2, -6.1) | -12.9 (-16.5, -9.3) |
| India | **Kolkata** | -14.3 (-30.8, 2.1) | -13.6 (-29.9, 2.8) | -14.6 (-31.4, 2.1) | -4.8 (-14.5, 5.0) | -1.6 (-15.7, 12.5) | -5.1 (-11.0, 0.7) | -5.2 (-12.1, 1.7) |
| Ireland | **Dublin** | -19.0 (-25.1, -12.9) | -21.4 (-27.4, -15.3) | -20.6 (-26.8, -14.4) | -15.4 (-19.4, -11.5) | -9.5 (-13.4, -5.7) | -5.7 (-8.4, -3.0) | -9.1 (-12.7, -5.4) |
| Israel | **Jerusalem** | 0.8 (-15.5, 17.1) | 0.6 (-15.4, 16.6) | -4.5 (-21.5, 12.5) | -11.2 (-20.9, -1.4) | 6.8 (-6.4, 20.1) | -8.6 (-16.6, -0.6) | -8.5 (-17.7, 0.7) |
| Italy | **Rome** | -19.4 (-23.2, -15.7) | -22.5 (-26.2, -18.9) | -22.4 (-26.3, -18.6) | -22.1 (-24.2, -20.0) | -18.0 (-21.2, -14.9) | -9.6 (-11.9, -7.3) | -11.1 (-13.8, -8.5) |
| Japan | **Tokyo** | -10.2 (-12.6, -7.8) | -13.2 (-15.5, -10.8) | -14.9 (-17.4, -12.5) | -10.8 (-12.3, -9.4) | -11.1 (-12.9, -9.3) | -7.6 (-8.6, -6.5) | -8.6 (-10.1, -7.1) |
| Luxembourg | **Luxembourg** | -23.2 (-30.0, -16.4) | -25.4 (-32.2, -18.6) | -25.0 (-31.9, -18.2) | -18.1 (-22.3, -13.8) | -16.8 (-21.3, -12.3) | -10.6 (-13.6, -7.7) | -13.8 (-17.8, -9.8) |
| Mexico | **Mexico_City** | -13.8 (-30.2, 2.6) | -15.2 (-31.7, 1.3) | -14.9 (-31.4, 1.6) | -3.1 (-14.1, 7.9) | -12.3 (-24.2, -0.5) | -12.6 (-17.8, -7.5) | -9.6 (-17.4, -1.8) |
| Netherlands | **Amsterdam** | -18.1 (-20.6, -15.7) | -20.6 (-23.0, -18.1) | -19.8 (-22.3, -17.3) | -21.5 (-23.0, -20.1) | -16.9 (-18.4, -15.4) | -10.5 (-11.5, -9.5) | -11.3 (-12.7, -9.8) |
| New Zealand | **Auckland** | -7.5 (-18.4, 3.4) | -9.3 (-20.3, 1.7) | -5.6 (-16.7, 5.5) | -12.4 (-19.2, -5.5) | -10.8 (-18.3, -3.3) | -8.4 (-13.6, -3.1) | -10.5 (-17.2, -3.8) |
| Norway | **Oslo** | -15.8 (-20.7, -11.0) | -18.8 (-23.7, -13.9) | -16.5 (-21.5, -11.5) | -10.5 (-13.5, -7.5) | -17.2 (-20.7, -13.8) | -9.2 (-11.2, -7.1) | -9.0 (-11.6, -6.4) |
| Poland | **Warsaw** | -13.8 (-18.3, -9.3) | -16.0 (-20.5, -11.5) | -15.3 (-19.9, -10.7) | -21.6 (-24.6, -18.6) | -11.6 (-14.9, -8.4) | -6.3 (-8.4, -4.2) | -9.5 (-12.5, -6.6) |
| Portugal | **Lisbon** | -19.3 (-25.0, -13.6) | -19.5 (-25.1, -13.9) | -20.2 (-26.0, -14.4) | -21.9 (-25.3, -18.5) | -15.5 (-19.4, -11.6) | -8.5 (-11.3, -5.8) | -12.8 (-16.4, -9.2) |
| Romania | **Bucharest** | -19.8 (-27.2, -12.5) | -23.1 (-30.4, -15.8) | -20.2 (-27.7, -12.6) | -22.8 (-27.6, -18.0) | -14.3 (-20.0, -8.6) | -6.7 (-10.5, -2.8) | -10.8 (-15.6, -6.0) |
| Russia | **Moscow** | -15.4 (-25.4, -5.5) | -16.4 (-26.2, -6.5) | -15.0 (-25.1, -4.9) | -22.0 (-28.6, -15.4) | -12.9 (-19.7, -6.1) | -6.5 (-10.8, -2.1) | -7.0 (-12.8, -1.2) |
| Singapore | **Singapore** | 3.8 (-0.3, 7.9) | 3.6 (-0.6, 7.9) | 3.0 (-1.1, 7.1) | 3.4 (0.7, 6.1) | 1.4 (-2.7, 5.4) | 0.7 (-0.8, 2.2) | 1.3 (-0.5, 3.2) |
| Slovakia | **Bratislava** | -13.8 (-21.6, -5.9) | -15.1 (-22.8, -7.3) | -15.3 (-23.2, -7.3) | -17.3 (-22.2, -12.5) | -13.7 (-19.2, -8.2) | -6.5 (-10.1, -3.0) | -9.8 (-14.8, -4.8) |
| South Korea | **Seoul** | -9.5 (-20.9, 2.0) | -11.4 (-22.7, -0.1) | -14.5 (-26.4, -2.6) | -3.3 (-9.6, 3.0) | -5.4 (-15.1, 4.3) | -6.7 (-11.5, -1.9) | -9.2 (-15.3, -3.1) |
| Spain | **Madrid** | -16.9 (-21.0, -12.8) | -17.8 (-21.9, -13.7) | -17.3 (-21.5, -13.2) | -23.6 (-26.2, -21.0) | -15.3 (-18.5, -12.2) | -9.9 (-11.8, -7.9) | -12.9 (-15.5, -10.3) |
| Sweden | **Stockholm** | -8.4 (-12.0, -4.8) | -11.6 (-15.1, -8.0) | -9.7 (-13.4, -6.0) | -10.6 (-12.7, -8.5) | -9.8 (-12.6, -7.0) | -6.8 (-8.6, -5.0) | -7.0 (-9.2, -4.8) |
| Switzerland | **Zurich** | -21.7 (-23.3, -20.0) | -22.8 (-24.4, -21.1) | -23.1 (-24.8, -21.5) | -22.1 (-23.1, -21.1) | -16.4 (-17.5, -15.2) | -7.5 (-8.2, -6.7) | -9.9 (-10.9, -8.9) |
| Thailand | **Bangkok** | -4.8 (-11.7, 2.1) | -4.6 (-11.4, 2.3) | -6.1 (-13.0, 0.9) | -3.6 (-8.5, 1.3) | -3.4 (-10.1, 3.3) | -0.3 (-3.2, 2.6) | -2.8 (-6.7, 1.2) |
| Turkey | **Istanbul** | -12.8 (-38.5, 13.0) | -12.4 (-37.9, 13.1) | -17.8 (-44.4, 8.9) | -26.1 (-43.3, -8.8) | -13.2 (-34.5, 8.1) | -8.7 (-21.3, 3.9) | -12.2 (-27.5, 3.1) |
| United Arab Emirates | **Dubai** | -8.2 (-24.7, 8.3) | -9.3 (-25.3, 6.6) | -9.6 (-26.4, 7.3) | 3.8 (-6.6, 14.2) | -2.2 (-16.5, 12.2) | 2.5 (-4.1, 9.1) | 1.5 (-6.2, 9.2) |
| United Kingdom | **London** | -21.4 (-23.0, -19.8) | -23.2 (-24.8, -21.7) | -22.9 (-24.5, -21.3) | -24.1 (-25.1, -23.2) | -21.7 (-22.7, -20.7) | -13.6 (-14.3, -13.0) | -15.0 (-15.9, -14.1) |
| United States of America | **New York** | -9.0 (-10.5, -7.6) | -9.8 (-11.2, -8.4) | -9.8 (-11.3, -8.4) | -5.2 (-6.1, -4.3) | -7.1 (-8.0, -6.2) | -3.8 (-4.3, -3.3) | -5.8 (-6.5, -5.0) |
|  | **Los Angeles** | -9.4 (-10.9, -7.9) | -10.1 (-11.6, -8.5) | -8.5 (-10.1, -7.0) | -6.3 (-7.2, -5.4) | -9.9 (-11.0, -8.8) | -6.7 (-7.4, -6.0) | -6.7 (-7.6, -5.7) |
|  | **Chicago** | -5.8 (-7.2, -4.4) | -6.4 (-7.8, -5.0) | -5.2 (-6.6, -3.8) | -1.7 (-2.7, -0.8) | -3.1 (-4.0, -2.2) | -2.4 (-3.0, -1.9) | -3.0 (-3.8, -2.2) |
|  | **Denver** | -8.4 (-11.8, -4.9) | -9.2 (-12.6, -5.8) | -7.1 (-10.6, -3.6) | -4.8 (-7.1, -2.5) | -7.4 (-9.8, -5.0) | -5.0 (-6.4, -3.6) | -5.4 (-7.3, -3.5) |
|  | **Phoenix** | -2.8 (-8.2, 2.6) | -3.6 (-9.0, 1.7) | -2.4 (-7.9, 3.1) | -3.7 (-7.2, -0.2) | -3.6 (-7.7, 0.5) | -8.0 (-10.4, -5.6) | -8.2 (-11.4, -5.0) |
|  | **Detroit** | -11.0 (-15.0, -7.0) | -12.2 (-16.2, -8.2) | -10.1 (-14.2, -6.1) | -6.6 (-9.3, -3.8) | -9.1 (-11.6, -6.7) | -5.5 (-7.0, -4.0) | -6.1 (-8.3, -3.9) |
|  | **Indiana** | -10.4 (-16.0, -4.7) | -11.2 (-16.7, -5.6) | -11.8 (-17.5, -6.0) | -4.8 (-8.7, -0.8) | -7.0 (-10.5, -3.4) | -3.4 (-5.4, -1.4) | -3.9 (-6.9, -0.9) |
|  | **Honolulu** | 0.6 (-6.2, 7.5) | 1.4 (-5.4, 8.2) | 0.8 (-6.1, 7.8) | -1.6 (-6.4, 3.2) | -7.9 (-15.8, -0.0) | -2.8 (-5.6, -0.1) | -1.6 (-5.4, 2.2) |

*M1: daylight duration adjustment; M2 temporal control using a natural cubic spline of time (six degrees of freedom/year); M3: person-year-month vs. person-year-week. M4: exposure of interest* $f\left( x_{it}, l \right)$ *modelled using splines (4df) but no lag; M5: linear function; M7: linear function including lagged effect.*

**Table S7:** Associations between high temperature (99th vs 50^th,^ of the global distribution) and short sleep - RR (95%CI) - in the users of the under-mattress sensors for each location based on alternative mode specifications.

| Country | **City** | **Main** | **M1** | **M2** | **M3** | **M4** | **M5** | **M6** |
| --- | --- | --- | --- | --- | --- | --- | --- | --- |
| Australia | **Sydney** | 1.18 (1.05, 1.33) | 1.21 (1.07, 1.36) | 1.17 (1.04, 1.32) | 1.18 (1.09, 1.27) | 1.23 (1.15, 1.31) | 1.25 (1.19, 1.31) | 1.26 (1.16, 1.36) |
|  | **Melbourne** | 1.22 (1.02, 1.43) | 1.23 (1.04, 1.45) | 1.22 (1.03, 1.44) | 1.09 (0.98, 1.22) | 1.31 (1.21, 1.42) | 1.41 (1.33, 1.49) | 1.36 (1.23, 1.50) |
|  | **Brisbane** | 1.26 (1.05, 1.49) | 1.30 (1.08, 1.53) | 1.25 (1.04, 1.49) | 1.07 (0.96, 1.20) | 1.22 (1.10, 1.35) | 1.11 (1.04, 1.18) | 1.04 (0.93, 1.16) |
|  | **Perth** | 1.24 (0.98, 1.54) | 1.23 (0.98, 1.53) | 1.19 (0.93, 1.48) | 1.18 (1.01, 1.37) | 1.36 (1.18, 1.56) | 1.35 (1.22, 1.49) | 1.36 (1.16, 1.57) |
|  | **Adelaide** | 1.24 (0.92, 1.64) | 1.25 (0.92, 1.65) | 1.26 (0.93, 1.66) | 1.18 (0.97, 1.42) | 1.14 (0.96, 1.33) | 1.23 (1.09, 1.38) | 1.28 (1.07, 1.52) |
| Austria | **Vienna** | 1.03 (0.93, 1.12) | 1.08 (0.99, 1.18) | 1.07 (0.97, 1.17) | 1.00 (0.94, 1.05) | 1.00 (0.94, 1.05) | 1.00 (0.96, 1.03) | 1.01 (0.97, 1.06) |
| Belgium | **Brussels** | 1.69 (1.55, 1.85) | 1.75 (1.60, 1.91) | 1.74 (1.59, 1.90) | 1.63 (1.55, 1.71) | 1.56 (1.47, 1.65) | 1.34 (1.29, 1.40) | 1.45 (1.37, 1.53) |
| Brazil | **Sao Paulo** | 1.28 (0.98, 1.63) | 1.29 (0.99, 1.64) | 1.30 (1.00, 1.65) | 1.38 (1.19, 1.59) | 1.19 (0.99, 1.42) | 1.18 (1.08, 1.29) | 1.13 (0.99, 1.28) |
| Bulgaria | **Sofia** | 1.35 (0.96, 1.81) | 1.38 (0.99, 1.84) | 1.30 (0.91, 1.76) | 1.40 (1.16, 1.66) | 1.54 (1.20, 1.91) | 1.29 (1.09, 1.51) | 1.23 (0.98, 1.51) |
| Canada | **Toronto** | 1.23 (1.13, 1.33) | 1.25 (1.15, 1.35) | 1.20 (1.11, 1.31) | 1.15 (1.09, 1.20) | 1.27 (1.20, 1.33) | 1.13 (1.09, 1.16) | 1.15 (1.10, 1.20) |
|  | **Vancouver** | 1.33 (1.10, 1.58) | 1.38 (1.15, 1.64) | 1.35 (1.12, 1.61) | 1.38 (1.24, 1.53) | 1.31 (1.14, 1.50) | 1.10 (1.00, 1.21) | 1.07 (0.95, 1.21) |
|  | **Edmonton** | 1.44 (1.18, 1.72) | 1.43 (1.18, 1.71) | 1.43 (1.17, 1.71) | 1.44 (1.29, 1.60) | 1.48 (1.29, 1.67) | 1.09 (1.01, 1.17) | 1.05 (0.96, 1.16) |
| China | **Shanghai** | 0.97 (0.79, 1.17) | 0.98 (0.80, 1.18) | 1.07 (0.87, 1.29) | 1.12 (1.01, 1.23) | 0.94 (0.80, 1.09) | 1.04 (0.95, 1.13) | 1.13 (1.01, 1.26) |
| Croatia | **Zagreb** | 1.08 (0.68, 1.60) | 1.27 (0.82, 1.82) | 1.10 (0.69, 1.64) | 1.48 (1.20, 1.79) | 1.34 (1.00, 1.74) | 1.16 (0.94, 1.41) | 1.03 (0.75, 1.37) |
| Czechia | **Prague** | 1.39 (1.19, 1.61) | 1.45 (1.24, 1.68) | 1.42 (1.21, 1.65) | 1.54 (1.42, 1.66) | 1.31 (1.18, 1.45) | 1.13 (1.04, 1.22) | 1.16 (1.04, 1.28) |
| Denmark | **Copenhagen** | 1.50 (1.32, 1.70) | 1.57 (1.38, 1.77) | 1.60 (1.40, 1.81) | 1.44 (1.34, 1.54) | 1.46 (1.33, 1.60) | 1.35 (1.26, 1.44) | 1.33 (1.22, 1.44) |
| Estonia | **Tallinn** | 1.63 (1.18, 2.15) | 1.65 (1.19, 2.18) | 1.46 (1.03, 1.97) | 1.45 (1.22, 1.72) | 1.58 (1.23, 2.00) | 1.16 (0.96, 1.39) | 1.26 (1.01, 1.56) |
| Finland | **Helsinki** | 1.47 (1.31, 1.65) | 1.51 (1.35, 1.69) | 1.55 (1.38, 1.74) | 1.47 (1.38, 1.56) | 1.53 (1.40, 1.66) | 1.27 (1.19, 1.35) | 1.26 (1.17, 1.36) |
| France | **Paris** | 1.74 (1.70, 1.78) | 1.79 (1.75, 1.84) | 1.75 (1.71, 1.79) | 1.69 (1.67, 1.72) | 1.57 (1.54, 1.60) | 1.35 (1.34, 1.37) | 1.46 (1.44, 1.49) |
| Germany | **Berlin** | 1.44 (1.41, 1.47) | 1.50 (1.47, 1.53) | 1.49 (1.45, 1.52) | 1.52 (1.50, 1.53) | 1.36 (1.34, 1.38) | 1.21 (1.20, 1.22) | 1.28 (1.26, 1.29) |
| Greece | **Athens** | 1.74 (1.37, 2.11) | 1.82 (1.45, 2.17) | 1.71 (1.32, 2.10) | 1.44 (1.23, 1.65) | 1.40 (1.10, 1.72) | 1.30 (1.11, 1.49) | 1.47 (1.23, 1.71) |
| Hungary | **Budapest** | 1.73 (1.50, 1.98) | 1.81 (1.57, 2.06) | 1.72 (1.49, 1.98) | 1.46 (1.34, 1.58) | 1.59 (1.43, 1.76) | 1.21 (1.11, 1.30) | 1.35 (1.21, 1.50) |
| India | **Kolkata** | 1.41 (1.00, 1.85) | 1.41 (1.00, 1.84) | 1.44 (1.02, 1.89) | 1.26 (1.05, 1.48) | 1.25 (0.92, 1.62) | 1.12 (0.98, 1.27) | 1.09 (0.92, 1.27) |
| Ireland | **Dublin** | 1.42 (1.18, 1.69) | 1.49 (1.24, 1.76) | 1.50 (1.25, 1.78) | 1.38 (1.24, 1.53) | 1.10 (0.97, 1.24) | 1.09 (0.99, 1.18) | 1.17 (1.04, 1.31) |
| Israel | **Jerusalem** | 1.11 (0.78, 1.48) | 1.01 (0.71, 1.36) | 1.31 (0.95, 1.71) | 1.20 (1.01, 1.40) | 1.00 (0.75, 1.29) | 1.30 (1.12, 1.49) | 1.31 (1.10, 1.53) |
| Italy | **Rome** | 1.57 (1.43, 1.72) | 1.64 (1.50, 1.79) | 1.65 (1.50, 1.81) | 1.53 (1.46, 1.60) | 1.50 (1.39, 1.63) | 1.25 (1.17, 1.33) | 1.32 (1.23, 1.42) |
| Japan | **Tokyo** | 1.18 (1.14, 1.22) | 1.22 (1.18, 1.26) | 1.25 (1.20, 1.29) | 1.17 (1.15, 1.19) | 1.20 (1.17, 1.23) | 1.12 (1.11, 1.14) | 1.14 (1.12, 1.17) |
| Luxembourg | **Luxembourg** | 1.66 (1.36, 1.98) | 1.70 (1.40, 2.04) | 1.73 (1.42, 2.07) | 1.50 (1.34, 1.67) | 1.43 (1.24, 1.63) | 1.31 (1.19, 1.44) | 1.43 (1.26, 1.62) |
| Mexico | **Mexico_City** | 1.34 (0.94, 1.84) | 1.36 (0.95, 1.87) | 1.35 (0.94, 1.85) | 1.23 (0.99, 1.51) | 1.17 (0.90, 1.50) | 1.15 (1.02, 1.29) | 1.08 (0.90, 1.28) |
| Netherlands | **Amsterdam** | 1.54 (1.41, 1.67) | 1.60 (1.47, 1.73) | 1.60 (1.47, 1.74) | 1.63 (1.56, 1.71) | 1.56 (1.48, 1.64) | 1.36 (1.31, 1.41) | 1.36 (1.29, 1.44) |
| New Zealand | **Auckland** | 1.15 (0.79, 1.63) | 1.23 (0.84, 1.72) | 1.12 (0.76, 1.60) | 1.33 (1.08, 1.61) | 1.25 (0.97, 1.58) | 1.24 (1.03, 1.48) | 1.28 (1.01, 1.59) |
| Norway | **Oslo** | 1.31 (1.15, 1.47) | 1.36 (1.20, 1.52) | 1.35 (1.19, 1.53) | 1.22 (1.13, 1.30) | 1.27 (1.16, 1.38) | 1.16 (1.09, 1.22) | 1.16 (1.08, 1.25) |
| Poland | **Warsaw** | 1.25 (1.11, 1.41) | 1.31 (1.16, 1.46) | 1.31 (1.16, 1.47) | 1.37 (1.28, 1.46) | 1.25 (1.15, 1.36) | 1.16 (1.10, 1.23) | 1.18 (1.09, 1.28) |
| Portugal | **Lisbon** | 1.60 (1.35, 1.86) | 1.61 (1.37, 1.87) | 1.65 (1.40, 1.92) | 1.55 (1.42, 1.68) | 1.38 (1.23, 1.55) | 1.26 (1.15, 1.37) | 1.45 (1.30, 1.61) |
| Romania | **Bucharest** | 1.47 (1.24, 1.72) | 1.56 (1.32, 1.81) | 1.50 (1.26, 1.75) | 1.59 (1.45, 1.74) | 1.42 (1.24, 1.61) | 1.17 (1.06, 1.29) | 1.23 (1.08, 1.38) |
| Russia | **Moscow** | 1.37 (1.07, 1.72) | 1.37 (1.06, 1.72) | 1.41 (1.09, 1.77) | 1.44 (1.25, 1.65) | 1.43 (1.20, 1.67) | 1.23 (1.09, 1.37) | 1.13 (0.96, 1.32) |
| Singapore | **Singapore** | 0.90 (0.81, 0.99) | 0.89 (0.80, 0.98) | 0.90 (0.82, 1.00) | 0.94 (0.88, 0.99) | 1.02 (0.92, 1.12) | 1.00 (0.97, 1.04) | 0.98 (0.94, 1.03) |
| Slovakia | **Bratislava** | 1.40 (1.11, 1.73) | 1.43 (1.14, 1.76) | 1.46 (1.16, 1.80) | 1.39 (1.23, 1.57) | 1.36 (1.16, 1.58) | 1.09 (0.97, 1.22) | 1.18 (1.00, 1.37) |
| South Korea | **Seoul** | 1.15 (0.96, 1.34) | 1.16 (0.98, 1.35) | 1.20 (1.00, 1.40) | 1.03 (0.94, 1.12) | 1.09 (0.93, 1.25) | 1.11 (1.03, 1.19) | 1.16 (1.06, 1.26) |
| Spain | **Madrid** | 1.52 (1.38, 1.67) | 1.54 (1.40, 1.68) | 1.53 (1.39, 1.68) | 1.56 (1.48, 1.65) | 1.42 (1.32, 1.53) | 1.26 (1.19, 1.33) | 1.34 (1.25, 1.43) |
| Sweden | **Stockholm** | 1.23 (1.10, 1.37) | 1.31 (1.17, 1.45) | 1.27 (1.13, 1.42) | 1.31 (1.24, 1.38) | 1.27 (1.17, 1.38) | 1.23 (1.16, 1.30) | 1.26 (1.17, 1.35) |
| Switzerland | **Zurich** | 1.48 (1.41, 1.54) | 1.49 (1.43, 1.56) | 1.52 (1.45, 1.59) | 1.49 (1.45, 1.52) | 1.35 (1.31, 1.39) | 1.17 (1.14, 1.19) | 1.23 (1.20, 1.27) |
| Thailand | **Bangkok** | 1.02 (0.86, 1.20) | 1.01 (0.85, 1.19) | 1.04 (0.87, 1.23) | 1.02 (0.91, 1.13) | 1.05 (0.89, 1.23) | 1.01 (0.93, 1.08) | 1.00 (0.90, 1.11) |
| Turkey | **Istanbul** | 1.09 (0.58, 1.79) | 1.06 (0.56, 1.76) | 1.09 (0.56, 1.82) | 1.51 (1.11, 1.95) | 1.12 (0.68, 1.69) | 1.14 (0.84, 1.49) | 1.10 (0.75, 1.52) |
| United Arab Emirates | **Dubai** | 0.94 (0.62, 1.36) | 0.99 (0.66, 1.41) | 0.97 (0.63, 1.41) | 0.91 (0.72, 1.13) | 0.79 (0.54, 1.11) | 0.89 (0.75, 1.04) | 0.96 (0.80, 1.15) |
| United Kingdom | **London** | 1.61 (1.54, 1.68) | 1.65 (1.58, 1.72) | 1.65 (1.58, 1.72) | 1.61 (1.57, 1.65) | 1.60 (1.56, 1.65) | 1.38 (1.36, 1.41) | 1.40 (1.36, 1.44) |
| United States of America | **New York** | 1.18 (1.14, 1.22) | 1.19 (1.15, 1.23) | 1.20 (1.16, 1.25) | 1.13 (1.11, 1.15) | 1.16 (1.13, 1.19) | 1.10 (1.08, 1.11) | 1.14 (1.12, 1.16) |
|  | **Los Angeles** | 1.17 (1.12, 1.22) | 1.18 (1.13, 1.23) | 1.16 (1.11, 1.21) | 1.16 (1.13, 1.18) | 1.21 (1.17, 1.24) | 1.16 (1.14, 1.19) | 1.15 (1.12, 1.18) |
|  | **Chicago** | 1.08 (1.04, 1.12) | 1.09 (1.05, 1.13) | 1.08 (1.04, 1.12) | 1.04 (1.02, 1.06) | 1.06 (1.03, 1.08) | 1.05 (1.03, 1.06) | 1.06 (1.03, 1.08) |
|  | **Denver** | 1.17 (1.06, 1.28) | 1.18 (1.07, 1.30) | 1.15 (1.04, 1.26) | 1.13 (1.06, 1.19) | 1.17 (1.09, 1.25) | 1.13 (1.09, 1.18) | 1.14 (1.08, 1.20) |
|  | **Phoenix** | 1.01 (0.87, 1.16) | 1.03 (0.89, 1.18) | 1.01 (0.86, 1.16) | 1.05 (0.97, 1.15) | 1.00 (0.90, 1.12) | 1.13 (1.06, 1.20) | 1.13 (1.03, 1.22) |
|  | **Detroit** | 1.22 (1.09, 1.37) | 1.23 (1.09, 1.37) | 1.21 (1.07, 1.36) | 1.14 (1.06, 1.22) | 1.18 (1.10, 1.26) | 1.10 (1.05, 1.15) | 1.10 (1.03, 1.18) |
|  | **Indiana** | 1.25 (1.07, 1.45) | 1.26 (1.08, 1.46) | 1.28 (1.10, 1.49) | 1.13 (1.02, 1.25) | 1.15 (1.04, 1.27) | 1.05 (0.99, 1.12) | 1.07 (0.97, 1.16) |
|  | **Honolulu** | 1.07 (0.90, 1.25) | 1.04 (0.88, 1.22) | 1.09 (0.92, 1.28) | 1.00 (0.90, 1.11) | 1.14 (0.94, 1.35) | 1.09 (1.02, 1.16) | 1.06 (0.97, 1.16) |

*M1: daylight duration adjustment; M2 temporal control using a natural cubic spline of time (six degrees of freedom/year); M3: person-year-month vs. person-year-week. M4: exposure of interest* $f\left( x_{it}, l \right)$ *modelled using splines (4df) but no lag; M5: linear function; M7: linear function including lagged effect.*

**Table S8:** Associations between high temperature (99th vs 50^th,^ of the global distribution) and sleep duration loss - mean (95%CI) - in the users of the smartwatch sensors for each locations based on alternative mode specifications.

| Country | **City** | **Main** | **M1** | **M2** | **M3** | **M4** | **M5** | **M6** |
| --- | --- | --- | --- | --- | --- | --- | --- | --- |
| Australia | **Sydney** | -13.0 (-15.7, -10.3) | -13.6 (-16.3, -10.9) | -12.8 (-15.5, -10.1) | -8.5 (-10.3, -6.7) | -11.4 (-13.0, -9.9) | -7.9 (-9.1, -6.8) | -9.6 (-11.5, -7.8) |
|  | **Melbourne** | -19.0 (-25.1, -12.8) | -19.2 (-25.3, -13.1) | -19.1 (-25.2, -13.0) | -10.6 (-14.2, -6.9) | -13.4 (-17.0, -9.8) | -13.6 (-16.2, -11.1) | -21.5 (-25.5, -17.6) |
|  | **Brisbane** | -11.2 (-15.7, -6.7) | -11.4 (-16.0, -6.9) | -10.2 (-14.7, -5.6) | -12.7 (-15.6, -9.7) | -6.5 (-9.2, -3.8) | -5.7 (-7.4, -4.1) | -6.6 (-9.2, -4.0) |
|  | **Perth** | -11.0 (-16.5, -5.4) | -13.1 (-18.5, -7.6) | -7.9 (-13.5, -2.3) | -6.8 (-10.6, -3.0) | -11.8 (-15.4, -8.3) | -10.9 (-13.4, -8.5) | -11.5 (-15.1, -7.8) |
|  | **Adelaide** | -19.3 (-27.4, -11.2) | -19.9 (-28.0, -11.8) | -21.0 (-29.1, -12.8) | -20.8 (-26.3, -15.3) | -15.7 (-20.9, -10.6) | -9.4 (-13.3, -5.6) | -8.5 (-14.1, -2.9) |
| Austria | **Vienna** | -5.3 (-7.4, -3.2) | -6.7 (-8.7, -4.6) | -7.1 (-9.2, -4.9) | -5.7 (-7.1, -4.2) | -3.0 (-4.3, -1.6) | -0.3 (-1.0, 0.4) | -1.1 (-2.2, -0.1) |
| Belgium | **Brussels** | -12.6 (-15.1, -10.1) | -14.9 (-17.4, -12.4) | -13.6 (-16.1, -11.1) | -7.4 (-8.9, -5.9) | -10.1 (-11.7, -8.4) | -4.7 (-5.8, -3.6) | -8.1 (-9.6, -6.6) |
| Brazil | **Sao Paulo** | -11.5 (-27.7, 4.7) | -12.1 (-28.2, 4.1) | -10.6 (-26.8, 5.7) | -7.0 (-17.3, 3.2) | -5.5 (-16.8, 5.9) | -7.2 (-11.1, -3.3) | -7.9 (-13.2, -2.5) |
| Bulgaria | **Sofia** | -12.5 (-21.2, -3.9) | -14.9 (-23.5, -6.4) | -11.1 (-19.9, -2.3) | -13.1 (-18.6, -7.5) | -10.8 (-17.7, -3.8) | -5.3 (-9.6, -0.9) | -10.9 (-16.4, -5.4) |
| Canada | **Toronto** | -10.2 (-14.0, -6.3) | -10.6 (-14.4, -6.7) | -11.8 (-15.7, -7.8) | -8.9 (-11.4, -6.5) | -5.9 (-8.5, -3.4) | -3.6 (-5.0, -2.1) | -7.6 (-9.5, -5.6) |
|  | **Vancouver** | -3.1 (-11.5, 5.3) | -4.1 (-12.5, 4.4) | -3.1 (-11.7, 5.4) | -8.2 (-13.3, -3.2) | -8.3 (-14.6, -2.0) | -0.2 (-4.1, 3.7) | -1.3 (-6.4, 3.8) |
|  | **Edmonton** | -10.0 (-21.0, 0.9) | -10.9 (-21.8, 0.0) | -8.1 (-19.2, 3.0) | -4.4 (-11.4, 2.6) | -13.8 (-21.5, -6.2) | -2.6 (-6.3, 1.1) | -4.1 (-8.8, 0.5) |
| China | **Shanghai** | 24.6 (12.2, 36.9) | 18.7 (6.4, 31.0) | 11.8 (-1.3, 24.9) | 15.9 (8.1, 23.6) | 15.7 (5.6, 25.8) | 5.7 (-0.3, 11.7) | 9.6 (2.3, 17.0) |
| Croatia | **Zagreb** | -20.1 (-30.1, -10.1) | -23.7 (-33.6, -13.9) | -21.7 (-31.7, -11.6) | -19.2 (-25.4, -13.0) | -13.1 (-20.8, -5.3) | -2.8 (-8.0, 2.5) | -9.0 (-15.9, -2.1) |
| Czechia | **Prague** | -10.1 (-14.3, -5.9) | -12.5 (-16.7, -8.3) | -10.8 (-15.0, -6.5) | -12.2 (-14.8, -9.5) | -9.1 (-12.0, -6.2) | -3.4 (-5.3, -1.4) | -9.0 (-11.7, -6.3) |
| Denmark | **Copenhagen** | -7.8 (-11.8, -3.9) | -10.0 (-14.0, -6.1) | -8.4 (-12.4, -4.3) | -5.0 (-7.4, -2.7) | -7.2 (-10.1, -4.2) | -6.2 (-8.3, -4.2) | -7.6 (-10.2, -5.0) |
| Estonia | **Tallinn** | -9.4 (-16.4, -2.4) | -12.7 (-19.8, -5.7) | -10.7 (-17.8, -3.6) | -9.9 (-14.1, -5.8) | -10.2 (-15.6, -4.7) | -4.7 (-8.4, -1.0) | -6.0 (-10.5, -1.5) |
| Finland | **Helsinki** | -6.7 (-9.3, -4.2) | -7.9 (-10.5, -5.4) | -6.7 (-9.2, -4.1) | -5.9 (-7.4, -4.4) | -5.6 (-7.6, -3.7) | -1.5 (-2.8, -0.2) | -1.5 (-3.1, 0.1) |
| France | **Paris** | -19.9 (-20.5, -19.2) | -21.8 (-22.4, -21.2) | -19.8 (-20.4, -19.2) | -15.6 (-16.0, -15.3) | -14.6 (-15.0, -14.2) | -8.5 (-8.8, -8.2) | -12.9 (-13.3, -12.5) |
| Germany | **Berlin** | -10.9 (-11.4, -10.4) | -13.7 (-14.2, -13.2) | -11.8 (-12.3, -11.3) | -10.5 (-10.8, -10.2) | -8.0 (-8.4, -7.7) | -2.9 (-3.2, -2.7) | -6.8 (-7.1, -6.4) |
| Greece | **Athens** | -15.5 (-23.0, -8.0) | -20.5 (-28.0, -13.1) | -19.7 (-27.4, -11.9) | -10.1 (-15.0, -5.3) | -16.3 (-22.7, -9.9) | -5.8 (-9.7, -2.0) | -8.5 (-13.1, -3.9) |
| Hungary | **Budapest** | -14.3 (-17.9, -10.7) | -17.4 (-21.0, -13.9) | -14.9 (-18.5, -11.2) | -14.8 (-17.1, -12.6) | -14.5 (-17.2, -11.9) | -4.6 (-6.4, -2.9) | -10.3 (-12.7, -7.9) |
| India | **Kolkata** | 7.2 (-2.4, 16.9) | 6.9 (-2.7, 16.6) | 6.0 (-3.7, 15.8) | 4.0 (-1.7, 9.6) | 8.2 (-0.4, 16.7) | -1.2 (-4.0, 1.6) | -0.3 (-3.8, 3.1) |
| Ireland | **Dublin** | -9.3 (-13.5, -5.2) | -10.1 (-14.2, -5.9) | -10.5 (-14.7, -6.3) | -4.3 (-6.7, -1.8) | -4.8 (-7.8, -1.9) | -2.5 (-4.6, -0.4) | -5.1 (-7.9, -2.4) |
| Israel | **Jerusalem** | -5.0 (-18.4, 8.4) | -7.0 (-20.3, 6.3) | -11.9 (-25.7, 1.8) | -4.5 (-12.4, 3.3) | -7.2 (-18.2, 3.9) | -7.0 (-13.4, -0.6) | -6.6 (-14.1, 0.9) |
| Italy | **Rome** | -15.1 (-17.8, -12.3) | -17.6 (-20.3, -14.9) | -17.0 (-19.8, -14.2) | -16.8 (-18.3, -15.2) | -12.1 (-14.4, -9.7) | -7.3 (-9.0, -5.7) | -9.9 (-11.8, -8.0) |
| Japan | **Tokyo** | 0.4 (-3.8, 4.6) | -1.8 (-5.9, 2.4) | -3.7 (-8.1, 0.6) | 6.4 (3.6, 9.3) | 5.5 (2.1, 8.8) | 3.1 (1.1, 5.2) | -1.6 (-4.2, 1.1) |
| Luxembourg | **Luxembourg** | -20.3 (-26.3, -14.3) | -23.1 (-29.1, -17.1) | -20.1 (-26.1, -14.1) | -13.9 (-17.5, -10.2) | -13.6 (-17.6, -9.6) | -6.4 (-9.0, -3.7) | -10.2 (-13.8, -6.6) |
| Mexico | **Mexico_City** | -5.3 (-20.1, 9.5) | -5.6 (-20.4, 9.3) | -6.7 (-21.6, 8.2) | 1.1 (-8.3, 10.5) | 4.6 (-6.8, 15.9) | -5.1 (-9.0, -1.2) | -3.1 (-8.6, 2.4) |
| Netherlands | **Amsterdam** | -14.7 (-17.3, -12.0) | -17.6 (-20.2, -15.0) | -16.1 (-18.7, -13.4) | -9.0 (-10.5, -7.5) | -10.6 (-12.4, -8.8) | -3.8 (-5.0, -2.6) | -7.9 (-9.6, -6.3) |
| New Zealand | **Auckland** | -13.4 (-20.3, -6.6) | -13.7 (-20.5, -6.9) | -12.7 (-19.5, -5.8) | -17.2 (-21.3, -13.1) | -7.1 (-12.1, -2.1) | -6.4 (-9.9, -2.9) | -7.4 (-11.8, -2.9) |
| Norway | **Oslo** | -8.4 (-12.1, -4.6) | -9.5 (-13.2, -5.7) | -8.2 (-12.0, -4.3) | 1.6 (-0.7, 3.8) | -4.6 (-7.4, -1.8) | -4.3 (-6.0, -2.6) | -6.5 (-8.7, -4.3) |
| Poland | **Warsaw** | -5.9 (-9.3, -2.5) | -9.4 (-12.8, -6.1) | -7.6 (-11.1, -4.2) | -8.5 (-10.7, -6.3) | -1.7 (-4.2, 0.8) | 0.7 (-0.9, 2.3) | -4.7 (-6.9, -2.5) |
| Portugal | **Lisbon** | -11.0 (-15.7, -6.2) | -12.2 (-16.9, -7.4) | -11.2 (-16.0, -6.4) | -10.4 (-13.3, -7.5) | -8.5 (-11.9, -5.2) | -2.0 (-3.8, -0.1) | -4.5 (-7.0, -2.0) |
| Romania | **Bucharest** | -8.2 (-14.0, -2.4) | -12.8 (-18.5, -7.1) | -8.7 (-14.6, -2.8) | -12.3 (-16.1, -8.5) | -9.9 (-14.5, -5.2) | -2.3 (-5.4, 0.8) | -3.3 (-7.2, 0.5) |
| Russia | **Moscow** | -15.7 (-24.0, -7.3) | -17.6 (-26.0, -9.2) | -16.9 (-25.3, -8.4) | -18.0 (-23.2, -12.8) | -11.4 (-17.4, -5.5) | -4.1 (-7.7, -0.5) | -5.5 (-10.2, -0.8) |
| Singapore | **Singapore** | 1.4 (-5.6, 8.5) | 0.6 (-6.4, 7.7) | 2.5 (-4.6, 9.6) | -3.7 (-8.2, 0.8) | 0.9 (-5.3, 7.1) | -1.5 (-2.6, -0.5) | -1.3 (-2.6, -0.0) |
| Slovakia | **Bratislava** | -18.6 (-25.9, -11.3) | -20.1 (-27.3, -12.8) | -19.7 (-27.1, -12.3) | -18.1 (-22.7, -13.5) | -8.5 (-13.8, -3.2) | -2.2 (-5.8, 1.4) | -9.8 (-14.6, -4.9) |
| South Korea | **Seoul** | n.a | n.a | n.a | n.a | n.a | n.a | n.a |
| Spain | **Madrid** | -15.2 (-18.1, -12.3) | -15.6 (-18.4, -12.7) | -16.2 (-19.1, -13.3) | -20.9 (-22.8, -19.1) | -16.0 (-18.2, -13.8) | -8.0 (-9.4, -6.6) | -9.0 (-10.8, -7.1) |
| Sweden | **Stockholm** | 0.1 (-2.6, 2.9) | -1.7 (-4.5, 1.0) | -0.1 (-2.8, 2.7) | -3.2 (-4.7, -1.6) | 0.9 (-1.2, 3.0) | -0.4 (-1.8, 1.0) | -1.6 (-3.3, 0.1) |
| Switzerland | **Zurich** | -17.5 (-19.0, -16.0) | -18.3 (-19.8, -16.8) | -18.2 (-19.7, -16.7) | -13.6 (-14.5, -12.7) | -10.7 (-11.8, -9.6) | -3.7 (-4.4, -2.9) | -8.1 (-9.0, -7.1) |
| Thailand | **Bangkok** | 5.4 (-3.5, 14.3) | 5.3 (-3.6, 14.2) | 5.8 (-3.1, 14.8) | -0.2 (-6.3, 6.0) | -11.8 (-21.1, -2.5) | 1.1 (-0.9, 3.2) | 2.2 (-0.3, 4.7) |
| Turkey | **Istanbul** | -19.6 (-34.8, -4.3) | -21.4 (-36.5, -6.2) | -20.7 (-36.2, -5.2) | -24.3 (-34.0, -14.5) | -18.5 (-30.8, -6.1) | -10.2 (-17.8, -2.7) | -6.3 (-15.6, 3.0) |
| United Arab Emirates | **Dubai** | -2.3 (-13.7, 9.1) | -1.3 (-12.6, 10.0) | -3.9 (-15.4, 7.5) | 2.2 (-4.9, 9.4) | -10.7 (-20.3, -1.0) | -3.0 (-6.6, 0.5) | -0.7 (-5.0, 3.6) |
| United Kingdom | **London** | -11.1 (-12.4, -9.8) | -12.7 (-14.0, -11.4) | -12.6 (-13.9, -11.3) | -12.5 (-13.2, -11.7) | -10.0 (-10.9, -9.1) | -5.4 (-6.0, -4.8) | -8.7 (-9.5, -7.9) |
| United States of America | **New York** | -3.9 (-6.1, -1.7) | -4.9 (-7.1, -2.7) | -4.0 (-6.3, -1.8) | -3.9 (-5.3, -2.5) | 0.1 (-1.5, 1.7) | 2.0 (1.1, 2.8) | -1.0 (-2.2, 0.1) |
|  | **Los Angeles** | -0.7 (-3.7, 2.4) | -1.6 (-4.6, 1.5) | 0.3 (-2.8, 3.4) | -2.7 (-4.4, -0.9) | 0.3 (-1.9, 2.6) | 0.9 (-0.4, 2.2) | -0.7 (-2.4, 1.0) |
|  | **Chicago** | -6.6 (-9.1, -4.1) | -7.5 (-10.0, -5.0) | -7.0 (-9.5, -4.4) | -1.2 (-2.9, 0.5) | -1.7 (-3.5, -0.0) | -1.4 (-2.3, -0.4) | -3.3 (-4.7, -2.0) |
|  | **Denver** | -1.2 (-6.8, 4.3) | -2.2 (-7.8, 3.3) | 0.1 (-5.5, 5.8) | 0.0 (-3.5, 3.6) | 0.9 (-3.1, 4.9) | -0.7 (-3.0, 1.6) | -2.5 (-5.5, 0.5) |
|  | **Phoenix** | -4.5 (-15.2, 6.3) | -5.6 (-16.3, 5.2) | -4.5 (-15.5, 6.4) | 0.4 (-6.3, 7.0) | -5.3 (-13.7, 3.0) | -2.5 (-6.9, 1.9) | -4.2 (-9.6, 1.3) |
|  | **Detroit** | -10.0 (-20.7, 0.8) | -11.5 (-22.2, -0.8) | -12.3 (-23.3, -1.3) | -4.9 (-12.4, 2.5) | -9.2 (-16.3, -2.2) | -1.5 (-5.6, 2.6) | -2.2 (-7.9, 3.4) |
|  | **Indiana** | -7.9 (-22.0, 6.2) | -10.6 (-24.6, 3.5) | -8.4 (-22.7, 5.9) | -1.7 (-11.9, 8.4) | -8.4 (-18.2, 1.3) | -0.8 (-6.0, 4.4) | -3.1 (-10.3, 4.1) |
|  | **Honolulu** | n.a | n.a | n.a | n.a | n.a | n.a | n.a |

*M1: daylight duration adjustment; M2 temporal control using a natural cubic spline of time (six degrees of freedom/year); M3: person-year-month vs. person-year-week. M4: exposure of interest* $f\left( x_{it}, l \right)$ *modelled using splines (4df) but no lag; M5: linear function; M7: linear function including lagged effect.*

**Table S9:** Associations between high temperature (99th vs 50^th,^ of the global distribution) and short sleep - RR (95%CI) - in the users of the smartwatch for each location based on alternative mode specifications.

| Country | **City** | **Main** | **M1** | **M2** | **M3** | **M4** | **M5** | **M6** |
| --- | --- | --- | --- | --- | --- | --- | --- | --- |
| Australia | **Sydney** | 1.33 (1.22, 1.46) | 1.35 (1.23, 1.47) | 1.33 (1.22, 1.46) | 1.26 (1.19, 1.33) | 1.32 (1.26, 1.39) | 1.19 (1.14, 1.24) | 1.20 (1.13, 1.28) |
|  | **Melbourne** | 1.74 (1.44, 2.08) | 1.75 (1.45, 2.09) | 1.74 (1.44, 2.08) | 1.48 (1.33, 1.64) | 1.41 (1.26, 1.58) | 1.36 (1.25, 1.48) | 1.58 (1.39, 1.79) |
|  | **Brisbane** | 1.36 (1.19, 1.55) | 1.37 (1.20, 1.56) | 1.33 (1.16, 1.52) | 1.38 (1.27, 1.49) | 1.30 (1.21, 1.41) | 1.15 (1.09, 1.21) | 1.15 (1.06, 1.25) |
|  | **Perth** | 1.04 (0.85, 1.25) | 1.09 (0.90, 1.31) | 0.98 (0.80, 1.18) | 1.14 (1.01, 1.28) | 1.10 (0.98, 1.25) | 1.18 (1.08, 1.28) | 1.13 (0.99, 1.28) |
|  | **Adelaide** | 1.22 (0.92, 1.60) | 1.23 (0.93, 1.61) | 1.26 (0.95, 1.65) | 1.37 (1.16, 1.61) | 1.28 (1.08, 1.51) | 1.16 (1.01, 1.32) | 0.92 (0.75, 1.13) |
| Austria | **Vienna** | 1.07 (1.00, 1.14) | 1.09 (1.02, 1.17) | 1.12 (1.04, 1.20) | 1.07 (1.03, 1.12) | 1.06 (1.01, 1.11) | 1.01 (0.99, 1.04) | 1.02 (0.98, 1.05) |
| Belgium | **Brussels** | 1.40 (1.28, 1.52) | 1.45 (1.34, 1.58) | 1.45 (1.33, 1.57) | 1.30 (1.24, 1.36) | 1.28 (1.21, 1.35) | 1.14 (1.09, 1.19) | 1.24 (1.17, 1.31) |
| Brazil | **Sao Paulo** | 1.71 (1.13, 2.39) | 1.71 (1.14, 2.40) | 1.66 (1.09, 2.35) | 1.28 (1.00, 1.60) | 0.90 (0.66, 1.22) | 1.11 (1.01, 1.23) | 1.21 (1.05, 1.39) |
| Bulgaria | **Sofia** | 1.64 (1.31, 2.03) | 1.70 (1.36, 2.09) | 1.62 (1.28, 2.01) | 1.38 (1.20, 1.58) | 1.51 (1.25, 1.80) | 1.28 (1.13, 1.45) | 1.48 (1.27, 1.71) |
| Canada | **Toronto** | 1.35 (1.19, 1.52) | 1.36 (1.20, 1.53) | 1.40 (1.23, 1.58) | 1.24 (1.16, 1.34) | 1.14 (1.05, 1.24) | 1.09 (1.03, 1.14) | 1.24 (1.16, 1.33) |
|  | **Vancouver** | 1.12 (0.83, 1.50) | 1.13 (0.83, 1.51) | 1.13 (0.83, 1.51) | 1.31 (1.11, 1.53) | 1.25 (1.01, 1.55) | 1.04 (0.90, 1.20) | 1.05 (0.86, 1.27) |
|  | **Edmonton** | 1.45 (1.05, 1.95) | 1.47 (1.07, 1.98) | 1.41 (1.02, 1.91) | 0.97 (0.79, 1.18) | 1.24 (0.99, 1.55) | 1.04 (0.93, 1.17) | 1.08 (0.93, 1.26) |
| China | **Shanghai** | 0.69 (0.47, 0.98) | 0.76 (0.52, 1.07) | 0.87 (0.59, 1.24) | 0.88 (0.72, 1.06) | 0.76 (0.56, 1.01) | 0.95 (0.80, 1.12) | 0.90 (0.72, 1.10) |
| Croatia | **Zagreb** | 1.51 (1.14, 1.94) | 1.66 (1.27, 2.10) | 1.58 (1.20, 2.02) | 1.55 (1.33, 1.79) | 1.38 (1.10, 1.69) | 1.01 (0.85, 1.18) | 1.10 (0.89, 1.35) |
| Czechia | **Prague** | 1.42 (1.24, 1.62) | 1.47 (1.28, 1.67) | 1.45 (1.26, 1.65) | 1.36 (1.26, 1.46) | 1.31 (1.19, 1.44) | 1.16 (1.09, 1.24) | 1.34 (1.22, 1.46) |
| Denmark | **Copenhagen** | 1.31 (1.14, 1.49) | 1.37 (1.20, 1.56) | 1.36 (1.19, 1.56) | 1.27 (1.18, 1.36) | 1.16 (1.05, 1.29) | 1.19 (1.10, 1.27) | 1.28 (1.17, 1.40) |
| Estonia | **Tallinn** | 1.02 (0.79, 1.30) | 1.09 (0.85, 1.39) | 1.07 (0.83, 1.38) | 1.47 (1.29, 1.66) | 1.06 (0.87, 1.27) | 0.99 (0.86, 1.14) | 1.05 (0.89, 1.24) |
| Finland | **Helsinki** | 1.20 (1.09, 1.31) | 1.23 (1.12, 1.35) | 1.22 (1.11, 1.34) | 1.29 (1.23, 1.36) | 1.18 (1.10, 1.27) | 1.05 (1.00, 1.11) | 1.08 (1.02, 1.15) |
| France | **Paris** | 1.53 (1.50, 1.56) | 1.57 (1.54, 1.60) | 1.54 (1.51, 1.57) | 1.46 (1.44, 1.48) | 1.37 (1.35, 1.39) | 1.23 (1.21, 1.24) | 1.35 (1.33, 1.37) |
| Germany | **Berlin** | 1.30 (1.27, 1.32) | 1.36 (1.34, 1.39) | 1.33 (1.30, 1.35) | 1.33 (1.32, 1.34) | 1.23 (1.21, 1.24) | 1.10 (1.10, 1.11) | 1.18 (1.17, 1.20) |
| Greece | **Athens** | 1.45 (1.19, 1.74) | 1.51 (1.25, 1.80) | 1.60 (1.32, 1.91) | 1.22 (1.08, 1.37) | 1.48 (1.25, 1.72) | 1.09 (0.97, 1.21) | 1.13 (0.99, 1.28) |
| Hungary | **Budapest** | 1.46 (1.31, 1.64) | 1.53 (1.37, 1.71) | 1.51 (1.35, 1.69) | 1.38 (1.29, 1.47) | 1.46 (1.35, 1.59) | 1.13 (1.07, 1.20) | 1.27 (1.17, 1.38) |
| India | **Kolkata** | 0.79 (0.61, 1.00) | 0.81 (0.63, 1.02) | 0.81 (0.63, 1.03) | 0.97 (0.86, 1.09) | 0.84 (0.67, 1.03) | 1.02 (0.96, 1.09) | 1.00 (0.92, 1.08) |
| Ireland | **Dublin** | 1.22 (1.06, 1.39) | 1.22 (1.06, 1.40) | 1.26 (1.10, 1.45) | 1.29 (1.19, 1.39) | 1.11 (1.01, 1.22) | 1.05 (0.98, 1.13) | 1.09 (0.99, 1.21) |
| Israel | **Jerusalem** | 1.48 (1.09, 1.93) | 1.51 (1.12, 1.95) | 1.60 (1.18, 2.07) | 1.20 (1.01, 1.41) | 1.42 (1.11, 1.77) | 1.23 (1.06, 1.42) | 1.27 (1.06, 1.50) |
| Italy | **Rome** | 1.40 (1.29, 1.52) | 1.45 (1.34, 1.57) | 1.45 (1.33, 1.57) | 1.38 (1.32, 1.44) | 1.32 (1.23, 1.41) | 1.19 (1.13, 1.26) | 1.26 (1.18, 1.34) |
| Japan | **Tokyo** | 1.03 (0.95, 1.11) | 1.05 (0.97, 1.13) | 1.07 (0.98, 1.15) | 0.95 (0.91, 1.00) | 0.95 (0.89, 1.01) | 0.93 (0.89, 0.97) | 1.02 (0.97, 1.07) |
| Luxembourg | **Luxembourg** | 1.61 (1.32, 1.94) | 1.67 (1.37, 2.01) | 1.59 (1.30, 1.93) | 1.54 (1.38, 1.71) | 1.48 (1.29, 1.68) | 1.18 (1.07, 1.29) | 1.27 (1.12, 1.45) |
| Mexico | **Mexico_City** | 1.06 (0.69, 1.56) | 1.09 (0.71, 1.60) | 1.09 (0.71, 1.60) | 0.99 (0.77, 1.24) | 0.89 (0.64, 1.21) | 1.11 (1.00, 1.23) | 1.00 (0.86, 1.16) |
| Netherlands | **Amsterdam** | 1.35 (1.22, 1.49) | 1.40 (1.27, 1.54) | 1.41 (1.28, 1.56) | 1.43 (1.35, 1.50) | 1.25 (1.17, 1.34) | 1.08 (1.02, 1.13) | 1.17 (1.10, 1.26) |
| New Zealand | **Auckland** | 1.48 (1.18, 1.84) | 1.51 (1.21, 1.87) | 1.50 (1.20, 1.86) | 1.39 (1.22, 1.56) | 1.30 (1.10, 1.52) | 1.04 (0.93, 1.17) | 1.02 (0.87, 1.19) |
| Norway | **Oslo** | 1.19 (1.06, 1.34) | 1.22 (1.08, 1.37) | 1.20 (1.07, 1.35) | 1.08 (1.01, 1.15) | 1.09 (1.00, 1.19) | 1.08 (1.02, 1.14) | 1.11 (1.03, 1.19) |
| Poland | **Warsaw** | 1.11 (1.00, 1.23) | 1.17 (1.06, 1.30) | 1.17 (1.05, 1.30) | 1.25 (1.18, 1.32) | 1.02 (0.95, 1.10) | 1.01 (0.96, 1.06) | 1.12 (1.05, 1.20) |
| Portugal | **Lisbon** | 1.22 (1.04, 1.41) | 1.25 (1.07, 1.45) | 1.22 (1.05, 1.42) | 1.22 (1.12, 1.33) | 1.20 (1.08, 1.34) | 0.99 (0.93, 1.05) | 1.05 (0.96, 1.14) |
| Romania | **Bucharest** | 1.28 (1.08, 1.50) | 1.37 (1.17, 1.60) | 1.30 (1.10, 1.53) | 1.34 (1.21, 1.47) | 1.28 (1.12, 1.45) | 1.08 (0.98, 1.19) | 1.15 (1.02, 1.28) |
| Russia | **Moscow** | 1.32 (1.04, 1.64) | 1.36 (1.08, 1.70) | 1.33 (1.05, 1.66) | 1.33 (1.17, 1.51) | 1.25 (1.06, 1.47) | 1.12 (1.01, 1.25) | 1.20 (1.04, 1.37) |
| Singapore | **Singapore** | 1.03 (0.83, 1.25) | 1.04 (0.84, 1.26) | 1.02 (0.83, 1.25) | 1.08 (0.96, 1.21) | 1.13 (0.94, 1.33) | 1.01 (0.98, 1.04) | 1.01 (0.97, 1.04) |
| Slovakia | **Bratislava** | 1.42 (1.13, 1.74) | 1.47 (1.18, 1.80) | 1.48 (1.19, 1.82) | 1.36 (1.20, 1.53) | 1.12 (0.95, 1.32) | 0.98 (0.87, 1.11) | 1.17 (1.00, 1.36) |
| South Korea | **Seoul** | n.a | n.a | n.a | n.a | n.a | n.a | n.a |
| Spain | **Madrid** | 1.38 (1.27, 1.50) | 1.40 (1.28, 1.51) | 1.39 (1.28, 1.51) | 1.49 (1.42, 1.56) | 1.39 (1.30, 1.48) | 1.19 (1.13, 1.24) | 1.24 (1.17, 1.31) |
| Sweden | **Stockholm** | 1.07 (0.98, 1.18) | 1.12 (1.02, 1.23) | 1.11 (1.01, 1.22) | 1.22 (1.16, 1.27) | 1.07 (1.00, 1.15) | 1.02 (0.97, 1.07) | 1.05 (0.99, 1.11) |
| Switzerland | **Zurich** | 1.48 (1.41, 1.55) | 1.50 (1.43, 1.57) | 1.51 (1.44, 1.58) | 1.38 (1.35, 1.42) | 1.31 (1.26, 1.35) | 1.13 (1.10, 1.15) | 1.21 (1.17, 1.25) |
| Thailand | **Bangkok** | 0.99 (0.74, 1.29) | 0.98 (0.74, 1.28) | 0.97 (0.72, 1.27) | 1.06 (0.89, 1.26) | 1.42 (1.09, 1.82) | 1.00 (0.94, 1.06) | 0.97 (0.90, 1.04) |
| Turkey | **Istanbul** | 0.94 (0.61, 1.36) | 0.97 (0.64, 1.41) | 0.89 (0.58, 1.32) | 1.23 (0.99, 1.51) | 1.20 (0.87, 1.58) | 1.04 (0.85, 1.26) | 0.85 (0.65, 1.10) |
| United Arab Emirates | **Dubai** | 0.92 (0.68, 1.21) | 0.89 (0.66, 1.18) | 0.93 (0.69, 1.23) | 1.00 (0.85, 1.17) | 1.16 (0.92, 1.44) | 1.11 (1.02, 1.21) | 1.04 (0.93, 1.15) |
| United Kingdom | **London** | 1.30 (1.24, 1.35) | 1.33 (1.27, 1.38) | 1.32 (1.27, 1.38) | 1.42 (1.39, 1.45) | 1.26 (1.22, 1.30) | 1.13 (1.11, 1.16) | 1.20 (1.17, 1.24) |
| United States of America | **New York** | 1.09 (1.02, 1.16) | 1.11 (1.04, 1.18) | 1.11 (1.04, 1.18) | 1.11 (1.07, 1.15) | 1.01 (0.96, 1.05) | 0.93 (0.91, 0.96) | 0.98 (0.94, 1.01) |
|  | **Los Angeles** | 0.89 (0.80, 0.97) | 0.89 (0.81, 0.98) | 0.89 (0.81, 0.98) | 1.01 (0.97, 1.06) | 0.88 (0.82, 0.94) | 0.94 (0.90, 0.97) | 0.98 (0.93, 1.04) |
|  | **Chicago** | 1.06 (0.98, 1.13) | 1.08 (1.00, 1.15) | 1.07 (0.99, 1.14) | 1.02 (0.98, 1.07) | 1.00 (0.95, 1.05) | 1.02 (0.99, 1.05) | 1.05 (1.01, 1.09) |
|  | **Denver** | 1.14 (0.96, 1.35) | 1.15 (0.97, 1.36) | 1.11 (0.93, 1.32) | 0.97 (0.88, 1.08) | 1.08 (0.96, 1.22) | 1.06 (0.99, 1.14) | 1.11 (1.01, 1.22) |
|  | **Phoenix** | 1.16 (0.85, 1.55) | 1.19 (0.87, 1.59) | 1.14 (0.83, 1.53) | 1.00 (0.84, 1.19) | 1.20 (0.94, 1.50) | 1.06 (0.93, 1.20) | 1.09 (0.92, 1.27) |
|  | **Detroit** | 0.83 (0.57, 1.19) | 0.87 (0.60, 1.24) | 0.89 (0.61, 1.28) | 1.02 (0.81, 1.27) | 1.04 (0.82, 1.31) | 1.01 (0.87, 1.16) | 0.96 (0.78, 1.17) |
|  | **Indiana** | 0.86 (0.52, 1.37) | 0.92 (0.56, 1.45) | 0.84 (0.51, 1.36) | 0.84 (0.60, 1.15) | 0.99 (0.71, 1.36) | 0.92 (0.77, 1.11) | 1.04 (0.81, 1.33) |
|  | **Honolulu** | n.a | n.a | n.a | n.a | n.a | n.a | n.a |

*M1: daylight duration adjustment; M2 temporal control using a natural cubic spline of time (six degrees of freedom/year); M3: person-year-month vs. person-year-week. M4: exposure of interest* $f\left( x_{it}, l \right)$ *modelled using splines (4df) but no lag; M5: linear function; M7: linear function including lagged effect.*


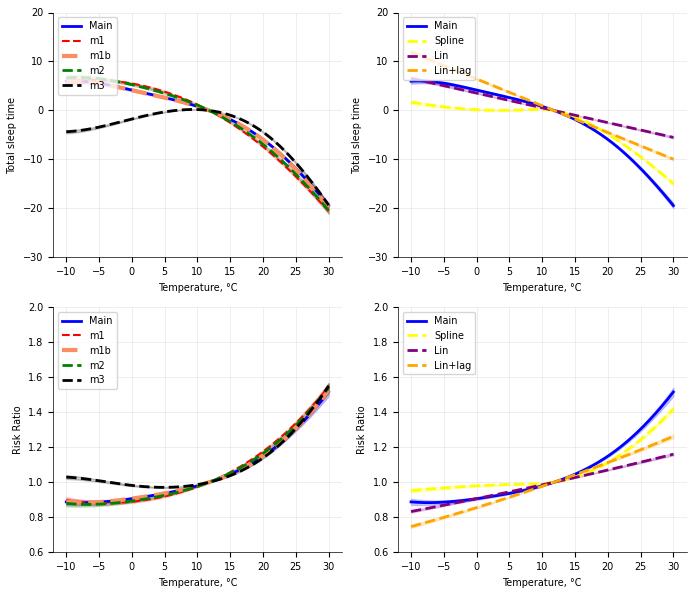


***Figure S11:*** *Associations between ambient temperature and sleep duration (****a*** *and* ***b****) and short sleep (****c****,* ***d****) for users of smartwatch for different model specifications. M1: daylight duration adjustment; M1b daylight and day of year adjustement; M2 temporal control using a natural cubic spline of time (six degrees of freedom/year); M3: person-year-month vs. person-year-week. M4: exposure of interest* $f\left( x_{it}, l \right)$ *modelled using splines (4df) but no lag; M5: linear function; M7: linear function including lagged effect.*

## Heatwaves and sleep loss


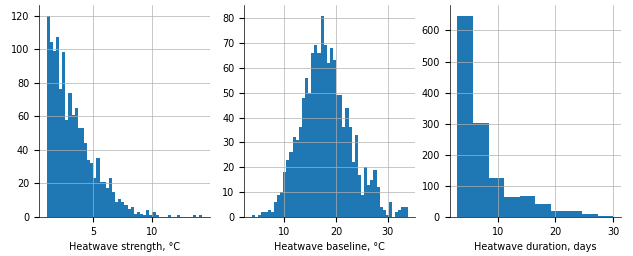


**Figure S12:** Heatwaves characteristics (strength, baseline temperature and duration) for the users of the under-mattress sensors.


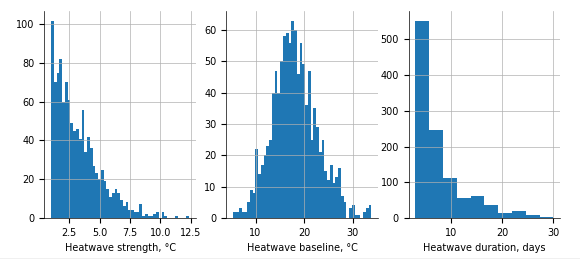


**Figure S13:** Heatwaves characteristics (strength, baseline temperature and duration) for the users of the smartwatch.


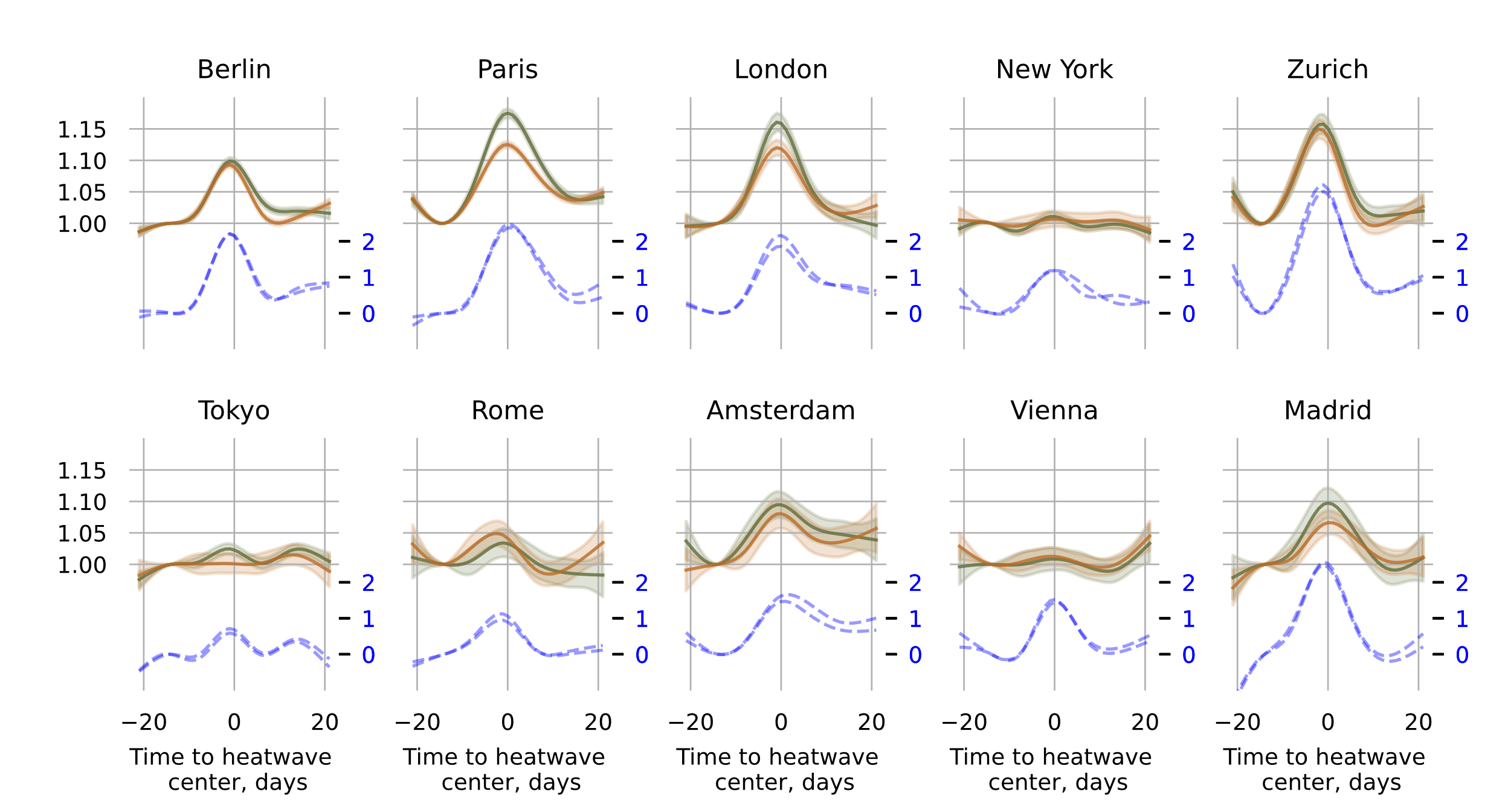


**Figure S14:** Increased temperature profiles during heatwaves (blue) and associated increased risk ratio of short sleep for under-mattress users (green) and smartwatch users (orange) for top 12 locations with most users and available heatwaves information. The number of participants in each city is available in Table S5.

## Sensitivity analyses

In the first sensitivity analysis, we used more conservative inclusion/exclusion criteria of at least 26, 52 or 104 weeks of data. This sensitivity analysis had a longer temporal resolution and coverage of expected seasonal effects within each participant. The number of nights and participants for each inclusion/exclusion criteria is available in Table S10. However, the results remained similar to the main using these additional inclusion/exclusion criteria (see supplementary Figure S15).

**Table S10:** Number of users and recordings for different inclusion/exclusion criteria.

|  | Under mattress | | Smartwatch | |
| --- | --- | --- | --- | --- |
| Criteria | Participants | Nights  (millions) | Participants | Nights  (millions) |
| 104 weeks | 56,312 | 53.9 | 48,911 | 41.0 |
| 52 weeks | 86,758 | 68.2 | 126,951 | 78.6 |
| 26 weeks | 103,727 | 71.9 | 171,488 | 88.9 |
| All | 116,879 | 73.1 | 200,879 | 91.5 |


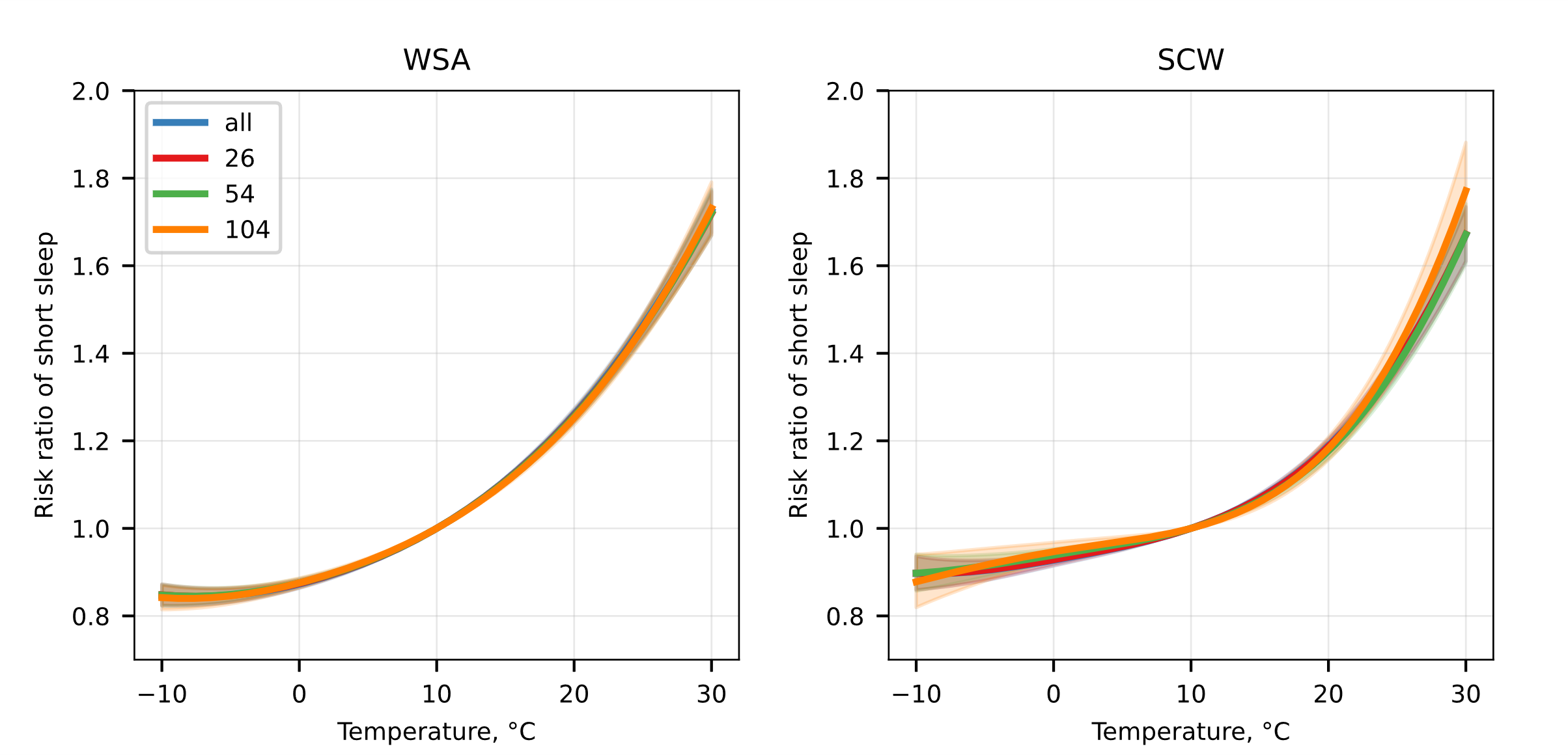


**Figure S15:** Risk ratio (95%CI) of short sleep, defined as sleep duration <6h for different ambient temperature for users using a smartwatch (right) or an under-mattress sensor (left). Different colors represent different cut-offs for the minimum number of weeks to be included in the analysis.

In the second sensitivity analysis, we adopt additional inclusion criteria based on minimum and maximum allowable sleep duration (between 4 and 12h) based on previous global observational studies on wearables sleep technologies^4,5^. These additional criteria removed ~3.4 million (5%) of nights for the users of the under-mattress sensor and ~2.7 millions of nights (3%) for the users of the watch. The results remained similar to the main analysis (see Figure S16).


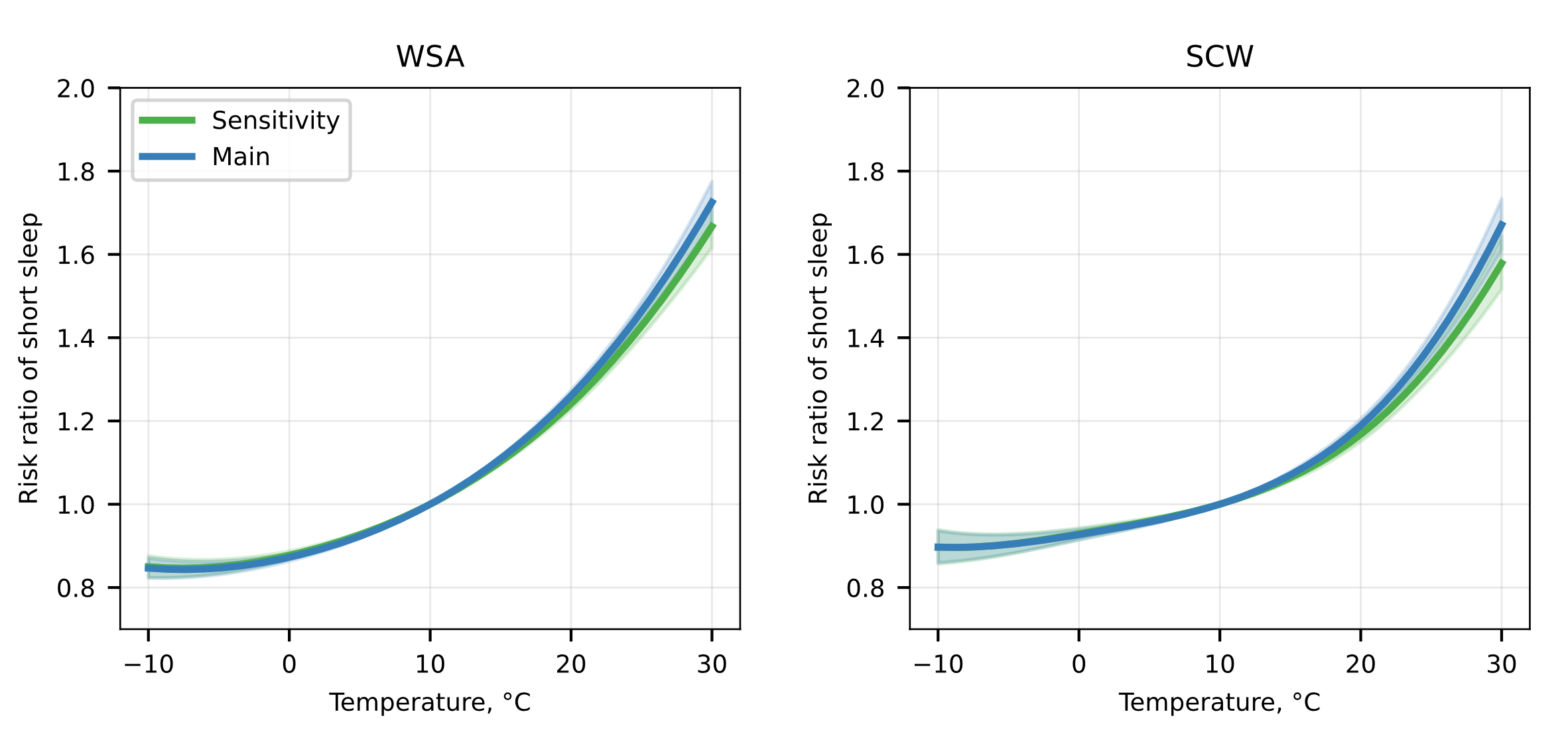


**Figure S16:** Risk ratio (95%CI) of short sleep, defined as sleep duration <6h for different ambient temperature for users using a smartwatch (right) or an under-mattress sensor (left). The analysis was run on the full dataset (blue) vs nights with sleep duration between 4 and 12h (orange – dashed lines).

The COVID19 pandemic impacted sleep, especially during lockdowns^6,7^. Therefore, in the third sensitivity analysis, we reproduced the analysis only in data after September 2022, a period where COVID19 was less likely to confound the observed results. In this analysis, we had ~30 million (115,229 users) and ~54 millions of nights (200,385 users) of the under-mattress sensor and smartwatch, respectively. The exposure-response curve between ambient temperature and short sleep was similar in this sensitivity analysis (Figure S17).


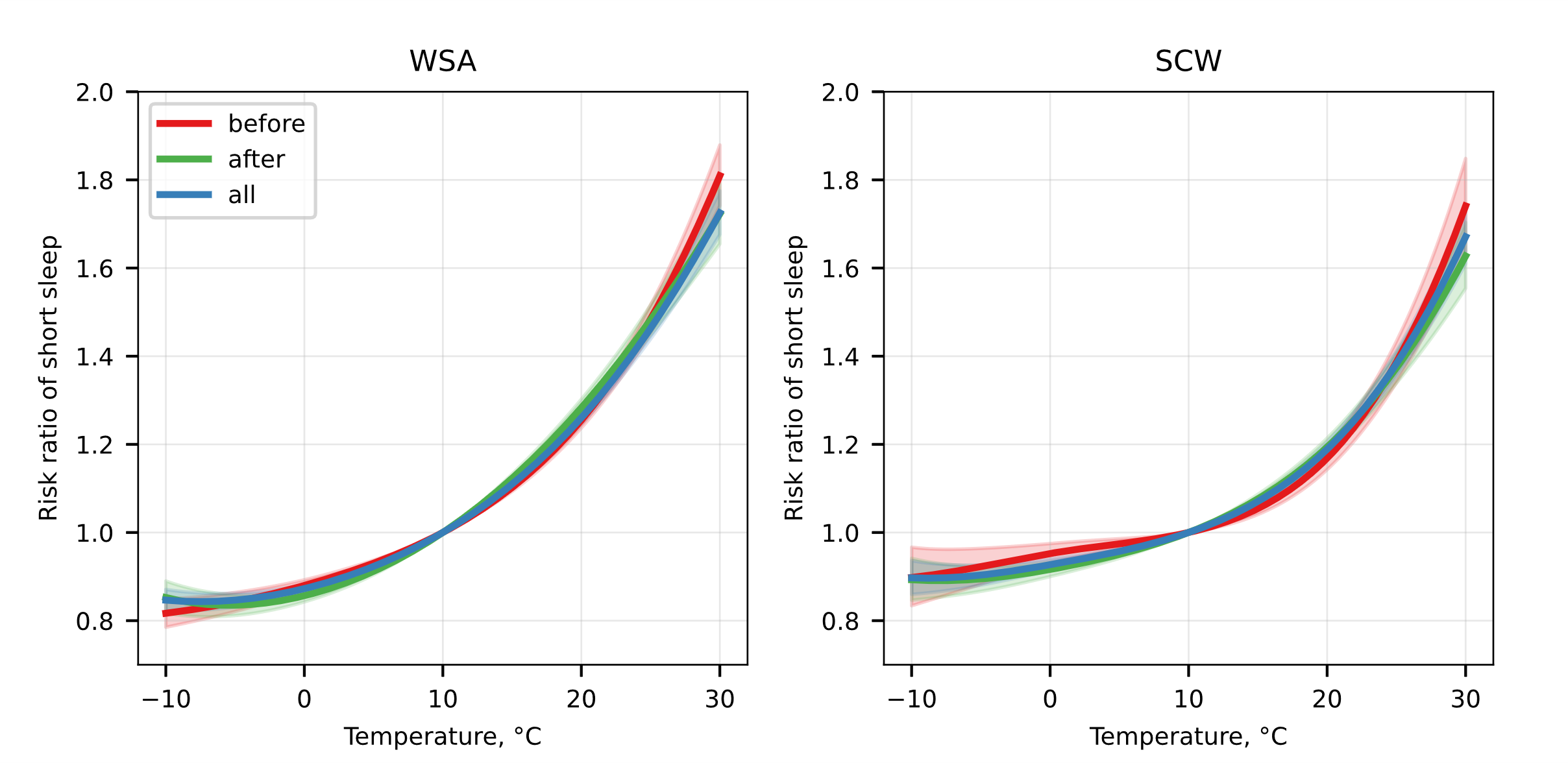


**Figure S17:** Risk ratio (95%CI) of short sleep, defined as sleep duration <6h for different ambient temperature for users using a smartwatch (right) or an under-mattress sensor (left). The analysis was run on the full dataset (blue) vs nights recorded after September (orange – dashed lines).

Air pollution and high ambient temperatures have been reported to be correlated^8,9^ and previous studies have suggested a potential synergistic effect between the two variables with health outcomes such as mortality^8,9^. Given this and the potential for multi-collinearity between the environmental variables, we repeated the analysis using a minimally adjusted model (adjusting only for the day of the year and the day of the week) in the top 12 locations with the most users. The association between temperature and short sleep was similar in fully adjusted vs minimally adjusted models (Supplementary Figure S18)


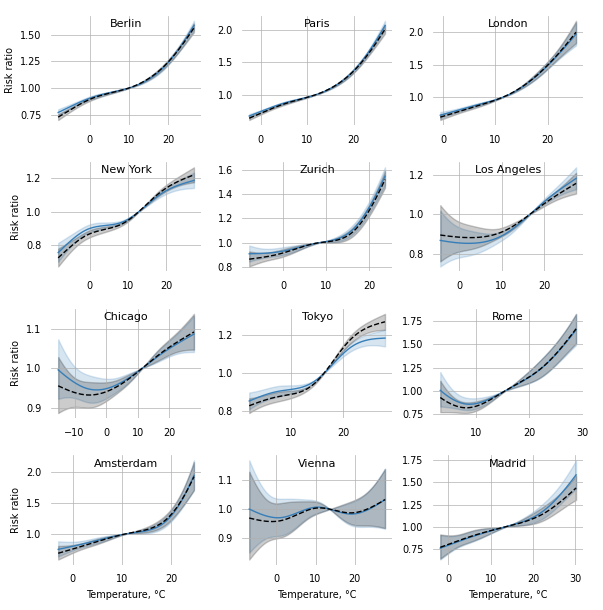


**Figure S18**: Risk ratio (95%CI) of short sleep, defined as sleep duration <6h, for different ambient temperature in the fully adjusted models (blue) and minimally adjusted models (black) for the top 12 countries with the most users. Different colors represent different cut-offs for the minimum number of weeks to be included in the analysis. The number of participants in each city is available in Table S5.


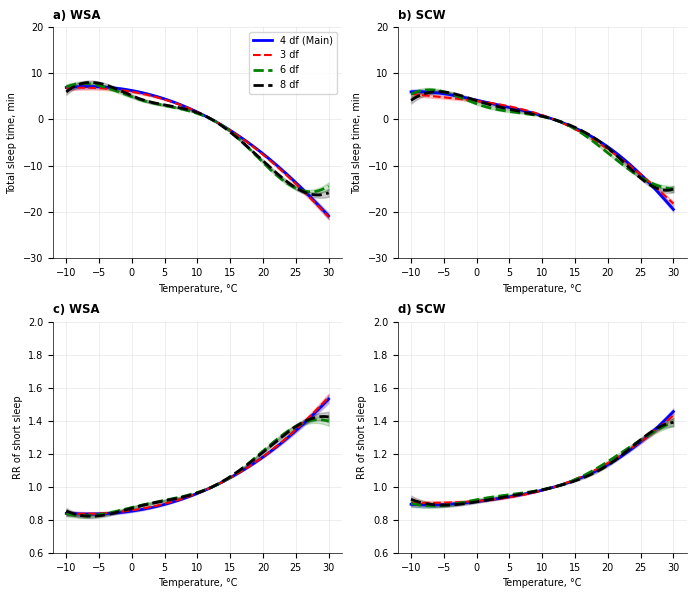


**Figure S19:** Associations between ambient temperature and sleep duration (a and b) and short sleep (c, d) for users of under-mattress sensors (left) and smartwatch (right) for different model specifications. The exposure of interest $f\left( x_{it}, l \right)$ was modelled using different degrees of freedom for the splines including 3df (red), 4df (main model – blue), 6 df and 8 df.

*
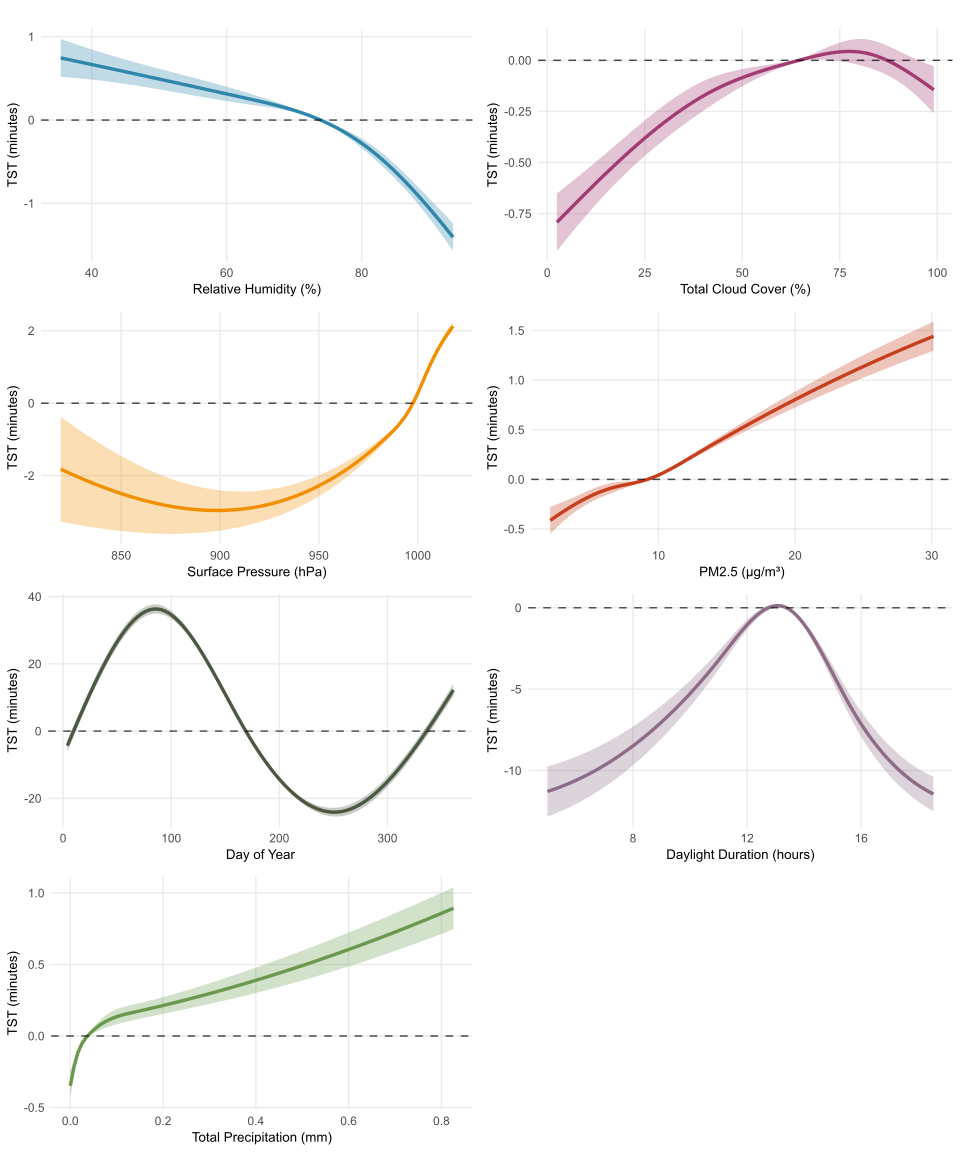
*

**Figure S20:** Exposure-response curve for the adjustment variables for the model investigating the association between temperature and sleep duration in users of the under-mattress sensors. Adjustment variables included relative humidity (4 df), total cloud cover (4 df), surface pressure (4 df), average daily density particulate matter with aerodynamic diameter <2.5 µg/m^3^ (4 df), splines of time (day of year variable; with 4 degrees of freedom [df]), daylight duration (4df – sensitivity model only) and total precipitation (4 df).


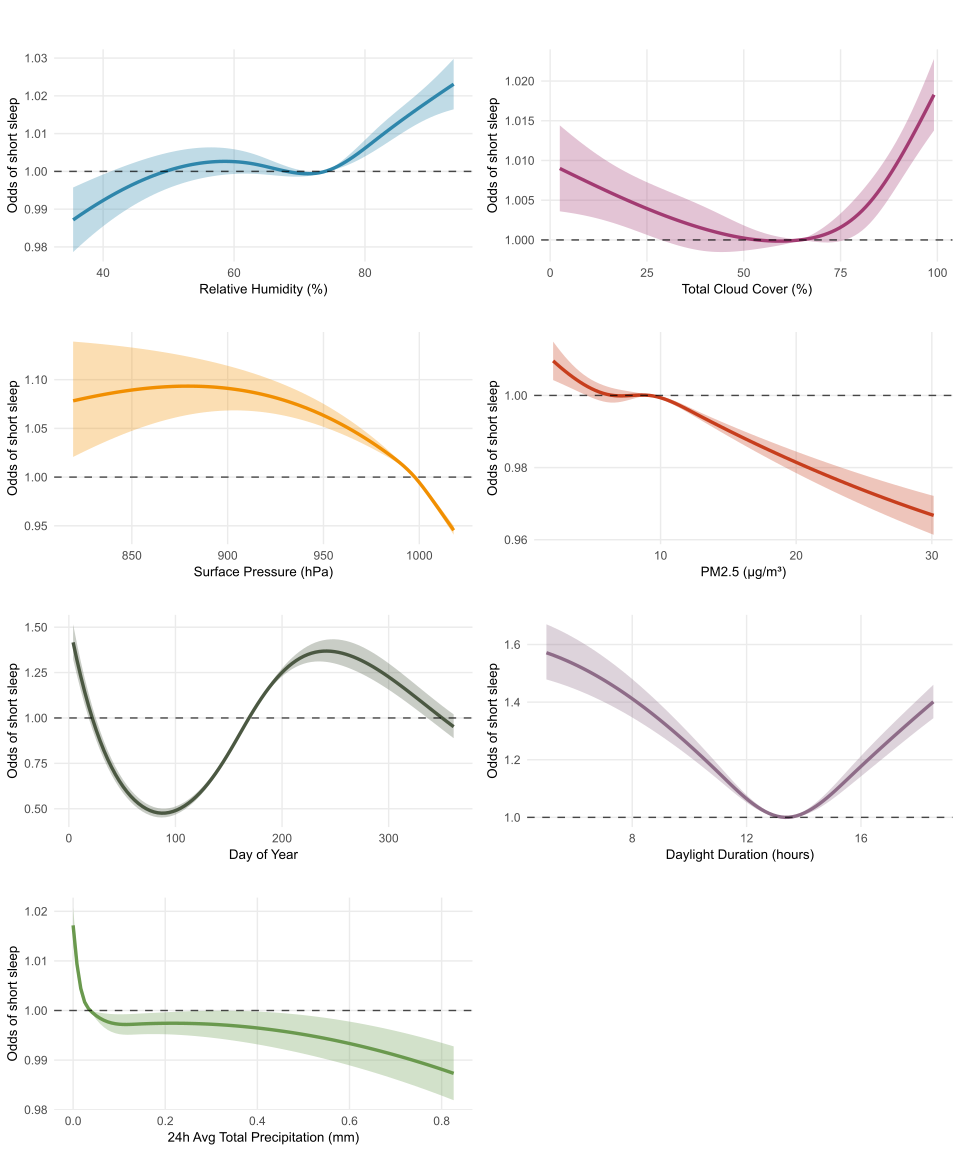


**Figure S21**: Exposure-response curve for the adjustment variables for the model investigating the association between temperature and short sleep (<6hr) in users of the under-mattress sensors. Adjustment variables included relative humidity (4 df), total cloud cover (4 df), surface pressure (4 df), average daily density particulate matter with aerodynamic diameter <2.5 µg/m^3^ (4 df), splines of time (day of year variable; with 4 degrees of freedom [df]), daylight duration (4df – sensitivity model only) and total precipitation (4 df).


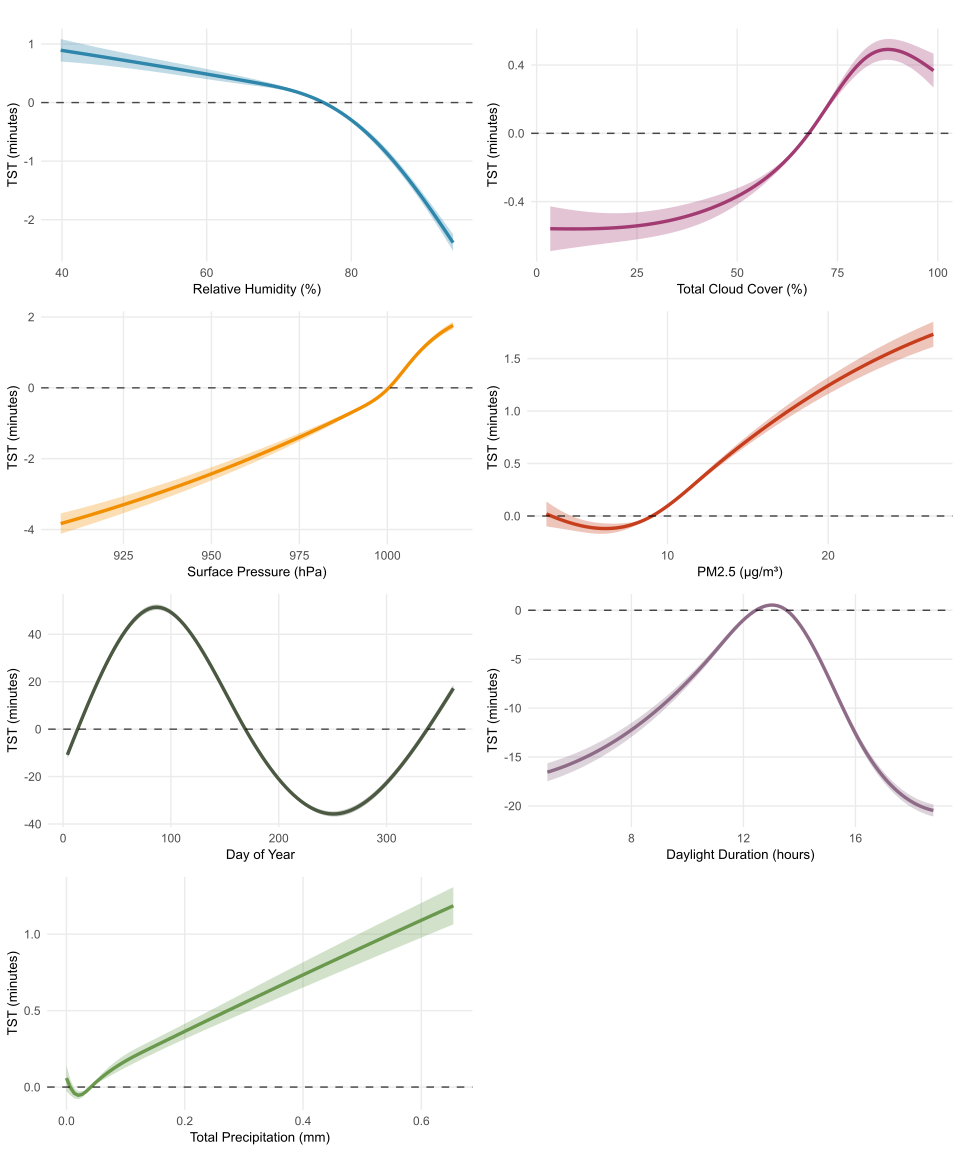


**Figure S22:** Exposure-response curve for the adjustment variables for the model investigating the association between temperature and sleep duration in users of the smartwatch. Adjustment variables included relative humidity (4 df), total cloud cover (4 df), surface pressure (4 df), average daily density particulate matter with aerodynamic diameter <2.5 µg/m^3^ (4 df), splines of time (day of year variable; with 4 degrees of freedom [df]), daylight duration (4df – sensitivity model only) and total precipitation (4 df).

*
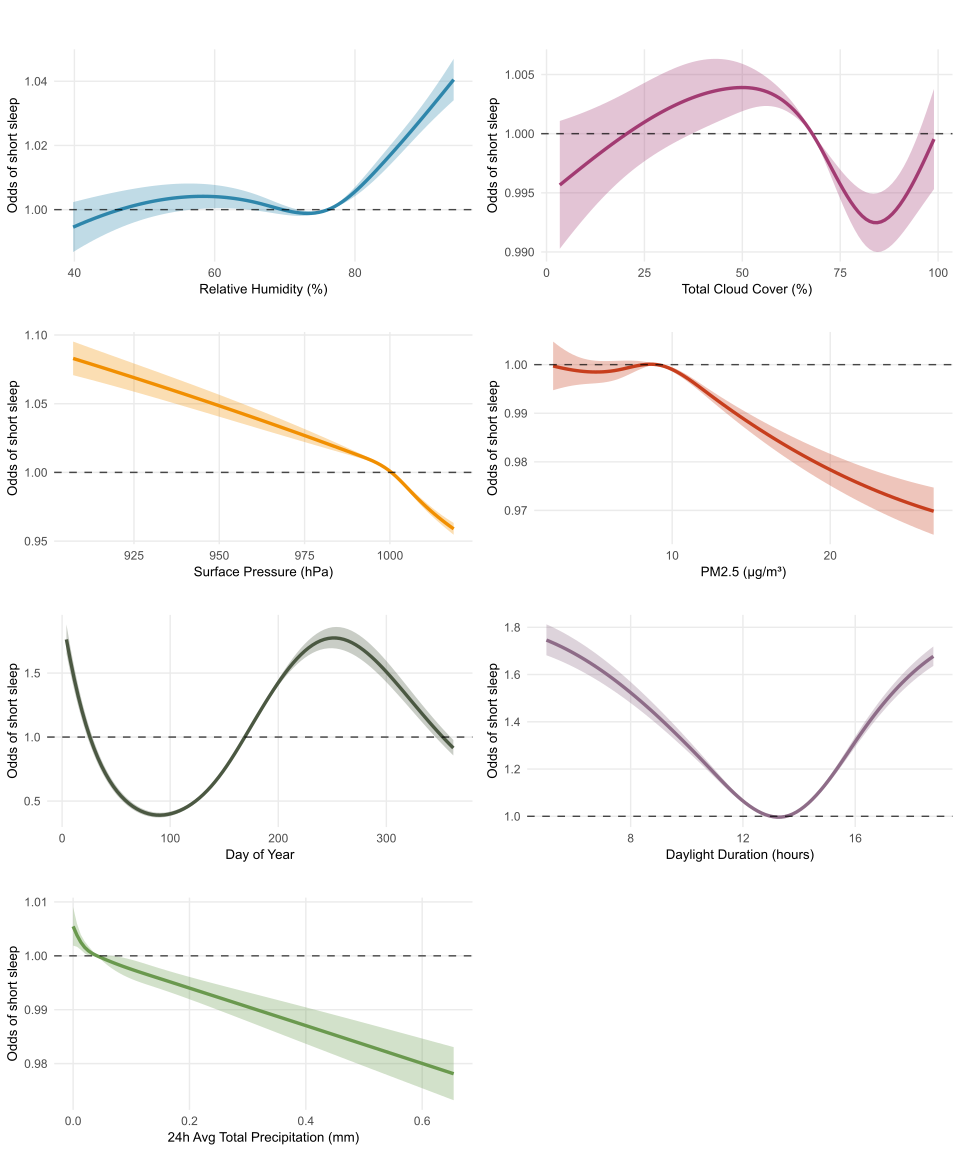
*

**Figure S23:** Exposure-response curve for the adjustment variables for the model investigating the association between temperature and short sleep (<6hr) in users of the smartwatch. Adjustment variables included relative humidity (4 df), total cloud cover (4 df), surface pressure (4 df), average daily density particulate matter with aerodynamic diameter <2.5 µg/m^3^ (4 df), splines of time (day of year variable; with 4 degrees of freedom [df]), daylight duration (4df – sensitivity model only) and total precipitation (4 df).

**References:**

1. Edouard P, Campo D, Bartet P, et al. Validation of the Withings Sleep Analyzer, an under-the-mattress device for the detection of moderate-severe sleep apnea syndrome. *J Clin Sleep Med* 2021; **17**(6): 1217-27.

2. Economics DA. The economic cost of sleep disorders in Australia, 2010: Sleep Health Foundation, 2011.

3. Scott H, Lechat B, Guyett A, et al. Sleep Irregularity Is Associated With Hypertension: Findings From Over 2 Million Nights With a Large Global Population Sample. *Hypertension* 2023; **80**(5): 1117-26.

4. Minor K, Bjerre-Nielsen A, Jonasdottir SS, Lehmann S, Obradovich N. Rising temperatures erode human sleep globally. *One Earth* 2022; **5**(5): 534-49.

5. Walch OJ, Cochran A, Forger DB. A global quantification of "normal" sleep schedules using smartphone data. *Sci Adv* 2016; **2**(5): e1501705.

6. Pepin JL, Bailly S, Mignot E, et al. Digital markers of sleep architecture to characterize the impact of different lockdown regimens on sleep health during the COVID-19 pandemic. *Sleep* 2022; **45**(6).

7. Pepin JL, Bailly S, Mordret E, et al. Greatest changes in objective sleep architecture during COVID-19 lockdown in night owls with increased REM sleep. *Sleep* 2021; **44**(9).

8. Rai M, Stafoggia M, de'Donato F, et al. Heat-related cardiorespiratory mortality: Effect modification by air pollution across 482 cities from 24 countries. *Environ Int* 2023; **174**: 107825.

9. Scortichini M, De Sario M, de'Donato FK, Davoli M, Michelozzi P, Stafoggia M. Short-Term Effects of Heat on Mortality and Effect Modification by Air Pollution in 25 Italian Cities. *Int J Environ Res Public Health* 2018; **15**(8).
